# Risk Factors for Death in 632 Patients with Sickle Cell Disease in the United States and United Kingdom

**DOI:** 10.1371/journal.pone.0099489

**Published:** 2014-07-02

**Authors:** Mark T. Gladwin, Robyn J. Barst, J. Simon R. Gibbs, Mariana Hildesheim, Vandana Sachdev, Mehdi Nouraie, Kathryn L. Hassell, Jane A. Little, Dean E. Schraufnagel, Lakshmanan Krishnamurti, Enrico Novelli, Reda E. Girgis, Claudia R. Morris, Erika Berman Rosenzweig, David B. Badesch, Sophie Lanzkron, Oswaldo L. Castro, James G. Taylor, Jonathan C. Goldsmith, Gregory J. Kato, Victor R. Gordeuk, Roberto F. Machado, on behalf of the walk-PHaSST Investigators and Patients

**Affiliations:** 1 Vascular Medicine Institute, University of Pittsburgh, Pittsburgh, Pennsylvania, United States of America; 2 Division of Pulmonary Allergy and Critical Care Medicine, University of Pittsburgh Medical Center, Pittsburgh, Pennsylvania, United States of America; 3 Columbia University, New York, New York, United States of America; 4 National Heart & Lung Institute, Imperial College London, London, United Kingdom; 5 Cardiovascular Branch, NHLBI, Bethesda, Maryland, United States of America; 6 Howard University, Washington, DC, United States of America; 7 University of Colorado HSC, Denver, Colorado, United States of America; 8 Case Western Reserve University, Cleveland, Ohio, United States of America; 9 University of Illinois, Chicago, Illinois, United States of America; 10 Children's Hospital of Pittsburgh, Pittsburgh, Pennsylvania, United States of America; 11 Johns Hopkins University, Baltimore, Maryland, United States of America; 12 Emory University School of Medicine, Atlanta, Georgia, United States of America; 13 National Heart Lung and Blood Institute/NIH, Bethesda, Maryland, United States of America; Vanderbilt University Medical Center, United States of America

## Abstract

**Background:**

The role of pulmonary hypertension as a cause of mortality in sickle cell disease (SCD) is controversial.

**Methods and Results:**

We evaluated the relationship between an elevated estimated pulmonary artery systolic pressure and mortality in patients with SCD. We followed patients from the walk-PHaSST screening cohort for a median of 29 months. A tricuspid regurgitation velocity (TRV)≥3.0 m/s cuttof, which has a 67–75% positive predictive value for mean pulmonary artery pressure ≥25 mm Hg was used. Among 572 subjects, 11.2% had TRV≥3.0 m/sec. Among 582 with a measured NT-proBNP, 24.1% had values ≥160 pg/mL. Of 22 deaths during follow-up, 50% had a TRV≥3.0 m/sec. At 24 months the cumulative survival was 83% with TRV≥3.0 m/sec and 98% with TRV<3.0 m/sec (p<0.0001). The hazard ratios for death were 11.1 (95% CI 4.1–30.1; p<0.0001) for TRV≥3.0 m/sec, 4.6 (1.8–11.3; p = 0.001) for NT-proBNP≥160 pg/mL, and 14.9 (5.5–39.9; p<0.0001) for both TRV≥3.0 m/sec and NT-proBNP≥160 pg/mL. Age >47 years, male gender, chronic transfusions, WHO class III–IV, increased hemolytic markers, ferritin and creatinine were also associated with increased risk of death.

**Conclusions:**

A TRV≥3.0 m/sec occurs in approximately 10% of individuals and has the highest risk for death of any measured variable.

**The study is registered in ClinicalTrials.gov with identifier:**

NCT00492531

## Introduction

As patients with sickle cell disease age, repetitive cycles of end-organ ischemia-reperfusion injury caused by vaso-occlusive events and intravascular hemolysis and anemia lead to end organ injury and failure [1], [2]. The development of pulmonary vascular disease and renal failure are particularly ominous. A series of studies using Doppler-echocardiography to estimate pulmonary artery systolic pressure has suggested that even mild elevations in estimated pulmonary pressures are associated with a significant increase in the risk of death [3]–[5]. Three clinical cohort studies have been recently published defining pulmonary hypertension (PH) by the gold standard, right heart catheterization [6]–[8]. These studies reported a prevalence of PH of 6–10.5% and in all cases the patients with PH exhibited an increased risk for early death. Despite the consistent findings of these echocardiographic cohort studies, and more recent right heart catheterization studies, the importance of PH as both a common complication observed in the adult sickle cell population and an attributable risk factor for death has been questioned in editorial forums [9], [10].

In the current study we estimate the prevalence of Doppler-echocardiography defined PH and the impact on survival in the largest screening cohort of patients with sickle cell disease, the Treatment of Pulmonary Hypertension and Sickle Cell Disease with Sildenafil Therapy (walk-PHaSST) study [11], [12]. This was designed both as a screening study to assemble a large cohort of patients with sickle cell disease and as an intervention trial to examine the effects of sildenafil therapy on PH. In addition to identifying the PH subjects eligible for the Main Intervention Trial (MIT), the screening study collected extensive data on demographic, medical history, physical examination, laboratory, and echocardiographic characteristics, and resulted in a large, multi-center cohort of over 600 patients with sickle cell disease. An observational follow-up study of screening study participants was also implemented as part of the original protocol, during which data on clinical outcomes, including deaths, were collected prospectively during a follow-up period of approximately two years. In this report, we present the results from this observational follow-up and an examination of mortality in the walk-PHaSST screening cohort and its association with various patient characteristics.

For our analysis of prevalence and hazards ratios for death we chose a conservative value for the tricuspid regurgitation velocity of ≥3.0 m/sec. This value had a 67% positive predictive value for PH measured by right heart catheterization (defined by a mean pulmonary artery pressure of ≥25 mm Hg) in the French screening study published by Parent and colleagues [6], and had a 77% positive predictive value for PH in the NIH-pulmonary hypertension screening study [8]. This TRV value provides a more conservative population estimate of Doppler-defined PH prevalence and impact on mortality than a cut-off value of 2.5 m/sec. We recognize that the gold-standard for PH diagnosis is a right heart catheterization, but this was not considered feasible for the large number of patients enrolled in this NIH funded trial.

## Methods

### Study Design and Selection of Subjects

The protocol for this trial and supporting CONSORT checklist are available as supporting information; see Checklist S1 and Protocol S1.The study population and design have been described in detail elsewhere [11], [12]. In brief, we analyzed all members of the screening cohort for whom follow-up data were available. Local institutional review boards or ethics committees (University of Pittsburgh, Columbia University, National Heart & Lung Institute, Imperial College London, Howard University, University of Colorado, Denver, University of Illinois Chicago, Johns Hopkins University, National Heart Lung and Blood Institute/NIH) approved the protocol and written informed consent was obtained (ClinicalTrials.gov identifier NCT00492531). Written informed consent was obtained from patients or their guardians in the case of minors. Overall, we recruited 720 subjects age 12 and over at steady state from nine different study sites in the United States and one site in the United Kingdom. Of these, 632 (94.2%) were followed for mortality and were included in our analysis; over a median of 29 months, we observed 22 deaths. Deaths were reported by study site coordinators and verified by review of medical records, contact with next-of-kin, and/or death certificates when available.

### Evaluation of Subjects

All screening study subjects were evaluated by histories of clinical events and lifetime treatments, physical examination, laboratory screening, transthoracic Doppler echocardiography, and the six-minute walk test. Routine laboratory tests (complete blood count, serum chemistry profile, and lactate dehydrogenase) from samples taken at the subject's screening visit were performed in the local laboratories of the participating institutions. Echocardiography was performed at the participating institutions and read centrally in the NHLBI echocardiography core laboratory. Percentage of hemoglobin F was measured by high-performance liquid chromatography (HPLC) (Ultra Resolution System, Trinity Biotech). Alpha-thalassemia was detected by molecular methodology based on polymerase chain reaction at the University of Pittsburgh. Serum N-terminal pro-brain natriuretic peptide (NT-pro BNP) concentration was measured by a sandwich immunoassay using polyclonal antibodies that recognize epitopes located in the N-terminal segment (1–76) of pro-BNP (1–108) (Elecsys analyser; Roche Diagnostics, Mannheim, Germany), as previously described [13]. Ferritin was measured with an enzyme immunoassay (Ramco Laboratories Inc, Stafford, TX; reference range, 20–300 ng/mL).

### Statistical Analysis

Patient characteristics are presented as median and interquartile range (IQR) or number and percentage of participants with a given characteristic. TRV was categorized into two groups, ≥3.0 m/sec and below 3.0 m/sec, based on now established high PPV of this cut-off for PH defined by right heart catheterization; NT-proBNP was categorized into two groups based on a cut-off value of 160 pg/mL. A hemolytic component variable was derived using principal component analysis from four markers of hemolysis (lactate dehydrogenase, aspartate aminotransferase, total bilirubin, and reticulocyte percent), as described elsewhere [14], and divided into two groups based on the 75^th^ percentile. Composite variables combining correlated risk factors were derived to minimize the effects on risk estimates of entering collinear variables simultaneously into statistical models. Associations of patient characteristics with mortality were assessed using Cox proportional hazards regression analysis, Kaplan-Meier survival curves, and the log-rank test for differences across groups. Continuous variables were log-transformed as necessary to normalize skewed distributions. Patients were censored at the point of their last contact with study staff if they did not have an event. Time to event or censoring was measured from the date of entry into the screening study. Regression coefficients were tested for significant differences from zero by the Wald test. The proportional hazards assumption was evaluated by testing the significance of each variable entered into the model as a time dependent covariate. For the final model, variables were entered in a stepwise approach if they had a significant univariate association with mortality. All statistical analyses were performed using PROC MEANS in SAS, version 9.1 (SAS Institute, Inc., Cary, NC) and STCOX and STS GRAPH in Stata, version 11.1 (Statacorp, LP, College Station, TX).

## Results

### Characteristics of the walk-PHaSST Screening Cohort

**Figure 1:**
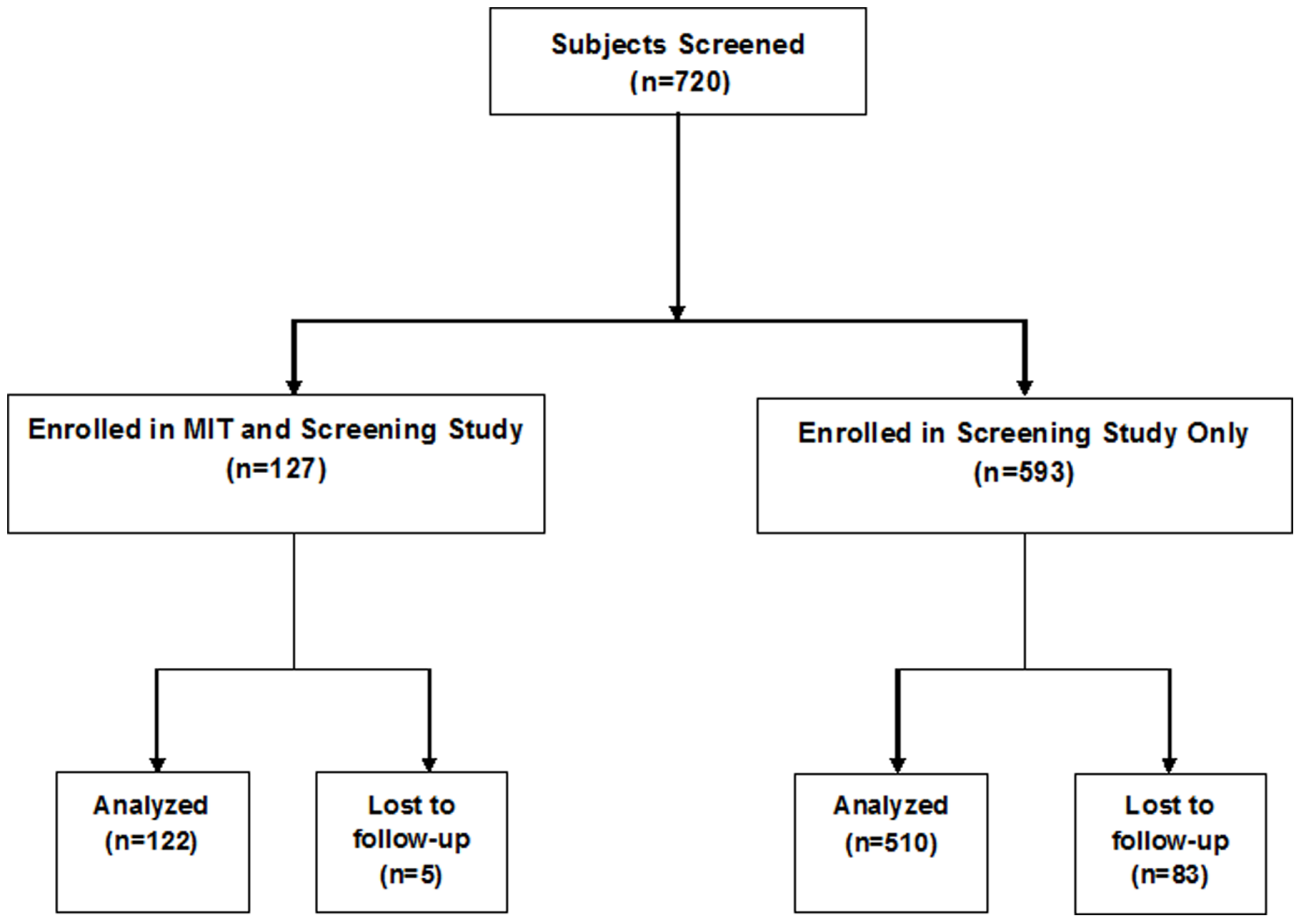
Study Flowchart.

Demographic, clinical, laboratory, and echocardiographic characteristics of the screening cohort are shown in **Table 1** **and** **Figure 1**. Of the 632 participants, 47% were male and 74% were homozygous for the hemoglobin S mutation. Study participants ranged in age from 12 to 84 years, and the median age was 37 years. A total of 22 deaths were observed during a median follow-up time of 29 months.

**Table 1:** Characteristics of Patients in Screening Cohort.

	Total	Median(IQR)^1^
**Demographics, Genotype, and Vital Status**		
Age,years	632	37 (26–47)
Male, N(%)	632	294 (46.5)
SS Genotype, N(%)	632	466 (73.7)
Deaths, N(%)	632	22 (3.5)
Follow-up time, months	632	29.0 (25.1–33.4)
**Clinical and Echocardiographic Measures**		
Hydroxyurea, current use, N(%)	632	238 (37.7)
Chronic Transfusions, N(%)	627	76 (12.1)
O2 Sat, %	625	97 (95–99)
Systolic Blood Pressure, mm Hg	628	118 (109–129)
Diastolic Blood Pressure, mm Hg	628	69 (62–75)
Six Minute Walk Distance, m	618	436 (378–500)
TRV, m/sec	572	2.5 (2.3–2.7)
**Laboratory Measures**		
BNP, pg/mL	582	67.9 (29.0–155.0)
Ferritin, ng/mL	576	228.8 (93.2–520.8)
Fetal Hemoglobin, %	566	4.8 (1.5–10.6)
Hemolytic Component, relative unit	546	0.09 (−1.20–1.29)
Absolute Reticulocyte Count, ×10^6^/µL	594	217.6 (139.0–320.1)
Reticulocytes, %	587	7.7 (4.2–11.8)
Hemoglobin, g/dL	615	9.2 (8.0–10.7)
Hematocrit, %	616	26.8 (22.9–31.0)
MCHC, g/dL	612	34.6 (33.6–35.7)
MCV, µm^3^	614	89.2 (81.6–98.1)
Platelets, ×10^3^/µL	614	341 (262–430)
RBC, ×10^6^/µL	615	2.95 (2.41–3.67)
WBC, ×10^3^/µL	615	9.2 (7.0–11.9)
Albumin, g/dL	615	4.2 (3.9–4.4)
Alkaline Phosphatase, U/L	615	86 (67–118)
ALT, U/L	619	22 (16–32)
AST, U/L	604	39 (27–54)
BUN, mg/dL	616	10.0 (7.0–28.0)
Creatinine, mg/dL	620	0.7 (0.6–0.9)
LDH, IU/L	584	367 (250–555)
Total Bilirubin, mg/dL	618	2.3 (1.4–3.6)

1 Unless otherwise indicated.

**Figure 2:**
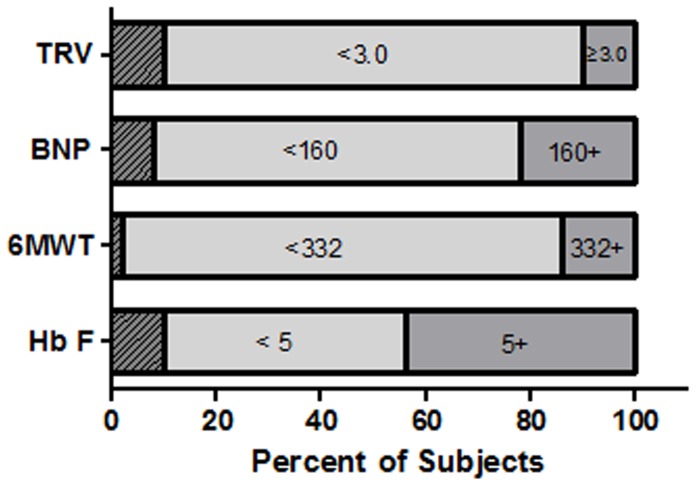
Prevalence of TRV, BNP, Six Minute Walk Distance, and Fetal Hemoglobin. The screening study patient population consisted of 671 patients with sickle cell anemia, 632 of whom were followed for mortality over a median of 29 months. Ten percent (n = 64) had TRV measurements of 3.0 m/sec or higher and 80% (n = 508) had measurements less than 3.0 m/sec. TRV measurements were not available for 10% (n = 60) of the patient population (diagonal stripes). Twenty-two percent of patients (n = 140) had BNP measurements of 160 pg/mL or higher. Fourteen percent (n = 85) had six minute walk distances less than 332 meters, and 46% (n = 289) had fetal hemoglobin levels less than 5%.

The prevalence of characteristics that are generally thought to be markers of poor relative health in patients with sickle cell disease is shown in **Figure 2**. Sixty-four patients (10.1%) had TRV measurements of 3.0 m/sec or higher, and140 (22.2%) had NT proBNP measurements at least as high as 160 pg/mL, a previously validated cut-off value associated with both PH and mortality in patients with sickle cell disease [15], [16]. Thirty-nine (6.2%) patients had both TRV≥3.0 m/sec and NT-proBNP≥160 pg/mL. Walk distances of less than 332 meters were observed in 85 (13.5%) patients, and fetal hemoglobin levels less than 5% were observed in 289 (45.7%) patients.

### Univariate Associations with Mortality

Patient characteristics and their associations with mortality were analyzed with Cox proportional hazards regression analysis (**Table 2**). In this study, an increased risk of death was observed for both age and gender, with males at two and a half times the risk of dying relative to females (p = 0.05), and patients older than 47 years at twice the risk of dying compared with patients less than 26 years (p = 0.03). Associations with mortality were also observed for chronic transfusions (p = 0.02) and a NYHA/WHO class value or III or IV (p = 0.01). Variables not associated with mortality included current hydroxyurea use, SC genotype, self-reported history of painful episodes, and six-minute walk distance.

**Table 2:** Cox Proportional Hazards Regression Analysis of Mortality for Demographic, Clinical, and Laboratory Characteristics.

Characteristic	Category	Total	Deaths	Hazard Ratio (95% CI)^1^	p
Age, years	-----	632	22	2.02 (1.1–3.8)	0.032
Gender	F	338	7	1.0	
	M	294	15	2.48 (1.0–6.1)	0.048
Genotype	SS	446	17	1.0	
	SC	166	5	0.83 (0.3–2.2)	0.71
Hydroxyurea use	none/past	394	11	1.0	
	current	238	11	1.64 (0.7–3.8)	0.25
Chronic Transfusions	no	551	15	1.0	
	yes	76	6	3.00 (1.2–7.7)	0.023
Moderate/Severe Pain episodes in past year	0–2	209	7	1.0	
	>2	420	14	1.02 (0.4–2.5)	0.96
Systolic BP, mm Hg	----	628	21	1.62 (0.93–2.8)	0.086
TRV, m/sec	<3.0	508	10	1.0	
	3.0+	64	11	9.55 (4.1–22.5)	<0.001
BNP, pg/mL^2^	---	582	19	2.56 (1.8–3.6)	<0.001
BNP, pg/mL	<160	442	8	1.0	
	≥160	140	11	4.55 (1.8–11.3)	0.001
Hemolytic Component, relative unit	<1.28	412	9	1.0	
	≥1.28	134	10	3.43 (1.4–8.4)	0.007
Lactate dehydrogenase, IU/L^3^	----	584	19	1.68 (1.1–2.6)	0.021
Aspartate aminotransferase, IU/L^3^	----	604	21	1.91 (1.3–2.7)	<0.001
Reticulocytes, %^2^	----	585	20	1.07 (0.6–2.0)	0.83
Total bilirubin, mg/dL^2^	----	618	21	1.15 (0.7–2.0)	0.63
Six-Minute Walk, m	≥332	533	19	1.0	
	<332	85	3	1.01 (0.3–3.4)	0.99
NYHA/WHO Class	I	439	11	1.0	
	II	141	7	1.96 (0.8–5.1)	0.17
	III,IV	35	4	4.52 (1.4–14.3)	0.010
Hemoglobin, g/dL	----	615	21	0.72 (0.4–1.4)	0.31
White blood cell count, ×10^3^/µL^2^	----	615	21	1.55 (0.8–2.9)	0.175
Absolute neutrophil count^2^	----	605	21	1.34 (0.8–2.3)	0.28
Platelets, ×10^3^/µL^2^	----	614	21	0.65 (0.4–1.0)	0.050
Hemoglobin F, %^2^	----	537	16	0.72 (0.3–1.5)	0.39
BUN, mg/dL^2^	----	616	20	1.24 (0.7–2.3)	0.49
Creatinine, mg/dL^2^	----	620	21	1.74 (1.4–2.2)	<0.001
Albumin, g/dL	----	615	20	0.60 (0.4–0.8)	0.002
Alkaline phosphatase, U/L^2^	----	615	21	1.88 (1.2–3.0)	0.009
Alanine aminotransferase, U/L^2^	----	619	21	1.68 (1.1–2.6)	0.023
Ferritin, ng/mL^2^	----	576	21	2.58 (1.5–4.4)	<0.001

1 Hazard ratios presented for 75^th^ relative to the 25^th^ percentile, unless otherwise indicated. All results are unadjusted.

2 Transformed using the log or square root function.

3 Adjusted for site-specific differences in normal ranges.

**Figure 3:**
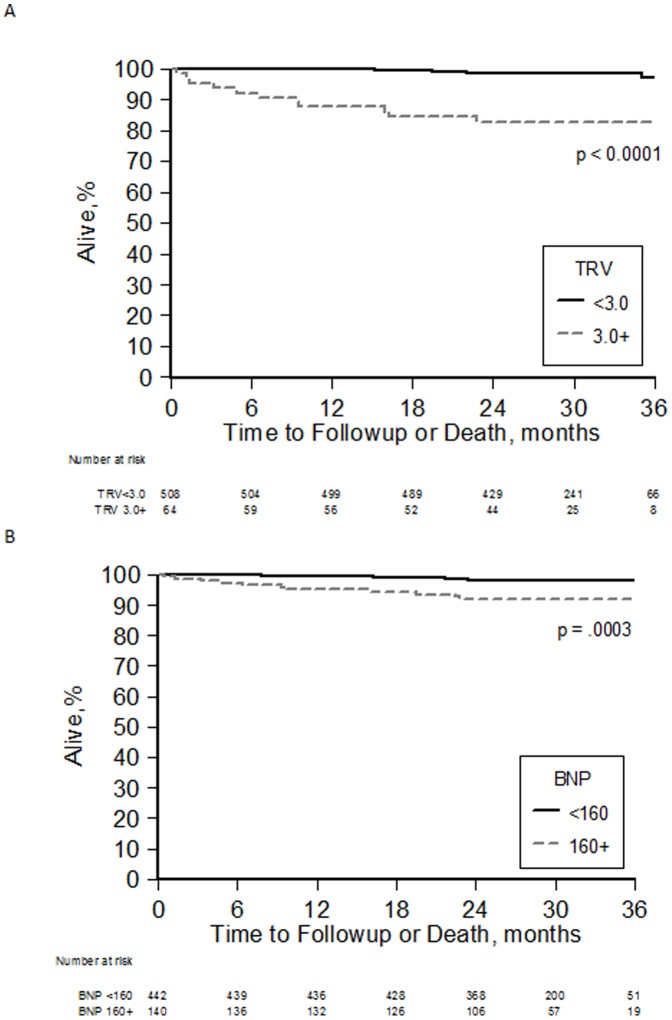
Kaplan-Meier Analysis of Survival Time by TRV and BNP. Longer survival times were observed for a) subjects with TRV less than 3.0 m/sec (p<0.0001) and b) for subjects with BNP levels less than 160 pg/mL (p = 0.0003).

We also observed associations with mortality for two separate biomarkers of pulmonary hypertension, TRV and NT-proBNP, both of which were identified as risk factors for death in other cohorts [3]–[5], [13]. At 24 months the cumulative survival was 83% for patients with TRV measurements of 3.0 m/sec or greater and 98% for patients below 3.0 m/sec (p<0.0001; **Figure 3a**). Similarly, the cumulative survival was lower for patients with NT-proBNP levels of 160 pg/mL or higher compared with levels less than 160 pg/mL, although the magnitude of the difference was not as large (92% for NT-proBNP≥160 vs. 98% for NT-proBNP<160, log-rank p = 0.0003; **Figure 3b**). The unadjusted hazard ratios for death were 11.14 (95% CI 4.1–30.1; p<0.0001) for patients with TRV≥3.0 m/sec relative to TRV<2.7 m/sec and 4.55 (95% CI 1.8–11.3; p = 0.001) for patients with NT-proBNP≥160 pg/mL relative to NT-proBNP levels < 160 (**Table 2**). For log-transformed NT-proBNP, the risk ratio for death was 2.56 (95% CI 1.8–3.6; p<0.0001) for NT-proBNP levels in the 75^th^ percentile relative to NT-proBNP levels in the 25^th^ percentile (**Table 2**).

Other variables associated with mortality in our cohort included the calculated hemolytic component and two of the variables from which it was calculated, aspartate aminotransferase (AST) and lactate dehydrogenase, as well as ferritin and creatinine. Patients with a hemolytic component value at least as large as 1.28, the 75^th^ percentile in this dataset, were at more than three times the risk of dying relative to those patients with smaller values (HR = 3.43, 95% CI 1.4–8.4; p = 0.007) (**Table 2**).

### Multivariate Associations with Mortality

In stepwise multiple proportional hazards regression analysis, the magnitude of the association between TRV and mortality was decreased but still significant after adjustment for ferritin, AST, creatinine and NT-proBNP (HR 4.27, 95%CI 1.3–14.1; p = 0.04; **Table 3**). Comparing the 75^th^ with the 25^th^ percentiles of log-transformed values, ferritin (HR 1.80, 95% CI 1.0–3.1; p = 0.04) was also associated with mortality after adjustment for all other variables in the model. AST was associated with mortality in the multivariate model at the α = 0.10 level (HR 1.84; 95%CI 0.95–3.5; p = 0.07), as were creatinine and NT-proBNP, which were entered into the model as a composite variables due to their high pairwise correlation (Pearson r = 0.48, p<0.0001). Patients who had both a high creatinine and a high NT-proBNP were at almost 3 times the risk of dying compared with patients with lower levels (HR 2.71, 95% CI 0.9–6.2; p = 0.08).

**Table 3:** Multivariate Cox Proportional Hazards Regression Analysis of Mortality.

Risk Factor	Category	N at Risk (deaths)	Hazard Ratio (95% CI)^1^	p
TRV, m/sec	<3.0	445 (10)	1.0	
	3.0+	57 (10)	4.12 (1.4–11.8)	0.008
Ferritin, ng/mL	---	502 (20)	1.80 (1.0–3.1)	0.038
Aspartate aminotransferase, U/L	---	502 (20)	1.85 (1.0–3.5	0.062
Creatinine, mg/dL and BNP, pg/mL^2^	0	339 (8)	1.0	
(# of risk factors)	1	104 (2)	0.53 (0.1–2.5)	0.43
	2	59 (10)	2.73 (0.9–8.4)	0.079

1 HR is presented for 75^th^ relative to the 25^th^ percentile, calculated as e^coefficient×(75th percentile–25th percentile)^, for each variable listed in the table, unless otherwise indicated. Values shown are adjusted for all other variables in the model.

2 HR is given for the combined influence of creatinine and BNP on mortality as defined by the creatinine levels >0.9 or BNP levels ≥160, or both, relative to creatinine ≤0.9 and BNP<160.

The results from an analysis of TRV as a composite variable combined with NT-proBNP are presented in **Table 4**. For patients with both high TRV (≥3.0 m/sec) and high NT-proBNP (≥160 pg/mL), the unadjusted hazard ratio was 14.86 (95% CI 5.5–39.9; p<0.0001) and 11.10 (95% CI 4.0–30.8; p<0.0001) after adjustment for ferritin. Adjustment for AST and creatinine had little effect on the significance of the association between the combined TRV and NT-proBNP variable and mortality and were not included in the model.

**Table 4:** Cox Proportional Hazards Regression Analysis of Mortality for Composite of TRV and BNP.

Risk Factor	Category	N at Risk (deaths)	Hazard Ratio (95% CI)^1^	p
Unadjusted TRV/BNP Composite^1^	0	381 (7)	1.0	
(# of risk factors)	1	105 (3)	1.56 (0.4–6.0)	0.52
	2	39 (9)	14.86 (5.5–39.9)	<0.001
Adjusted TRV/BNP Composite^1^^,^ 2	0	366 (7)	1.0	
(# of risk factors)	1	96 (3)	1.44 (0.4–5.6)	0.60
	2	36 (9)	11.10 (4.0–30.8)	<0.001
Ferritin, ng/mL^3^	---		2.15 (1.2–3.8)	0.008

1 HR is given for the combined influence of TRV and BNP on mortality as defined by TRV levels ≥3.0 or BNP levels ≥160, or both, relative to TRV<3.0 and BNP<160.

2 Adjusted for ferritin.

3 HR presented for 75^th^ relative to the 25^th^ percentile, calculated as e^coefficient×(75th percentile–25th percentile)^, and adjusted for TRV/BNP composite.

## Discussion

Here we show that amongst a large multinational cohort of patients with SCD, a TRV≥3.0 m/sec is common and has the highest risk for death of any measured variable. This risk is higher when an elevated TRV is combined with an elevated NT-proBNP level confirming the value of these measurements as risk stratification screening tools in patients with SCD.

An estimate based on a Doppler-echocardiographic measured TRV value ≥3.0 m/sec suggests that 11.2% of the SCD population screened are at high risk of having PH. This result is consistent with the recently published analysis of the NIH-Pulmonary Hypertension Screening Study, which reported 84 right heart catheterizations in 531 SCD subjects and a PH prevalence of 10.4% [8]. It is also consistent with a PH prevalence of 10% reported by Fonseca et al. in the evaluation of a smaller Brazilian cohort [7]. These numbers are higher than the 6% prevalence observed by Parent and colleagues, however they excluded 10% of their patient population from screening, those with elevated international normalized prothrombin time ratio >1.7, estimated creatinine clearance <30 mL per minute and forced expiratory vital capacity <70% predicted [6]. Because hepatic dysfunction, renal insufficiency and low total lung capacity are associated with PH in the sickle cell disease population [3], [17], they likely excluded a group with a much higher prevalence of PH. This would be expected to reduce prevalence estimates.

This study identified a number of risk factors for early death previously described in the Cooperative Study of Sickle Cell Disease (CSSCD) such as age and male gender [18]. It also confirmed risks associated with elevated TRV, elevated NT-proBNP, increasing creatinine, intensity of hemolytic anemia, and ferritin. Additionally, the New York Heart Functional Classification and number of red blood cell units transfused were also found to be associated with higher risk of death in univariate analysis. In multivariate analysis TRV, ferrritin, AST, and creatinine or NT-pro-BNP remained independent predictors of mortality. Of all the measured parameters the TRV carried the highest hazards ratio for early death, associated with a 10-fold increased risk. This was even more significant when combined with a high NT-proBNP (14-fold increased risk of death), potentially reflecting PH with right heart failure.

There are a number of major limitations to this study. Most patients with an elevated TRV did not have a right heart catheterization to confirm the diagnosis of PH. For these estimates we refer to the recent RHC studies and published operating characteristics for this test at this threshold value. The causes of death are largely unknown as there were no autopsies available and the trial was not funded to establish the definitive cause of death for all patients. In the recent analysis of the NIH-PH cohort, death certificates were available for 15 out of 23 (65%) subjects with PH and 80% of these subjects were reported to have had right heart failure or sudden cardiac death stated as a cause of death [8]. In addition, we cannot estimate the percentage of patients with an elevated TRV that had pulmonary arterial hypertension (PAH) or pulmonary venous (or post-capillary) hypertension (PVH). According to the NIH cohort [8] study 5.8% of the entire cohort had PAH and in the Fonseca study 3.75% had PAH [7]; based on these data it is likely that half of the patients with PH would have PAH and half PVH. It is notable in the Mehari study that even patients diagnosed with PVH had elevated transpulmonary gradients and the pulmonary vascular resistance and transpulmonary gradients predicted risk of death, while the pulmonary capillary wedge pressure did not [8], [19]. These data suggest that pulmonary vascular disease is central to the mechanism of disease and death observed in the current study.

Recent editorials have suggested that PH is rare in patients with sickle cell disease and not a cause of death “per se” in this patient population [9], [10]. To our knowledge, scleroderma, with a prevalence of 7–11% and portopulmonary hypertension (1–6% prevalence) are the only diseases with a prevalence of PAH that is comparable to sickle cell disease. Based on this accepted high prevalence, all patients with scleroderma and patients with portal hypertension being evaluated for liver transplantation are screened for the development of PH and referred to specialty care [20]. Similar to certain diseases associated with PAH, there have been no completed trials in patients with sickle cell disease with sufficient power to evaluate the efficacy of PAH targeted therapy. However, there are a number of interventions that would be expected to reduce morbidity and mortality in this population, including more aggressive hydroxyurea or transfusion therapy, iron chelation, supplemental oxygen and identification and treatment of thromboembolic disease and sleep disordered breathing. For these reasons, we suggest that screening for PH associated with sickle cell disease is helpful and would have a positive impact on the well-being of these patients.

## Supporting Information

Checklist S1 Supporting CONSORT checklist.
(PDF)

Protocol S1 (PDF)
