# Supplementary material for: Risk Factors for Death in 632 Patients with Sickle Cell Disease in the United States and United Kingdom
Source: PLoS One. 2014 Jul 2;9(7):e99489. doi: 10.1371/journal.pone.0099489 (PMC4079316; doi:10.1371/journal.pone.0099489)
Supplement: Protocol S1 — (PDF) [file pone.0099489.s002.pdf]

walk PHaSST

CLINICAL PROTOCOL

PROTOCOL TITLE: Treatment of Pulmonary Hypertension and Sickle Cell Disease with Sildenafil Therapy

PROTOCOL NAME: walk-PHaSST

IDENTIFYING WORDS: PDE inhibitors, sickle cell, pulmonary hypertension

DATE: 20 January 2009

VERSION NUMBER: 7.0

PRIMARY AUTHORS: Roberto Machado, MD
Mark Gladwin, MD
National Heart Lung and Blood Institute (Intramural)

PROTOCOL COMMITTEE: Kathryn Hassell, MD
Petra LeBeau, ScD
Roberto Machado, MD
Nancy Yovetich, PhD

SPONSOR: National Heart Lung and Blood Institute
Project Officer: Jonathan Goldsmith, MD

DATA COORDINATING CENTER: Rho Federal Systems Division, Inc.
6330 Quadrangle Drive, Suite 500
Chapel Hill, NC 27517
Phone: 919-408-8000
Fax: 919-408-0999

STUDY SITES: Children's Hospital, Oakland
Children's Hospital, Pittsburgh
Albert Einstein College of Medicine/Columbia University Medical Center, New York
Howard University, Washington D.C.
Imperial College London and Hammersmith Hospital, London
Johns Hopkins University, Baltimore
NHLBI (intramural), Bethesda
University of Colorado, Denver
University of Illinois, Chicago

SYNOPSIS

Protocol Title: Treatment of Pulmonary Hypertension and Sickle Cell Disease with Sildenafil Therapy

IND Number: 77461

IND Holder: National Heart Lung and Blood Institute (NHLBI)

Responsible Party: Jonathan Goldsmith, MD

Study Site(s):

Children's Hospital, Oakland
Children's Hospital, Pittsburgh
Albert Einstein College of Medicine/Columbia University Medical Center, New York
Howard University, Washington D.C.
Imperial College London and Hammersmith Hospital, London
Johns Hopkins University, Baltimore
NHLBI (intramural), Bethesda
University of Colorado, Denver
University of Illinois, Chicago

Phase of Development: Post-marketing, Phase II/III

Objectives:

Primary Objective(s):

In a randomized, double-blind, placebo-controlled phase II/III trial, to determine the efficacy of 16 weeks of sildenafil therapy on exercise capacity (six-minute walk distance).

Secondary Objectives:

1. To determine the efficacy of 16 weeks of sildenafil therapy on echocardiographic estimates of right ventricular systolic pressure, and symptoms in patients with sickle cell disease and pulmonary hypertension, defined by tricuspid regurgitant velocity (TRV) ≥ 2.7 m/sec
2. Via a right heart catheterization in subjects with more severe pulmonary hypertension (TRV ≥ 3.0 m/sec stratum), to evaluate and compare the acute hemodynamic effects of inhaled nitric oxide and of oral sildenafil at rest in the catheterization laboratory and to determine the changes in hemodynamics after 16 weeks of sildenafil therapy
3. To determine the safety of 16 weeks of sildenafil therapy via adverse event reports and laboratory assessments
4. To evaluate prospective clinical outcomes in the subjects participating in the Observational Follow-up Study to the extent possible by each participating site
5. To provide subjects with open-label sildenafil for up to one year after completion of the Main Interventional Trial and to evaluate the long term safety of sildenafil in this population

Statement of Primary Efficacy Hypothesis and Primary Analysis:

The primary efficacy hypothesis is that 16 weeks of sildenafil therapy provides greater improvement in six-minute walk (6MW) distance than does placebo in patients with sickle cell disease (SCD) and pulmonary hypertension.

The primary efficacy analysis is an Analysis of Covariance Analysis (ANCOVA) on 6MW distance change from baseline to Week 16 on the Intent to Treat (ITT) Population. The primary hypothesis test will be based on a test that the average change differs between the two treatment groups, with baseline 6MW distance and TRV stratum used as covariates. This type of model controls for any impact of baseline 6MW on the treatment effect without assumptions about the slope of the relationship between the baseline and Week 16 measures.

Planned Sample Size: Up to 1000 subjects will be screened. A total of 132 subjects will be randomized into the Main Interventional Trial. Recruitment is expected to extend for approximately 13 months.

Study Design: Up to 1000 patients will be screened based on medical history, physical examination, laboratory testing, transthoracic Doppler-echocardiography, and 6MW test. All consented subjects in this

cohort will provide plasma and serum for a biomarker and DNA repository and will be followed prospectively in the Observational Follow-up Study.<br>Subjects found to have a screening TRV of $\geq$ 2.7 m/s will be invited to participate in the Main Interventional Trial. Upon repeat echocardiogram at baseline, subjects with TRV $\geq$ 2.7 m/s who meet all inclusion/exclusion criteria will be enrolled in the 16 week, randomized, double-blind, placebo-controlled trial of sildenafil versus placebo. The study will randomize 132 subjects in the double-blind phase of the MIT. Subjects will be stratified and randomized based on site and TRV. The primary endpoint is the change in 6MW distance across this study phase. Secondary endpoints include non-invasive estimation of pulmonary artery systolic pressure by Doppler-echocardiography, plasma NT-BNP levels, cardiovascular and sickle cell related symptoms and events, and quality of life scores. Study assessments will occur at weeks 6, 10, and 16 or early withdrawal. One-half of the subjects, those with TRVs ≥ 3.0 m/s, will also be evaluated with right heart catheterization (RHC) before and after 16 weeks of the intervention.<br>Subjects who complete the double-blind phase will be eligible to participate in the Open-label Follow-up Phase and will be treated with sildenafil for up to 1 additional year. Adverse event data will be collected every 1-3 months during this open-label phase.
**Diagnosis and Key Patient Selection Criteria:**<br>**Screening Phase**<br>Males or females 12 years of age or older with a diagnosis of sickle cell disease (documentation of sickle cell disease, including but not limited to, SS, SC, SD, or Sβ°/+ thalassemia phenotype is required).<br>**Double-blind Phase of Main Interventional Trial**<br>Qualification for screening phase, electrophoretic documentation of sickle cell disease, at least mild pulmonary hypertension with TRV ≥ 2.7 m/s by echocardiogram, and 6MW distance of 150-500 m.<br>**Open-label Follow-up Phase of Main Interventional Trial**<br>Completion of Main Interventional Trial.<br>**Observational Follow-up Study**<br>Qualification for screening phase, provision of informed consent, and a) disqualification for Main Interventional Trial; b)discontinuation from Main Interventional Trial/Open-label phase; or c) completion of Main Interventional Trial/Open-label phase.
**Treatments:** Sildenafil (20, 40, 80 mg) or matching placebo during the double-blind phase of the Main Interventional Trial. Sildenafil (doses range: 20-80 mg) during Open-label Follow-up Phase.

LIST OF ABBREVIATIONS

6MW	6 Minute Walk
AE	adverse event
ANCOVA	analysis of covariance
ACS	acute chest syndrome
aPTT	activated partial thromboplastin time
BPI	Brief Pain Inventory
cGMP	cyclic guanosine monophosphate
CI	Clinical Investigator
CRA	Clinical Research Associate
CRF	case report form
DSMB	Data Safety Monitoring Board
DCC	Data Coordinating Center
EOP	end of phase
EOS	end of study
INR	international normalize ratio
ITT	Intent-to-Treat
LDH	Lactate dehydrogenase
LOCF	last observation carried forward
LVF	left ventricular function
MPAP	mean pulmonary arterial pressure
MIT	Main Interventional Trial
NHLBI	National Heart, Lung, and Blood Institute
NIH	National Institutes of Health

NO	nitric oxide
NYHA/WHO	New York Heart Association/World Health Organization
OFS	Observational Follow-up Study
PAP	pulmonary arterial pressure
PAH	pulmonary arterial hypertension
PDE5	phosphodiesterase Type 5
PH	pulmonary hypertension
PO	per os (by mouth, orally)
ppm	parts per million
PVR	pulmonary vascular resistance
RVF	right ventricular function
RVSp	right ventricular systolic pressure
SAE	serious adverse event
S$\beta^{+/o}$thalassemia	sickle cell disease genotype
SC	sickle cell disease genotype
SCD	sickle cell disease
SD	sickle cell disease genotype
SS	sickle cell disease genotype
SVR	systemic vascular resistance
TRV	tricuspid regurgitant velocity
VOC	vaso-occlusive crisis

TABLE OF CONTENTS

SYNOPSIS **2**
LIST OF ABBREVIATIONS **4**
TABLE OF CONTENTS **6**
LIST OF TABLES **9**
LIST OF FIGURES **9**
1 PRECIS **10**
2 INTRODUCTION **11**
2.1 Sickle Cell Disease and Pulmonary Hypertension 11
2.2 Decreased NO Bioavailability in Hemolytic Disorders Provide Rationale for NO Based Therapies of Pulmonary Hypertension 12
2.3 Sildenafil Therapy in Hemolysis Associated Pulmonary Hypertension 13
2.4 Genetic Epidemiology and Candidate Gene Modifiers in Sickle Cell Hemolysis Associated Pulmonary Hypertension 17
3 STUDY OBJECTIVES AND PURPOSE **17**
4 STUDY DESIGN AND METHODS **18**
4.1 Overview 18
4.2 Screening Phase 20
4.3 Observational Follow-up Study 20
4.4 Main Interventional Trial 21
4.4.1 Description of Study Endpoints 21
4.4.2 Description of Study 22
4.4.2.1 Baseline 22
4.4.2.2 Right Heart Catheterization (RHC) Procedure 22
4.4.2.3 Monitoring for Priapism 23
4.4.2.4 Randomized, Double-blind Phase 24
4.4.3 Alternative Therapies during the Double-blind Phase 26
4.4.4 Open-Label Follow-up Phase 27
5 SELECTION OF SUBJECTS **28**
5.1 Screening Phase 28
5.1.1 Inclusion Criteria 28
5.2 Observational Follow-up Study 28
5.2.1 Inclusion Criteria 28
5.3 Main Interventional Trial 28
5.3.1 Inclusion Criteria 28
5.3.2 Exclusion Criteria 29
5.3.3 Randomization Criteria 30
6 RANDOMIZATION AND BLINDING **30**
7 MONITORING OF SUBJECTS AND CRITERIA FOR WITHDRAWAL OF SUBJECTS **31**
7.1 Subject Discontinuation 31
7.2 Dose Modification or Discontinuation Due to Expected Adverse Events: Headache or Facial/skin Edema 32
8 DESCRIPTION OF STUDY DRUG AND DOSING REGIMEN **33**
8.1 Packaging, Labeling, Blinding, Storage, and Return of Study Drug 33
8.2 Subject Compliance 34

9 CLINICAL AND LABORATORY ASSESSMENTS 34
9.1 Schedule of Study Events 34
9.2 Efficacy Evaluations 37
9.2.1 Echocardiography 37
9.2.2 Right Heart Catheterization 37
9.2.3 6-Minute Walk 38
9.2.4 Clinical Outcome Adjudication 38
9.2.5 Candidate Gene Analysis 39
9.3 Scientific and Safety Evaluations 39
9.3.1 Biomarker Analyses 39
9.3.2 Clinical Laboratory Tests 39
9.3.3 Physical Examination and Vital Signs 40
9.3.4 Medical History 40
10 DATA MANAGEMENT 41
10.1 Source Documentation 41
10.2 Data Management 41
10.3 Staff Training 41
10.4 Data Monitoring 41
11 STATISTICAL METHODS 42
11.1 Analysis Populations 42
11.2 Efficacy Analyses 42
11.2.1 Primary Efficacy Endpoint 42
11.2.2 Secondary Efficacy Endpoints 43
11.3 Safety Analyses 45
11.4 Interim Analyses 45
11.5 Statistical Considerations 45
11.5.1 Covariates 45
11.5.2 Multi-center Studies 46
11.5.3 Multiple Comparisons and Multiplicity 46
11.5.4 Examination of Subgroups 46
11.5.5 Handling of Dropouts and Missing Data 46
11.6 Sample Size 47
11.7 Statistical Estimates for Genetic Analysis 49
12 HUMAN SUBJECT PROTECTION 50
12.1 Rationale for Subject Selection 50
12.2 Participation of Children 51
12.2.1.1 Sickle Cell Disease and Pediatric PH 51
12.3 Evaluation of Benefits and Risks/Discomforts 52
12.3.1 Risks/Discomforts of Procedures 52
12.3.1.1 Arterial and Venous Angiocatheters 52
12.3.1.2 Central Venous Catheter (CVC) Introducer 52
12.3.1.3 Pulmonary Artery Catheter 52
12.3.1.4 Fluoroscopy 53
12.3.2 Risks/Discomforts of Study Medications 53
12.3.2.1 Sildenafil Use in Healthy Volunteers 53
12.3.2.2 Adverse Reactions: Pre-marketing Experience 54

12.3.2.3 Adverse Reactions: Post-marketing Experience 55
12.3.2.4 Sildenafil and Sickle Cell Disease 58
12.3.3 Benefits of Sildenafil Therapy 59
12.4 Protocol Consent Processes and Documents 59
12.5 Patient Advocate 59
13 SAFETY EVALUATIONS AND REPORTING PLAN 59
13.1 Safety Assessments Overview...59
13.2 Definitions 60
13.2.1 Adverse Events 60
13.2.2 Serious Adverse Events 60
13.2.3 Adverse Clinical Laboratory Trend 60
13.3 Assessment of Adverse Event Severity and Relationship to Treatment.........61
13.4 Assessment of Adverse Event Outcome and Action(s) Taken.......................62
13.5 Reporting of Adverse Events..63
13.6 Reporting of Serious Adverse Events..63
13.6.1 Initial Reporting of SAEs by the Clinical Investigator (CI) 63
13.6.2 IND Safety Reporting to the FDA 64
13.6.3 Safety Reporting in the United Kingdom 66
13.7 Reporting of Adverse Events to the IRB 66
13.7.1 Expected AEs that Require no IRB Reporting 66
13.7.2 SAEs that Require Expedited Reporting to the IRB 68
13.8 Reporting of Serious Safety Issues to the DSMB; Suspension Guidelines 69
13.8.1 Reporting of All Serious Adverse Events to the DSMB........................... 70
13.8.2 Reporting of All Adverse Events (Serious and Non-serious).................. 70
13.8.3 Reporting of Adverse Clinical Laboratory Trends 71
13.9 Pregnancy Reporting 72
13.10 Clinical and Safety Monitoring 73
13.10.1 Subject Safety Monitoring 73
13.10.2 Clinical Monitoring Plan.. 73
13.10.3 Plan for Reporting Protocol Violations/Deviations 73
14 COMPENSATION 73
15 STUDY CONDUCT..73
16 INVESTIGATOR AGREEMENT 75
17 REFERENCES 76
18 APPENDICES..82
APPENDIX A: ECHOCARDIOGRAPHIC PARAMETERS....................................83
APPENDIX B: PATIENT HISTORY INTAKE DOCUMENT 84
APPENDIX C: ACUTE INHALED NO AND SILDENAFIL CHALLENGE AT BASELINE AND END OF MIT 87
APPENDIX E: CANDIDATE GENES 91

List of Tables

Table 1. Summary of Relevant Literature 15
Table 2. Inhaled NO and Oral Sildenafil Challenge at Baseline 23
Table 3. Schedule of Events – Screening and Observational Follow-up Study 35
Table 4. Schedule of Events – Main Interventional Study 36
Table 5. Investigational Laboratory Studies (Biomarkers) 39
Table 6. Sample size needed per treatment arm by assumed standard deviation and power 47
Table 7. Relative Risk Ratio 50
Table 8. Adverse Events Reported more Frequently by Patients Treated with Sildenafil than those Treated with Placebo in PRN Flexible-Dose Phase II/III Studies .. 55
Table 9. Sildenafil Trials in Men and Women with Pulmonary Hypertension with Published Adverse Events 58
Table 10. Relationship between Treatment and AE 62
Table 11. List of Sickle Cell Related or Right Heart Catheterization Related Adverse Events 65
Table 12. Summary of Procedures and Timing for Alerting the DSMB and NHLBI Project Officer of Possible Serious Safety Issues 72
Table 13. Modified NYHA Functional Classification 89
Table 14. Borg dyspnea score 90
Table 15. Candidate Genes in Cell Adhesion Molecules 91
Table 16. Candidate Genes - Cytokine and Regulatory Molecules 91
Table 17. Candidate Genes – Major Human Erythrocyte Membrane Proteins 92
Table 18. Candidate Genes Identified By Gene Expression Analysis in SCD 93

List of Figures

Figure 1. Effects of sildenafil on (A) pulmonary pressures and (B) functional capacity 16
Figure 2. walk-PHaSST Study Design 19

1 PRECIS

Sickle cell disease (SCD) is an autosomal recessive disorder and the most common genetic disease affecting African-Americans. Approximately 0.15% of African-Americans are homozygous for sickle cell disease, and 8% have sickle cell trait. Acute pain crisis, acute chest syndrome (ACS), and pulmonary hypertension are common complications of sickle cell anemia. Pulmonary hypertension (PH) has now been identified as a marker of mortality in adults with sickle cell disease. Sildenafil has been proven beneficial in pulmonary hypertension (PH) and recent phase I/II studies from the intramural National Institutes of Health (NIH) suggest it is well tolerated and efficacious in the SCD population. Furthermore, a number of recent studies have suggested that nitric oxide (NO) based therapies may have a favorable impact on sickle red cells at the molecular level and could improve the abnormal microvascular perfusion that is characteristic of sickle cell anemia.

The project has 3 distinct components:

1. **Screening Phase**. Approximately 1000 subjects with sickle cell disease will be screened. Assessments will include historical and laboratory data, Doppler echocardiogram, 6-minute walk test, plasma/serum, and DNA for banking.

2. **Main Interventional Trial**. The randomized, double-blind, placebo controlled phase is designed to determine the effects of 16 weeks of sildenafil therapy on exercise endurance, cardiopulmonary hemodynamic parameters and symptoms in this patient population. The Open-label Follow-up Phase is designed to provide up to 1 additional year of sildenafil therapy to subjects who completed the randomized, double-blind phase.

3. **Observational Follow-up Study.** All patients who sign informed consent for the Screening Phase are also consenting to participate in the Observational Follow-up Study. Screened subjects will proceed into the Observational Follow-up Study if a) they do not qualify for -or choose not to participate in- the MIT; b) are discontinued for any reason during the MIT or the Open-label Follow-up Phase; or c) complete the Open-label Follow-up Phase. Subjects may be contacted every 6-12 months for up to 3 years to assess major disease-related complications, including mortality; subject follow-up during this study will be at the discretion of each clinical site.

2 INTRODUCTION

2.1 Sickle Cell Disease and Pulmonary Hypertension

Sickle cell disease (SCD) occurs in individuals who are homozygous for a single nucleotide substitution in the β-globin gene that ultimately renders their hemoglobin (HbS) much less soluble than normal hemoglobin (HbA) when deoxygenated. This insolubility causes aggregation (or polymerization) of HbS inside sickle erythrocytes as they traverse the microcirculation. The severity of sickle cell anemia has been described by models that emphasize either the intracellular kinetics of polymer formation and cell sickling or the extent of polymerization and cell rigidity at the reduced oxygen saturation values of various tissues and organs. The pathogenesis of sickle cell anemia is also thought to be affected by adhesion of the sickle erythrocytes to the microvascular endothelium. Increased expression of adhesion molecules on erythrocytes (α4β1, CD36) and endothelial cells (VCAM-1, CD36), interaction with leukocytes, increased levels of circulating inflammatory cytokines, enhanced microvascular thrombosis, and endothelial damage are all thought to contribute to obstruction of the arterioles by polymer-containing sickle erythrocytes. Factors that increase the intracellular concentration of hemoglobin (red blood cell dehydration), increase time spent in the microcirculation (increased expression of adhesion molecules endothelial VCAM-1 and erythrocyte α4β1), or increase deoxygenation of hemoglobin, all contribute to increased HbS polymerization. Other sickle cell syndromes, include sickle-hemoglobin C (HbSC), sickle-hemoglobin D (HbSD), and sickle-$\beta^{o/+}$thalassemia (HbSβthal), vary in severity as compared to sickle cell anemia (HbSS), but sickle hemoglobin polymerization, chronic vascular injury and organ damage can occur.

Pulmonary hypertension, a disorder characterized by an elevated pulmonary artery pressure (PAP) and pulmonary vascular resistance (PVR), is an increasingly recognized complication of sickle cell anemia[1-7]. Pulmonary hypertension has been defined by the 1981 NHLBI/NIH national registry as a mean pulmonary artery pressure (MPAP) ≥ 25 mm Hg at rest or ≥ 30 mm Hg with exercise [8]. Echocardiographic studies performed at tertiary care sickle cell centers have reported that 10-30% of screened patients have pulmonary hypertension (MPAP ≥ 25 mm Hg) [1, 2]. Furthermore, sickle cell patients with pulmonary hypertension have significantly increased mortality rate compared with patients without pulmonary hypertension. Sutton and colleagues reported a 40% mortality rate at 22 months with an odds ratio for death of 7.86 (2.63-23.4)[2]. Powars and colleagues reported a mean 2.5 year survival in sickle cell patients with chronic lung disease with pulmonary hypertension [9]. Data presented at the 1999 American Thoracic Society by investigators at Wayne State University revealed a 30% prevalence of pulmonary hypertension in their sickle cell clinic population and a 30% two-month mortality rate. Castro and colleagues [10] reported a 50% two year mortality rate in patients with sickle cell disease with pulmonary hypertension confirmed by right heart catheterization (RHC). It is clear that this disease, similar to idiopathic PAH (formerly primary pulmonary hypertension) and pulmonary hypertension associated with other conditions, e.g. scleroderma and HIV, carries an unacceptably high morbidity and mortality rate. The variability in 6 to12 month prognoses is likely attributable to the variability in the PH in the patients studied.

These data are consistent with the results of an NIH pulmonary hypertension screening study [11]. 195 adult patients with sickle cell disease were recruited from the greater Washington, DC region. All patients were screened with transthoracic echocardiograms and the tricuspid regurgitant jet velocity (TRV) was used to estimate the pulmonary artery systolic pressure. Pulmonary hypertension was prospectively defined by a TRV ≥ 2.5 m/s and moderate-to-severe PH defined by a TRV ≥ 3.0 m/s. Right heart catheterization was performed in consenting patients with TRV ≥ 2.8 m/s. This study revealed that 32% of patients with SCD had elevated pulmonary artery systolic pressure (TRV ≥ 2.5 m/s) and 9% had at least moderately elevated pressures (TRV ≥ 3.0 m/s). Multiple logistic regression analysis identified a history of renal or cardiovascular complications, increased systemic systolic blood pressure, markers of hemolysis (LDH), elevated alkaline phosphatase, and low transferrin levels as independent predictors of PH. Most importantly, TRV of at least 2.5 m/s, as compared to a velocity < 2.5 m/s, was associated with an increased risk of death (RR 10.1; 95% CI, 22. 47; P < 0.001) and remained so after adjustment for other possible risk-factors in proportional hazards regression analysis. The 18 month mortality approached 20% for patients with a TRV ≥ 2.5 m/s. In another recent prospective study of 60 patients systematically sampled at a comprehensive sickle cell treatment center, the prevalence of PH (defined by an age and body-mass index adjusted nomogram) was 30 % [12]. Further updated follow-up data from our cohort continues to demonstrate that PH is a strong independent risk factor for mortality with 45-month mortality rate of approximately 40 %. From Cox regression analysis the estimated risk ratio of death, relative to patients with TRV < 2.5 m/s, was 4.4 (95% CI 1.6 to 12.2; P < 0.001) for patients with TRV 2.5 to < 3.0 m/s and 10.6 (95% CI 3.3 to 33.6; P < 0.001) for patients with TRV > 3.0 m/s. In addition, a study by De Castro and colleagues presented at the meeting of the American Society of Hematology reported a similar prevalence of PH and a remarkably similar 17% mortality rate for patients with PH over 2 years compared with approximately 2% for subjects without pulmonary hypertension [13]. Ataga and colleagues have also shown a 10% mortality rate for patients with PH over 26 months in comparison to 1% in those patients without PH[12]. Taken together, the retrospective and prospective studies strongly support the contention that PH is the greatest risk factor facing the aging population of patients with sickle cell disease and likely patients with other chronic hemolytic disorders.

Clearly, therapeutic trials targeting pulmonary hypertension in this high risk population are indicated.

2.2 Decreased NO Bioavailability in Hemolytic Disorders Provide Rationale for NO Based Therapies of Pulmonary Hypertension

As a result of hemolysis, hemoglobin is released into plasma where it reacts with and destroys nitric oxide (NO), resulting in abnormally high NO consumption. Consequently, smooth muscle guanylyl cyclase is not activated and vasodilation is inhibited. Plasma from patients with SCD contains cell-free ferrous hemoglobin, which stoichiometrically consumes micromolar quantities of NO and abrogates forearm blood flow responses to NO donor infusions, and that hemoglobin oxidation by NO inhalation restores NO bioavailability[14, 15]. As such, plasma hemoglobin and oxygen free radical-mediated consumption of NO produces a state of resistance to NO in patients with sickle cell

disease. NO destruction by hemoglobin can also cause further impairment in vascular endothelial function via transcription derepression of adhesion molecules, including VCAM-1 and E-selectin, and vasoconstrictor/growth factors such as endothelin-1[16, 17]. In addition to hemoglobin decompartmentalization, hemolysis releases erythrocyte arginase, which converts L-arginine, the substrate for NO synthesis, to ornithine [18-22]. Consistent with this observation, in patients with sickle cell disease, the arginine-to-ornithine ratio decreases significantly as pulmonary pressures increase and the patients with low ratios have the highest prospective mortality [11, 20].

These alterations in NO bioavailability are likely to be involved in the pathogenesis of the secondary PH associated with SCD and other chronic hemolytic disorders. As such, therapeutic interventions that enhance NO effects or act as NO donors are of potential benefit and may alter the progression of the disorder. The sub-population of patients with SCD who have PH suffers the highest morbidity and mortality and is most likely to respond to these therapies.

Elevation in lactate dehydrogenase (LDH) is significantly associated with evidence of chronic hemolysis, including low levels of hemoglobin and haptoglobin, high levels of reticulocytes, bilirubin, plasma hemoglobin, and aspartate aminotransferase (AST). Elevated LDH levels also closely correlates with accelerated NO consumption by plasma, impaired vasodilatory response to NO donors, and is identified in patients with a syndrome of hemolysis-associated NO resistance, endothelial dysfunction and end-organ vasculopathy[23]. This association has been confirmed in a longitudinal study of pulmonary hypertension in sickle cell disease patients[12]. It has been suggested that LDH may be a simple but important biomarker for the clinical phenotype of pulmonary hypertension induced by chronic hemolysis[14].

2.3 Sildenafil Therapy in Hemolysis Associated Pulmonary Hypertension

Endothelial NO activates the enzyme guanylate cyclase, which results in increased levels of cyclic guanosine monophosphate (cGMP), producing smooth muscle relaxation. Sildenafil has no direct relaxant effect, but enhances the effect of NO by inhibiting phosphodiesterase type 5 (PDE5), which is responsible for degradation of cGMP. Studies *in vitro* have shown that sildenafil is selective for PDE5. Its effect is more potent on PDE5 than on other known phosphodiesterases (>80-fold for PDE1, >1,000-fold for PDE2, PDE3, and PDE4).

In addition to human corpus cavernosum smooth muscle, PDE5 is also found in lower concentrations in other tissues including platelets, vascular and visceral smooth muscle, and skeletal muscle. The inhibition of PDE5 in these tissues by sildenafil may be the basis for the enhanced platelet anti-aggregatory activity of NO observed *in vitro*, an inhibition of platelet thrombus formation *in vivo* and peripheral arterial-venous dilatation *in vivo*. Recent studies now suggest that PDE5 is also present in greater abundance in the pulmonary vasculature. Sildenafil has thus been found to promote relatively selective pulmonary vasodilation in different subgroups of patients with pulmonary hypertension and is being utilized as an alternative therapeutic agent[24-32].

Acute administration of a single oral dose of sildenafil (50-100 mg) causes a significant decrease in MPAP, PVR with minimal or no effects on mean systemic arterial pressure. The effect peaks at 60 minutes and lasts as long as 4 hours[27-31]. The magnitude of the effect in the pulmonary circulation is comparable to that of inhaled NO, iloprost and epoprostenol [29, 30].

Case series have also documented a favorable effect of chronic sildenafil administration in patients with PH. Ghofrani et al [26] treated 12 patients with inoperable chronic thromboembolic disease and PH. After 6 months of sildenafil therapy (50 mg three times/day) MPAP decreased from 53 to 45 mmHg (P = 0.03) and 6MW distance increased from 312 to 366 m (P = 0.02). Three months of sildenafil therapy added to standard of care was also associated with improvements in functional class, walking distance, PAP and right ventricular mass in five individuals with PH [32]. In another group of 14 patients with PH failing iloprost therapy, the addition of sildenafil for up to 12 months was also associated with improvements in pulmonary hemodynamics and exercise capacity [25]. The cumulative published experience from case series involving more than two patients is summarized in Table 1. A recently completed phase III study of sildenafil for PH confirmed these observations [24]. In this trial 277 patients were randomized to receive placebo or sildenafil 20mg, 40mg or 80mg three times daily. All three sildenafil treatment groups demonstrated a significant improvement in exercise capacity when compared to placebo with placebo corrected effects on 6MW distance ranging from 45 to 50 m, but without a dose related response. Sildenafil treatment significantly decreased MPAP and PVR and increased cardiac output and mixed venous oxygen saturation; these hemodynamic responses were maximal at 80 mg three times a day. None of the men enrolled in these trials experienced priapism or unwanted erections.

Over the last four years we have treated 22 patients with SCD and PH with sildenafil under protocols 03-CC-0127 and 01-H-0223, for a combined total exposure time of 44 patient years. These experiences suggest that sildenafil is well tolerated in this population (see section 12.3.2) and effectively lowers PAPs and increases exercise capacity (Figure 1). None of the male patients experienced severe sustained priapism. While the observed effects are comparable to inhaled NO gas, the oral modality of sildenafil therapy offers substantial advantages. Based on these data, we hypothesize that chronic sildenafil therapy in patients with PH associated with SCD will result in improvements in exercise capacity and pulmonary hemodynamics.

Table 1. Summary of Relevant Literature

Reference	Number of Patients	Dose	Duration	Main Results
Lepore [30]	9	50 mg X1	Single dose	Significant decrease in MPAP
Michelakis [31]	13	75 mg X1	Single dose	Significant 27% decrease in MPAP
Ghofrani [28]	8	50 mg	Single dose	Significant 32.5 % decrease in PVR index
Michelakis [32]	5	50 mg TID	3 months	Significant improvement in NYHA functional class, 6MW distance, MPAP and PVR
Ghofrani [25]	14	25 mg TID- 50 mg TID	9-12 months	Significant improvement in NYHA functional class, 6MW distance, MPAP and PVR
Ghofrani [26]	12	50 mg TID	6 months	Significant improvement in 6MW distance, MPAP and PVR
Galie [24]	278	20mg, 40 mg or 60 mg TID	12 weeks	Significant improvement in MPAP, oxygen saturation and exercise capacity

Figure 1. Effects of sildenafil on (A) pulmonary pressures and (B) functional capacity

Subtitle: As measured by six minute walk distance, in patients with pulmonary hypertension and sickle cell disease

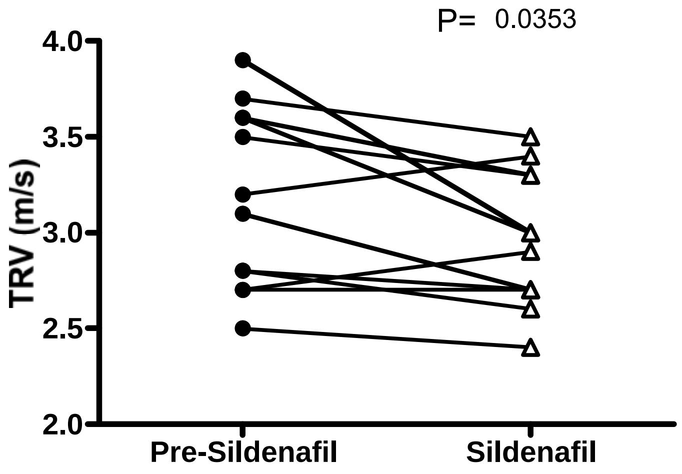

A)

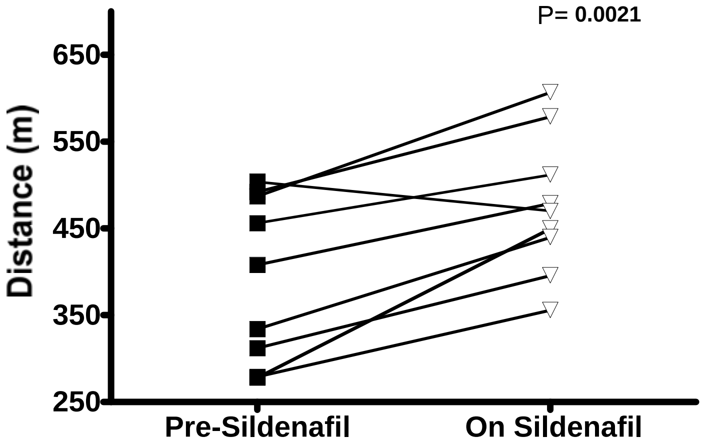

B)

This protocol sponsored by the
National Heart Lung and Blood Institute

2.4 Genetic Epidemiology and Candidate Gene Modifiers in Sickle Cell Hemolysis Associated Pulmonary Hypertension

Clinical risk factors only account for 40% (R^2=0.40) of the variability associated with the development of PH, therefore genetic background risk factors for the development of PH in SCD are likely to play a role. The current study will be one of the largest cohorts of SCD patients where cardiovascular phenotype is extensively characterized. The β^S mutation occurs on at least five different haplotypes defined by the presence of restriction sites within the 50 kilobase β-globin gene cluster which includes a region of high recombination immediately 5' to HBB on chromosome 11. These β^S haplotypes may be associated with differences in SCD severity and phenotypic expressions including PH [33]. A more refined understanding of the haplotype differences flanking the sickle mutation would therefore be helpful in parallel with searches for other genetic modifiers in SCD related PH. As such, in this study patients consenting to participate in the genetic portion of this study will undergo an extensive genome wide genetic analysis followed by segments of regions associated with PH. When available in the next five years, whole genome sequence DNA on this population samples will be performed. Specifically, automated DNA sequencing of candidate genes, novel PCR based assays, and “chip” based high-throughput SNP detection platforms will be conducted at the NHLBI through the Division of Intramural Research or through collaboration with other Institutes.

We will be particularly interested in evaluating a number of potential genetic risk factors for PH in individuals with SCD. Example of genes of interest are included in redox, inflammatory, cell adhesion, endothelial function, vasoregulation, vascular proliferation, cell cycle regulation pathways, as well as genes related to red blood cell function and structure and globin regulators.

3 STUDY OBJECTIVES AND PURPOSE

Our study has the following major objectives:

1. Screening to identify patients with sickle cell disease who have pulmonary hypertension and to create a cohort with an established cardiovascular phenotype linked to a biomarker and DNA repository
2. In a randomized, double-blind, placebo-controlled phase II/III trial, to determine the safety and efficacy of 16 weeks of sildenafil therapy on exercise capacity (six-minute walk distance), echocardiographic estimates of right ventricular systolic pressure, and symptoms in patients with sickle cell disease and pulmonary hypertension, defined by TRV $\geq$ 2.7 m/s
3. Via a right heart catheterization in subjects with more severe pulmonary hypertension (TRV stratum), to evaluate and compare the acute hemodynamic effects of inhaled nitric oxide and of oral sildenafil at rest in the catheterization laboratory and to determine the changes in hemodynamics after 16 weeks of sildenafil therapy

4. To evaluate prospective clinical outcomes in the subjects participating in the Observational Follow-up Study

5. To provide subjects with open-label sildenafil for up to one year after completion of the Main Interventional Trial and to evaluate the long term safety of sildenafil in this population

The primary endpoint in the Main Interventional Trial is change in six minute walk distance across the 16 week double-blind phase.

4 STUDY DESIGN AND METHODS

4.1 Overview

Up to 1000 subjects will be screened based on medical history, physical examination, laboratory testing, transthoracic Doppler-echocardiography, and 6MW test. All consented subjects in this cohort will provide plasma and serum for storage and measurement of BNP and DNA for a repository.

Patients found to have a screening TRV of ≥ 2.7 m/s and 6MW distance between 150 and 500 meters and who satisfy all inclusion/exclusion criteria will be enrolled in the 16 week, randomized, double-blind, placebo-controlled trial of sildenafil versus placebo. During the MIT baseline period, among other things, an additional echocardiogram and 6MW will be conducted. Subjects whose additional baseline evaluations satisfy the randomization criteria will be randomized to the MIT; 132 subjects will be randomized in all. Subjects will be stratified and randomized based on site and TRV stratum.

One-half of the subjects, those with TRV ≥ 3.0 m/s, will also be evaluated with right heart catheterization (RHC) before and after 16 weeks of the intervention. Study assessments will occur at weeks 6, 10, and 16 or early withdrawal. The primary endpoint is change in 6MW distance across this study phase. Secondary endpoints include non-invasive estimation of pulmonary artery systolic pressure by transthoracic Doppler-echocardiography, plasma NT-BNP levels, cardiovascular and sickle cell related symptoms and events, and quality of life scores.

Screened subjects will proceed into the Observational Follow-up Study at the point in which they either are not qualified to participate in the MIT or complete/discontinue participation in the MIT/Open-label Follow-up Phase. Subjects may be contacted every 6-12 months for up to 3 years to assess major disease-related complications, including mortality; subject follow-up during this study will be at the discretion of each clinical site.

Recruitment into the Screening Phase and MIT is expected to extend for approximately 13 months. Subjects who complete the double-blind phase will be eligible to participate in the Open-label Follow-up Phase and will be treated with sildenafil for up to 1 additional year; only AE data will be collected during this period. Subjects in the Observational Follow-up Study will continue through the end of their third study year (counting from date of signature on the Screening Informed Consent).

Figure 2. walk-PHaSST Study Design

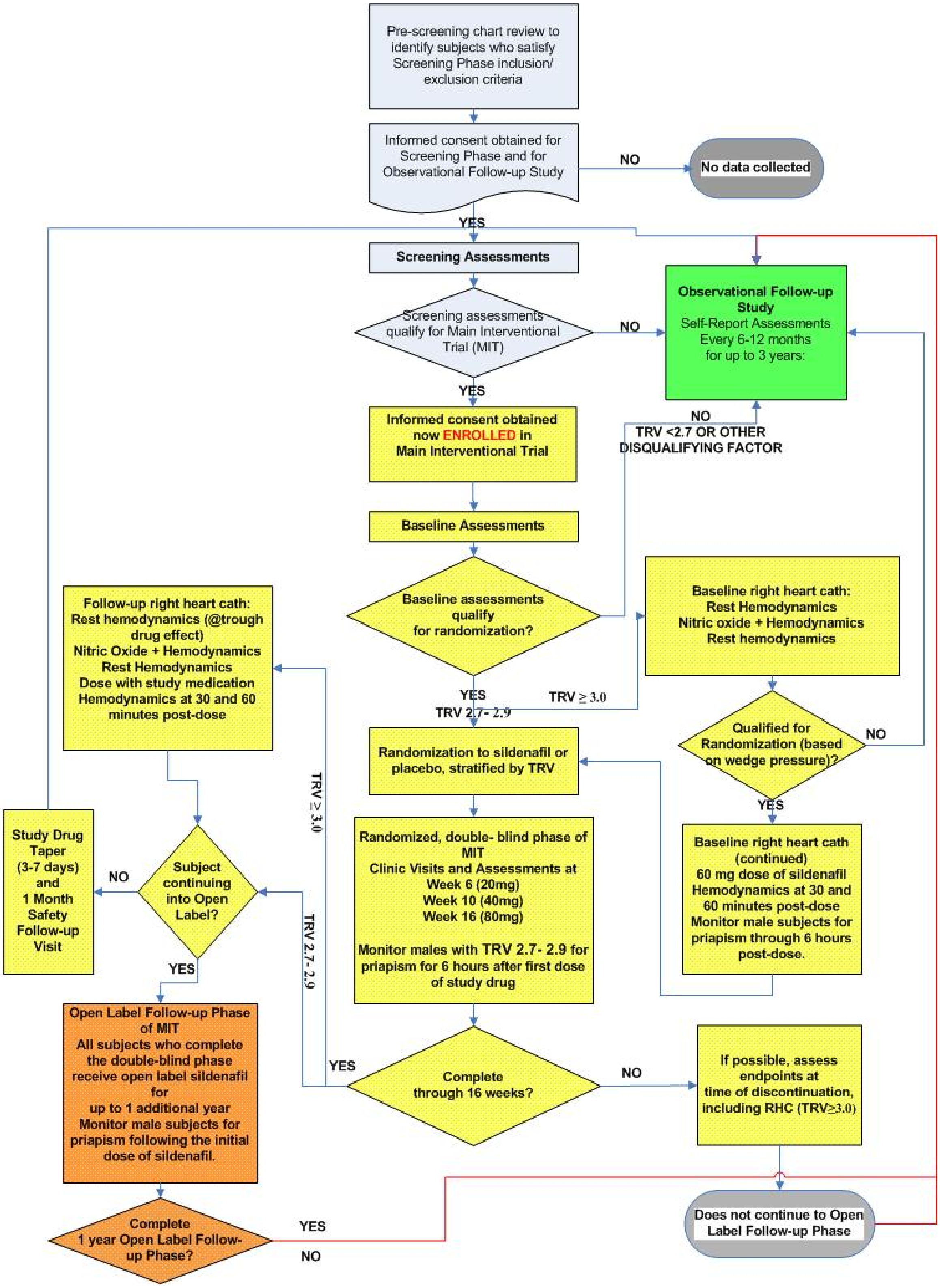

4.2 Screening Phase

Up to 1000 subjects with SCD who are not already receiving specific therapy for PH will be screened with history, physical examination, standard laboratory assessment, Doppler-echocardiogram and 6MW test. Subjects will have a Doppler-echocardiogram with specific and detailed assessment of TRV, diastolic function (e.g., E/A ratio deceleration time), and valvular and systolic function (Appendix A). Medical history, a physical exam and standard laboratory tests including documentation of SS, SC, SD, or $S\beta^{+/o}$thalassemia hemoglobin genotype, HIV testing will be performed, and blood will be collected for DNA, plasma and serum (Appendix E). NT-pro BNP levels and other specialty biomarkers will be run centrally at the NIH. All screening procedures should be performed two weeks after resolution of vaso-occlusive crisis (VOC), ACS event, or acute transfusion. This will create a cohort in which potential subjects for the MIT will be identified. See Table 3 for a complete schedule of screening assessments.

For all subjects identified with a TRV ≥2.7 m/s, evaluation for possible causes of pulmonary hypertension should be performed as clinically indicated per routine standard of care. Subjects with a history of regular loud snoring and sleepiness, including males with a neck circumference >17 inches and females with a neck circumference > 15 inches should be referred for evaluation of possible obstructive sleep apnea. Subjects newly recognized to be HIV antibody positive during the screening phase should be evaluated for anti-retroviral therapy as per routine standard of care prior to enrollment in the main interventional trial.

In order to monitor AEs that may be associated with the screening procedures, AEs will be collected if they satisfy the following criteria: 1) the AE is temporally associated with any screening phase study procedure (i.e, beginning or worsening after signing the screening informed consent through 7 days after the last screening procedure) and 2) deemed by the investigator to have a potential association with that procedure.

4.3 Observational Follow-up Study

All patients who sign informed consent for the Screening Phase are also consenting to participate in the Observational Follow-up Study. Screened subjects will proceed into the Observational Follow-up Study if a) they do not qualify for -or choose not to participate in- the MIT; b) are discontinued for any reason during the MIT or the Open-label Follow-up Phase; or c) complete the Open-label Follow-up Phase.

To the extent possible, sites will contact each OFS subject either in-person or via telephone every 6-12 months through the end of his/her third study year. This cohort will provide natural history data for the duration of the trial. The clinical data to be collected includes vital status and self-report of health care utilization, episodes of ACS or priapism, and initiation/continuation of therapies for SCD or iron overload. See Table 3 for a complete schedule of Observational Follow-up Study assessments.

Any subject in the observational follow-up cohort without an initial TRV ≥2.7 m/sec who develops apparent pulmonary hypertension detected through routine clinical care will be eligible for re-screening and participation in the MIT while it is still enrolling.

4.4 Main Interventional Trial

4.4.1 Description of Study Endpoints

The *primary efficacy endpoint* is the change in 6-minute walk (6MW) distance across the 16 weeks of therapy.

The *secondary efficacy endpoints* include the following:

- Hemodynamic parameters (right atrial pressure; pulmonary artery systolic pressure; pulmonary artery diastolic pressure; MPAP; pulmonary capillary wedge pressure; PVR; cardiac output; mixed venous oxygen saturation; systemic systolic, diastolic and mean pressures; SVR; and body surface area indexed values for cardiac output, PVR, and SVR)
- Left and right ventricular systolic and diastolic function
- NYHA/WHO functional class
- Borg dyspnea score
- Time to clinical deterioration of PH
- Clinical outcomes: number of visits to emergency departments; hospitalizations; reasons for hospitalizations; episodes of acute chest syndrome, number of transfusions
- Quality of Life score (SF-36 for ages 14-adult; Peds-QL for ages 12-13)
- Pain score (BPI)

The *safety endpoints* include the following:

- Vital signs, including blood pressure
- Change in physical examination
- Urinalysis
- CBC
- Reticulocyte count
- Peripheral oxygen saturation by pulse oximetry
- AE monitoring

- Baseline history and interim history (including pain history, pain treatment, respiratory symptoms, inpatient or outpatient evaluations, and management of SCD-related complications)
- Concomitant medications monitoring

The *genetic/biomarker assessments* include the following:

- NT-pro Brain Natriuretic Peptide levels (BNP)
- DNA sequencing

4.4.2 Description of Study

4.4.2.1 Baseline

Subjects whose screening data satisfy MIT the inclusion/exclusion criteria will be offered participation in the MIT, starting 2-4 weeks after screening. Although a TRV ≥ 2.5 m/s is associated with an increased risk of death in patients with SCD, a TRV ≥ 2.7 m/s was chosen to minimize the potential inclusion of patients with false positive (borderline) TRV values. After obtaining informed consent, subjects will be evaluated for additional medical history since screening. They will also participate in a physical examination, baseline laboratory testing, chest x-ray, and a second echocardiogram with documentation of TRV for estimation of right ventricular systolic pressure, assessment of LV systolic and diastolic function, a second 6MW test and Borg dyspnea score for baseline exercise capacity, and assessment of NYHA/WHO functional class. In the case that a subject receives an unscheduled blood transfusion, all study assessments should not be performed until after 2-3 weeks have passed (i.e., 14-21 days). Additional baseline assessments include completion of the Quality of Life assessment and completion of a one-week pain diary composed of daily assessment using the Brief Pain Inventory (BPI). See Table 4 for complete list of baseline assessments.

Subjects will be categorized into 1 of 2 strata, based on their TRV as assessed during the baseline echocardiogram: TRV ≥ 2.7-2.9 m/s and TRV ≥ 3.0 m/s.

4.4.2.2 Right Heart Catheterization (RHC) Procedure

Subjects enrolled in the trial with TRV ≥ 3.0 m/s (at least moderate PH) will also undergo RHC (using standard techniques and while the subject is in steady state) at baseline and after 16 weeks. Following premedication, RHC is performed under local anesthesia with a heparin-bonded thermodilution Swan-Ganz catheter inserted percutaneously into the right internal jugular or a femoral vein depending on anatomic landmarks. Catheter is positioned in the pulmonary artery with pressure monitoring. Catheter tip position will be evaluated by fluoroscopy or chest radiography. Fluoroscopy may be used according to standard clinical practice. A 20-gauge heparin-bonded cannula inserted percutaneously into a radial, brachial or femoral artery may be used for blood sampling and monitoring of systemic arterial pressure. Mean vascular pressure levels are determined by electronic integration of the pressure signals. Heart rate and vascular pressures are monitored

continuously. Cardiac output is measured in triplicate by thermodilution or by the Fick method with measured oxygen consumption. Systemic and pulmonary vascular resistances are calculated using the mean cardiac index and mean vascular pressures.

In subjects undergoing right heart catheterization the acute hemodynamic effects of inhaled NO and oral sildenafil will be assessed at rest (see Table 2 and Appendix C) at baseline and at the end of the study.

Table 2. Inhaled NO and Oral Sildenafil Challenge at Baseline

	Intervention	Duration	Assessment
Step 1	Baseline		Resting hemodynamics
Step 2	Inhaled NO 40 ppm	10 minutes	Resting hemodynamics[1]
Step 3	None	10 minutes	Resting hemodynamics
Step 4	Sildenafil 60 mg orally	30 minutes post oral dose	Resting hemodynamics
Step 5		60 minutes post oral dose	Resting hemodynamics

[1] Resting hemodynamics assessed at the end of the 10 minute period of inhaled NO, while the subject is still inhaling the NO.

Right heart catheterization will be repeated at the end of the double-blind phase. This hemodynamic assessment will follow the guidelines below:

- Subjects will take their nighttime dose of study medication, but will not take their morning dose prior to right heart catheterization.
- Subjects will bring study medication to visit.
- Study medication is administered with direct observation by study coordinator
- Right heart catheterization will be performed approximately 4 hours after study medication is administered
- Baseline hemodynamic measurements will be performed as outlined in Table 2 (steps 1-3).
- Blinded study medication will be administered and hemodynamic measurements will be performed at 30 and 60 minutes post dosing as outlined in steps 4 and 5 of Table 2.

If during right heart catheterization, it is not possible to obtain an adequate reading of the pulmonary capillary wedge pressure, a left heart catheterization will be performed for the measurement of left ventricular end-diastolic pressure.

4.4.2.3 Monitoring for Priapism

Priapism is defined in this study as a sustained, painful erection lasting more than 3 hours or intermittent painful unwanted erections lasting less than 3 hours. Males will be monitored for priapism for 6 hours after sildenafil therapy in the catheterization laboratory (for subjects with TRV ≥ 3.0 m/s) or in the clinic after the first dose of study drug during the double-blind phase for subjects with TRVs between 2.7 m/s and 2.9 m/s.

In addition, all male patients will be monitored in the clinic for priapism after their first dose of sildenafil during the open label follow-up phase.

Males will be specifically monitored for incidence of priapism during their study visit interviews. Additionally, they will be instructed to contact a member of the study team at the onset of any degree of priapism and will be admitted for immediate evaluation and treatment. Each site will have a priapism management plan in place. Additionally, the study's Manual of Operations (MOO) will provide possible therapeutic interventions which will be individualized as clinically appropriate for each episode of priapism.

4.4.2.4 Randomized, Double-blind Phase

The randomized, double-blind phase of the MIT is designed to test the efficacy of 16 weeks of chronic sildenafil therapy on exercise endurance (6MW distance) and right ventricular systolic pressure (RVSp) in patients with PH and SCD. Randomization of patients meeting inclusion criteria will be stratified in two subgroups according to TRV (TRV ≥ 2.7-2.9 m/s and TRV ≥ 3.0 m/s); approximately 33 individuals will be randomized to each of the four treatment arm/PH strata combinations.

In the event of VOC, ACS or transfusions, all end-point assessments (including hemodynamic assessment) should be performed 14 ± 3 days after resolution of the acute event or transfusion.

All subjects will have their baseline data confirmed for eligibility prior to randomization. Those qualifying subjects with TRVs between 2.7 m/s and 2.9 m/s will proceed to randomization. Whereas qualifying subjects with TRV ≥ 3.0 m/s will next undergo the RHC procedure; those subjects with qualifying wedge pressures will then proceed to randomization. Subjects will be randomized to receive sildenafil 20 mg by mouth (PO) or placebo (matching in pill color and size) three times a day (TID). Study drug will be issued on an outpatient basis for six weeks with the dosage increased to 40 mg TID for four weeks and then increased to 80 mg TID for six weeks.

A peak dose of 80 mg TID was chosen to match our initial open label experience with sildenafil. This is based on the role of hemolysis-associated decreased NO bioavailability in the pathogenesis of pulmonary hypertension associated with sickle cell disease.

The dosing regimen was designed to achieve the following 2 objectives:

1. Minimize sildenafil related adverse effects such as headache and facial/periorbital edema through gradual dose escalation

2. Provide the ability to evaluate the relative efficacy of different sildenafil doses on functional capacity and pulmonary artery pressures as evaluated by echocardiogram

Clinical follow-up will occur at weeks 6 ± 1, 10 ±1, and 16 ± 1 or EOS, with the following procedures/tests:

- History
- Physical exam and vital signs
- Adverse event monitoring
- Concomitant medications
- Venous blood will be obtained for CBC, standard chemistries
- Pregnancy test
- Venous blood will be obtained for NT-pro Brain Natriuretic Peptide levels and other specialty biomarkers to be run centrally at the NIH
- Emergency department visits, outpatient/day hospital visits and hospital admissions for sickle cell disease and those for complications of PH will be recorded
- Six-minute walk (with assessment of Borg dyspnea index, and NYHA/WHO functional class) 1-2 hours after taking the morning dose of study drug
- Echocardiogram with assessment of TRV LV and RV function 1-2 hours after taking the morning dose of study drug. All echocardiograms should be performed at least one hour after 6MW test. EXCEPTION NOTE: Echocardiogram will not be assessed at week 10 ± 1
- Study drug compliance and daily pain assessment using the BPI --evaluated by subject for one week between each follow-up visit

End of the double-blind phase visit (week 16 ± 1) will also include:

- In subjects with an initial study entry TRV ≥ 3.0 m/s a second right heart catheterization
- Quality of life assessment using SF-36 Quality of Life form (ages 14-adult) or Peds-QL (ages 12-13)

In subjects with TRV ≥ 3.0 m/s clinically indicated studies (according to accepted guidelines) for the work-up of subjects with pulmonary hypertension (PH) can be ordered at the discretion of treating clinicians. These studies could include:

  - Collagen vascular screening tests (e.g., ANA, anti-centromere antibodies, anti SCL-70, antiphospholipid antibodies)
  - Ventilation perfusion scan
  - Chest X-ray (required for all MIT subjects at baseline), CT of chest
  - Overnight pulse oximetry
  - Spirometry with bronchodilation

- Lung volume
- Diffusing capacity for CO

Efficacy assessments will be done at peak dose (i.e., study medication should be taken 1-2 hours before measurements) when subjects are in a steady state.

A subset of clinical outcomes will be adjudicated by a blinded panel of experts. Adjudicated outcomes include: ACS, right heart failure, and clinical worsening.

Records of study drug used, dosages administered, and intervals between visits are kept during the study. Study drug accountability is performed on an ongoing basis by the study personnel and checked by the clinical research associate (CRA) during site visits and at the completion of the study. Subjects are asked to return all unused study drug at each visit.

To promote treatment compliance, the study personnel will be contacting the study subjects throughout the trial reminding them to administer the study drug as required per protocol. Additionally, subjects will be asked to complete a dosing diary for 1 week in-between each clinic visit.

4.4.3 Alternative Therapies during the Double-blind Phase

The following regimens will be permitted:

- Hydroxyurea therapy, if stable for at least 1 month preceding randomization and throughout the trial (no change in dose, except decrease for safety reasons)
- Long term oxygen therapy, if stable for a least 2 weeks prior to randomization
- Chronic blood transfusions, if started within at least 1 month preceding randomization and continued throughout the trial. Each efficacy assessment must occur at least 1 week before or at least 1 week after each chronic blood transfusion.
- Anticoagulants should be continued during the whole study period, if ongoing at time of entry into MIT
- ACE inhibitors, beta blockers, vasodilators (not including nitrate-based), diuretics provided they have been stable on the medication at least one month prior to enrollment in the study

Both the initiation of hydroxyurea therapy to those naïve to that treatment and tonsillectomy surgery should be delayed until conclusion of the double-blind phase of the MIT, unless deemed clinically necessary in the opinion of the investigator. Those subjects requiring either therapy during the MIT may, however, remain on study.

The following regimens are forbidden:

- Specific treatments for pulmonary hypertension (excluding acute administration during a catheterization procedure to test vascular reactivity)

- Protease inhibitor treatment for HIV
- Initiation of potent CYP3A4 inhibitor therapy (e.g., itraconazole, ritonavir, ketoconazole)
- Initiation of Chronic elective transfusion therapy during the double-blind phase of the MIT

See Section 7.1 for further information on study discontinuation for subjects requiring any of these forbidden therapies.

4.4.4 Open-Label Follow-up Phase

After the completion of the 16-week randomized phase, patients will be offered open-label sildenafil for up to 1 year. Sites will maintain clinical outcome follow-up with these patients during this time, every 1-3 months, based on each site's standard practice. To preserve the scientific integrity of the MIT, all subjects will remain blinded to their MIT treatment assignment until after the MIT database is locked. In order to maintain the blind on a per subject basis and to minimize the possibility of sildenafil related adverse effects, at the beginning of the Open-label Follow-up Phase all patients will be down titrated from 80mg TID of sildenafil or placebo to 20 or 25mg of sildenafil TID. The doses will then be titrated upward as tolerated (see section 7.2 for dose adjustment guidelines in case of adverse effects). Doses will increase by 20-25mg TID every two weeks, but a faster dose escalation regimen will be allowed if, in the opinion of the treating clinician, a patient has become symptomatic due to apparent dose down-titration. The maximum dose will either be 75 mg or 80 mg TID, depending on 20 mg or 25 mg tablet availability. During the course of this year, the CI and administrative team will make every effort to assist patients in obtaining medication from a non-study source; this could include, for instance, helping obtain insurance coverage for their drug. Patients will be informed that these efforts do not guarantee availability of open label drug at the end of the study contract.

Adverse events and study drug dosing schedules will be collected during this open-label period.

If patients choose not to participate in the Open-Label Follow-up Study, they will be required to return to the clinic for a one-month safety follow-up visit approximately 30 days after the MIT Week 16 visit. Clinical Investigators will taper study drug for these non-continuing patients upon completion of the MIT treatment phase. The taper will continue for 3-7 days, based on CI judgment; blinded study medication will be provided in a quantity sufficient to supply the maximum taper period. Tapering is not recommended for patients requiring discontinuation of the drug due to AEs.

Refer to Chapter 13.3 of the MOO for a suggested taper regimen and for additional details.

5 SELECTION OF SUBJECTS

Eligibility will be based on the following inclusion and exclusion criteria.

5.1 Screening Phase

5.1.1 Inclusion Criteria

1. Males or females, 12 years of age or older
2. Diagnosis of sickle cell disease (including, but not limited to SS, SC, SD, or Sβ°/+ thalassemia)
3. Provision of informed consent and, where applicable, assent

5.2 Observational Follow-up Study

5.2.1 Inclusion Criteria

1. Satisfaction of screening criteria
2. In the opinion of the investigator, ability to maintain follow-up contact
3. Failure to satisfy the eligibility requirements of the MIT OR discontinuation/completion of the MIT/Open-label Follow-up Phase
4. Provision of informed consent and, where applicable, assent

5.3 Main Interventional Trial

5.3.1 Inclusion Criteria

1. Males or females, greater than or equal to 12 years of age and less than or equal to 70 years of age
2. For female subjects, on a reliable method of birth control or not physically able to bear children
3. Electrophoretic documentation of sickle cell disease (including, but not limited to SS, SC, SD, or Sβ°/+ thalassemia)
4. At least mild pulmonary hypertension with TRV $\geq$ 2.7 m/s by echocardiogram
5. Six minute walk distance of 150-500 m
6. In the opinion of the investigator, ability to complete the protocol scheduled assessments during the 16 week, double-blind phase
7. Provision of informed consent and, where applicable, assent

8. Subjects with systemic hypertension must be on a stable antihypertensive regimen for greater than or equal to 90 days and a stable dose for greater than or equal to 30 days.

5.3.2 Exclusion Criteria

1. Current pregnancy or lactation
2. Any one of the following medical conditions:
   - Stroke within the last six weeks
   - New diagnosis of pulmonary embolism within the last three months
   - History of retinal detachment or retinal hemorrhage in the last 6 months
   - Non-arteritic anterior ischemic optic neuropathy (NAION) in one or both eyes
   - History of sustained priapism requiring medical or surgical treatment, unless currently impotent or on transfusion program within the last two years
   - Any unstable (chronic or acute) condition that in the opinion of the investigator will prevent completion of the study
3. Subjects taking nitrate-based vasodilators (including, but not limited to nicorandil [available in the UK only]), prostacyclin (inhaled, subcutaneous or intravenous) or endothelin antagonists. Subjects taking calcium channel blockers will be allowed to participate provided they are on a stable dose for ≥ 3 months.
4. Left ventricular ejection fraction < 40% or clinically significant ischemic, valvular or constrictive heart disease: LVEF <40% or SF < 22%
5. Subjects who are in other research studies with investigational drugs, with the exception of hydroxyurea, unless the other trial has been approved by the walk-PHaSST Executive Committee for co-participation
6. Acute or chronic impairment (other than dyspnea), limiting the ability to comply with study requirements (in particular with 6MWT), e.g., angina pectoris, intermittent claudication, symptomatic hip osteonecrosis
7. Tonsillectomies for sleep apnea within 3 months prior to randomization
8. Active therapy for pulmonary hypertension, including prostacyclin analog, endothelin-1 antagonists, or PDE-5 inhibitor
9. Protease inhibitor therapy for the treatment of HIV

10. Subjects taking potent CYP3A4 inhibitor therapy (e.g., itraconazole, ritonavir, ketoconazole)

11. Subjects who are anticoagulated **and** have proliferative retinopathy (unless they have had ophthalmologist recommended intervention (e.g., phototherapy) or have been otherwise cleared by an ophthalmologist to participate in the study)

12. Subjects with systolic blood pressure greater than or equal to 140 mmHg OR diastolic blood pressure greater than or equal to 90 mmHg. See Section 9.3.3 for qualifying blood pressure assessment instructions.

5.3.3 Randomization Criteria

1. Second 6MW distance between 150-500 meters and within 15% of the previous 6MW (Refer to Section 9.2.3)

2. Second echocardiogram with a TRV ≥ 2.7 m/s

3. For subjects undergoing right heart catheterization, pulmonary capillary wedge pressure ≤ 24 mmHg

4. An ophthalmological exam sometime within the past year for patients not being anticoagulated. A completed baseline ophthalmological exam for patients being and who otherwise qualify (see Exclusion Criteria #11)

6 RANDOMIZATION AND BLINDING

The study will randomize 132 subjects.

After successful qualification of their baseline data and the completion of the RHC procedure (for subjects with TRV ≥ 3.0), subjects will be randomized to one of two treatment arms by the internet based, secure Rho*RAND*™ system provided by the Data Coordinating Center (DCC). Subjects in Arm 1 will receive sildenafil. Subjects in Arm 2 will receive oral placebo. A computer-generated, adaptive randomization schema will be used to allocate subjects 1:1 between the two treatment arms. Randomization will be stratified by site and TRV stratum (2.7 m/s ≤ TRV < 3.0 m/s versus TRV ≥ 3.0 m/s). As the number of stratification factors is rather large (9x2x2), many strata may have fewer subjects than the block size, and a good balance can no longer be guaranteed for all of them[34]. As a result, the use of a traditional stratified, blocked randomization schedule could lead to significant imbalance in treatment allocations, both across the study and within the various strata. In order to increase the likelihood of balance in treatment allocations, subjects will be randomized using the standardized range variation[35] of the sequential allocation algorithm of Pocock and Simon[36], a minimization method. This method attempts to achieve treatment balance on several subject characteristics (i.e., site and TRV stratum in this study) simultaneously – not within separate strata. Minimization

This protocol sponsored by the
National Heart Lung and Blood Institute

consists of biasing the treatment allocation so as to minimize the total imbalance between the treatment groups on some scale[37]. The order of entry of the subjects to the various sites and in the various prognostic groups is assumed to be random. As minimization is a dynamic method that uses information on subjects already entered to allocate treatment to the next subject, and thus a continuous updating of the information related to previous treatment allocations is required, a centralized randomization system will be used.

The unmasked statistician will monitor the balance of treatment assignments across subjects on anticoagulants (defined as vitamin K antagonists and heparin). If a significant imbalance is detected, anticoagulant use will be added as stratification factor in the randomization.

Rho*RAND* will be configured to send alerts to the project administrative team when numbers in a stratum approach 66. Based on power calculations, a minimum of 64 subjects with TRV $\geq$ 3.0 m/s should be randomized. Additionally, slight over-enrollment in the 2.7 m/s $\leq$ TRV $<$ 3.0 m/s stratum may be permitted by NHLBI, if funding is available at that time.

To preserve the scientific integrity of the MIT, all subjects will remain blinded to their MIT treatment assignment until after the MIT database is locked.

Pharmacists at each site will be blinded and will utilize the blinded randomization notification from Rho*RAND* to issue appropriate study drug to each randomized subject. The Rho*RAND* system is able to provide unblinding information in case of a medical emergency where the exact study drug is needed in order to appropriately treat a subject. This information can only be accessed by a pre-authorized individual, such as the medical monitor.

The subjects, physicians, nurses, and ancillary support staff will be blinded to individual treatment assignment. The placebo formulations will be identical in appearance and taste to the active compounds (see Section 8.1).

The mechanism for unblinding in a clinical emergency will be detailed in the Manual of Operations.

7 MONITORING OF SUBJECTS AND CRITERIA FOR WITHDRAWAL OF SUBJECTS

7.1 Subject Discontinuation

Therapy will be discontinued for the following reasons:

1. Major bleeding complication including intracerebral hemorrhage or stroke, GI hemorrhage, need for major surgery.
2. One episode of severe priapism defined as priapism lasting for more than 4 hours or requiring transfusion or surgical therapy (see Section 4.4.2.3 for further details of priapism monitoring)

3. Positive quantitative blood HCG or pregnancy
4. New retinal detachment, hemorrhage or clinically significant visual change (Subjects will be instructed by site personnel to alert their local CI of any visual changes between study visits; the CI will schedule an immediate evaluation by an ophthalmologist if a randomized subject complains of changes in vision.)
5. Serious adverse events considered to be related to the study drug
6. Introduction of specific treatments for pulmonary hypertension (excluding acute administration during a catheterization procedure to test vascular reactivity)
7. Initiation of chronic transfusion therapy (in this instance, EOS assessments should occur either immediately prior to initiation of transfusions or between 2-3 weeks after the transfusion)
8. Protease inhibitor treatment for HIV
9. If an emergency clinical unblinding of treatment assignment is required
10. Initiation of potent CYP3A4 inhibitor therapy (e.g., itraconazole, ritonavir, ketoconazole)

Sickle cell related clinical deterioration, other than those requiring initiation of chronic transfusion therapy, does not require withdrawal of study medication. Likewise, clinical deterioration of PH does not necessarily require discontinuation of study medication, unless the Investigator feels it is necessary to introduce another PH medication. In any situation where subject discontinuation is not required, the Investigator may always choose to discontinue the subject based on his or her clinical judgment.Any premature discontinuation of study drug precludes participation in the Open-Label Follow-up Phase of the MIT. At the time of study drug discontinuation all EOS visit assessments should be obtained to the extent possible. Such subjects should continue to be followed for adverse events and collection of other study data to the extent possible. Assessment of study endpoints should be made blinded to subjects' study treatment as much as possible.

7.2 Dose Modification or Discontinuation Due to Expected Adverse Events: Headache or Facial/skin Edema

Dose adjustment instructions for symptomatic/dose limiting headache or facial/skin edema sufficient to warrant discontinuation of the drug (only for symptoms that are severe enough as described by subject or physician to warrant holding the drug) are provided herein.

1. If present during initial dose (20 mg) hold for one day and rechallenge, if symptoms occur upon rechallenge discontinue drug. Data collection should continue for such subjects to the extent possible.

2. If present during dose escalation (40 or 80 mg) hold drug for one day and restart at previously tolerated dose for one week. If symptoms recur, discontinue drug but continue to follow subject for collection of safety and other data. If tolerated for one week increase dose to desired level as dictated by protocol. If symptoms recur upon second increase hold drug for one day and drop dose to previously tolerated dose for the duration of the trial.

8 DESCRIPTION OF STUDY DRUG AND DOSING REGIMEN

Sildenafil is an oral therapy for erectile dysfunction and is a selective inhibitor of cyclic guanosine monophosphate (cGMP)-specific phosphodiesterase type 5 (PDE5). It is available in tablet form (Pfizer). Pfizer will provide the drug and matching placebo for this study free of charge through an Independent Research Grant Agreement.

Potential AEs include hypotension, headache, flushing, dyspepsia, nasal congestion, urinary tract infection, abnormal vision, diarrhea, dizziness, rash, myalgia, epistaxis, insomnia, parasthesia, pyrexia, and decreased or loss of hearing.

During the randomized, double-blind phase, the initial dose of study drug (20 mg every 8H PO) is given for a total of 6 weeks, followed by 4 weeks of 40 mg TID, and finally 80 mg TID for 6 weeks. All study drug should be given by mouth.

The open label sildenafil will be available in two tablet strengths: 20 and 25mg. At the beginning of the open label phase, all eligible subjects will be down titrated from 80 mg TID of sildenafil or placebo to 20 or 25 mg of sildenafil TID. The doses will then be titrated upwards as tolerated. Doses will increase by 20-25 mg TID every 2 weeks. The maximum dose will either be 75 mg or 80 mg TID, depending on 20 mg or 25 mg tablet availability.

Refer to MOO Chapter 13.3 for additional taper guidance for patients who choose not to participate in the Open Label Follow-up Study upon completion of the MIT.

8.1 Packaging, Labeling, Blinding, Storage, and Return of Study Drug

Pfizer will provide oral Revatio™ (sildenafil citrate) and matching Placebo (“Pfizer Product”) from multiple commercial batches, with differing expiration dates. Sildenafil citrate is a white to off-white crystalline powder with a solubility of 3.5 mg/mL in water and a molecular weight of 666.7. No additional dilution is required prior to administration. The product should be stored at controlled room temperature, 15º to 30ºC (59º- 86º F). The study drug is stable through the date stamped on the product label and is administered orally.

All study drug (active and placebo) will be prepared by the central pharmacist at the Program Support Center, HHS Supply Service Center, Perry Point, MD, and shipped to participating sites’ pharmacies for dispensing. Study drug will be available in capsule formulation (over-encapsulated tablets). Placebo and active study drug capsules will

appear identical. The drug supply for the randomized double-blind phase will be packaged to allow the local site pharmacists to remain blinded to treatment group. Upon randomization of each subject, the Rho*RAND* system will send a randomization notification to the local pharmacist that provides the identification number of the bottle(s) (or kit) of study medication to be assigned/dispensed to that subject.

All unused study drug will be returned to the local pharmacy for disposal according to their standard operating procedures.

8.2 Subject Compliance

Good subject compliance/adherence will be critical to the success of the study and will therefore be monitored in multiple ways. Subjects will be required to return their bottles of sildenafil or placebo at every scheduled clinic visit in order to receive their new supply of "treatment." Each site will count the number of pills returned and compare to the expected number of pills returned in order to calculate percent compliance. In addition, subjects will be instructed to maintain a study drug log for one week in-between each visit during the double-blind phase. They will record if and when they took their daily doses of study medication. Subjects whose diary or pill count suggest <75% compliance will be counseled.

9 CLINICAL AND LABORATORY ASSESSMENTS

9.1 Schedule of Study Events

Table 3 and Table 4 list events and study assessments to be performed during the Screening Phase/Observational Follow-up Study and Main Interventional Trial, respectively.

Table 3. Schedule of Events – Screening and Observational Follow-up Study

	Screening	Follow-up Study (Every 6-12 months, as possible)
Sign Informed Consent	X	
Medical history, physical examination, and vital signs (including weight)	X	
Standard laboratory tests (chemistry, CBC, urinalysis)	X	
Hemoglobin Electrophoresis	X[1]	
HIV test	X	
Echocardiogram (TRV for RV systolic function)	X	
6-minute walk test	X	
Genotype studies	X	
Pregnancy test	X	
Biomarkers (Central Labs [RBC pellets], e.g. NT-BNP)	X	
Adverse Events[2]	X	
Subject report of Hospitalizations/ED/Clinic visits		X
Subject report of Stroke / neurological events, surgical procedures, priapism, acute chest syndrome		X
Subject report of hydroxyurea therapy, pulmonary hypertension therapy, iron chelation therapy, renal replacement therapy, transfusion therapy		X

[1] Complete only for subjects who otherwise qualify for the MIT but for whom source documentation of hemoglobin electrophoresis is absent.

[2] AEs will be reported if they begin or worsen from the time a subject signs informed consent for the Screening Phase through 7 days after the last screening procedure and only if the investigator believes the event to be possibly associated with that study procedure.

Table 4. Schedule of Events – Main Interventional Study

		Randomized, Double-blind Phase			Open-Label Follow-up Phase[8]
	Baseline	6±1wk	10±1wk	16± wk / early term	
Sign Informed Consent	X				
Interim medical history, physical examination, and vital signs	X	X	X	X	
Standard laboratory tests (chemistry, CBC, urinalysis, including international normalized ration (INR) for subjects on vitamin K antagonist and activated partial thromboplastin time (aPTT) for subjects on therapeutic doses of unfractionated heparin)	X	X	X	X	
Echocardiogram (LV systolic/diastolic function)	X[1]	X		X	
Right heart catheterization – Hemodynamic parameters at rest [2]	X			X	
6-minute walk test	X[3]	X	X	X	
Borg dyspnea index	X	X	X	X	
NYHA/WHO functional class assessment	X	X	X	X	
Pregnancy test	X	X	X	X	
Ophthalmologic Exam	X[9]				
Chest X-ray [7]	X				
O_2 saturation	X	X	X	X	
Quality of Life (SF-36 or Peds-QL)	X			X	
Biomarkers (Central Labs, e.g. NT-BNP)	X	X	X	X	
Symptoms documentation: hospitalizations, ED, Clinic, transfusions, ACS, priapism	X	X	X	X	
Prior/concomitant medications & narcotics	X	X	X	X	
Compliance evaluations		X[5]	X[5]	X[5]	
Pain Questionnaire (BPI)	X	X[5]	X[5]	X[5]	
Adverse Events	X	X	X	X	X[6]

[1]Confirmatory (may be the second echo that confirms the screening echo).
[2]Only for subjects enrolled in the trial with TRV ≥ 3.0 m/s.
[3]Confirmatory (<15% change from screening 6MW test distance).
[4] Subjects will maintain a diary for 1 week between each study visit. The diary will capture study drug compliance and self-reported pain assessments.
[5]Monitoring interval every 1-3 months, based on each site's standard practice.
[6] If obtained with 3 months of signing the main study consent document, this test need not be repeated.
[7] Patients who complete the treatment phase of the MIT but who choose not to participate in the Open-Label Follow-up Study will have study drug tapered for 3-7 days and return to the clinic for a safety follow-up visit approximately 30 days after completing MIT.
[8]Required for qualifying anti-coagulated patients and for those with no available record of an exam within the past year. Ophthalmologic exams will provide baseline information for clinicians.

9.2 Efficacy Evaluations

9.2.1 Echocardiography

Transthoracic echocardiography will be performed according to the guidelines of the American Society of Echocardiography. Transmitral flow, Doppler determinations of the severity of valvular regurgitation, and left ventricular stroke volume will be assessed and graded. Peak velocities of the E wave and A wave, the ratio of the E wave to the A wave, and the deceleration time will be measured. Isovolumic relaxation time will be measured as the time from aortic valve closure to the start of mitral inflow. See Appendix A for Echo checklist.

Tricuspid regurgitation will be assessed in the parasternal right ventricular inflow, parasternal short-axis, and apical four-chamber views, and a minimum of five sequential complexes will be recorded. Continuous-wave Doppler sampling of the peak regurgitant jet velocity will be used to estimate the right-ventricular-to-right-atrial systolic pressure gradient with the use of the modified Bernoulli equation (4 x [tricuspid regurgitant jet velocity]^2). Pulmonary-artery systolic pressure will be quantitated by adding the Bernoulli-derived pressure gradient to the estimated mean right atrial pressure.

The mean right atrial pressure will be calculated according to the degree of collapse of the inferior vena cava with inspiration: 5 mm Hg for a collapse of at least 50 percent and 15 mm Hg for a collapse of less than 50 percent.

During follow-up visits echocardiography should be performed on peak study medication dose i.e. 1-2 hours after study medication intake.

All echocardiograms will be completed at the local site and will be sent (either via FedEx or by posting to a secure website) to Dr. Vandana Sachdev’s echocardiogram central laboratory. All echocardiograms will be de-identified (removal of all of the patient’s personal information) prior to them being sent for review and quality control. Refer to Chapter 4 of the walk-PHaSST Manual of Operations.

9.2.2 Right Heart Catheterization

The pulmonary artery catheter is a triple lumen central venous catheter with two lumens devoted to the pressure transduction functions. The distal port will transduce pressure from the pulmonary artery and from the left atrium, when in the “wedge” position. The central venous port transduces right atrial pressures. During catheter placement, right ventricular pressures are transduced. Pulmonary artery systolic and diastolic pressures, right atrial pressure, pulmonary capillary wedge pressure, systemic artery systolic and diastolic pressures, cardiac output, and calculated systemic and pulmonary vascular resistances will be obtained using this methodology.

Baseline and endpoint RHC procedures should be conducted under similar conditions (e.g., level of sedation and oxygen). Endpoint RHC should be conducted at trough levels of study drug: approximately 4 hours post-dose.

9.2.3 6-Minute Walk

The 6MW will be performed in accordance with standard practice [29, 38-40]. Briefly, site personnel will discuss the 6MW test with each patient, and will then allow each patient to complete one practice walk before the Screening 6MW[40]. The practice walk is not required if the patient has completed a documented (in the medical record) 6MW within 3 months of the Screening 6MW. The subject will be encouraged to walk as fast as possible during the preliminary instructions. However, once the walk begins the technician may not provide additional encouragement. Distance walked, Borg dyspnea index, NYHA functional class, and oxygen saturation will be determined (see Appendix D). Once the practice walk is completed, the site personnel will conduct the actual Screening 6MW ≥ 2 hours after the practice walk. Upon enrollment into the MIT, at least one additional 6MW test will be completed with each patient prior to Randomization. No additional practice walks will be conducted during the MIT.

The 6MW test performed at baseline must satisfy the following requirements:

- The 6MW test #2 distance should be within 15% of 6MW test #1 distance or a third test is required (6MW test #3). If screening and baseline 6MWs take place on the same day, the 6MW test #1 and 6MW test #2 must be done at least 2 hours apart.
- The 6MW test #3 distance should be within 15% of 6MW test #2 distance or the subject cannot be included in the study. The 6MW test #2 and #3 must be done at least 2 hours apart.
- For the subjects treated with long-term oxygen therapy, the walk test should be performed without supplemental oxygen. If interruption of the oxygen supply is deemed as unsafe and it is required during the baseline 6MW, follow-up 6MWs must be conducted at the same flow rate.

During follow-up visits 6MW tests should be performed on peak study medication dose i.e. 1-2 hours after study medication intake.

9.2.4 Clinical Outcome Adjudication

A subset of clinical outcomes that occur during the randomized, double-blind phase will be adjudicated by a blinded panel of experts. Adjudicated outcomes include: ACS, right heart failure, and clinical deterioration of PH.

In order to determine clinical deterioration of PH, the adjudication panel will consider, at a minimum, the following:

- 20% decrease in six-minute walk distance, on two successive evaluations taken at least 2 weeks apart while not in VOC, plus deterioration in NYHA/WHO functional class
- Initiation of additional PH specific therapy, that is, prostanoid analogue, endothelin receptor antagonist or PDE 5 inhibitor

9.2.5 Candidate Gene Analysis

The intention of collecting DNA samples in this population is to perform extensive genome wide genetic analysis followed by segments of regions associated with pulmonary hypertension. When available in the next five years, whole genome sequence DNA on this population samples will be performed. Specifically, automated DNA sequencing of candidate genes, novel PCR based assays, and "chip" based high-throughput SNP detection platforms will be conducted at the NHLBI through the Division of Intramural Research or through collaboration with other Institutes.

For the purpose of the genetic study design cases will be recruited as specified in the protocol (individuals meeting criteria for a diagnosis of SCD and PH). Individuals with SCD not meeting criteria for PH will serve as a first control group, and will be used to identify susceptibility determinants for PH as a whole.

Candidate genes for detailed examination will be selected from a list of cytokines, adhesion molecules, and genes identified by microarray expression analysis that are potentially involved in the pathogenesis of SCD pulmonary hypertension. Specific candidate genes to be studied are listed in Appendix E.

9.3 Scientific and Safety Evaluations

9.3.1 Biomarker Analyses

All subjects will provide blood for the biomarkers studies. Approximately 30 mL of blood will be needed for the set of assays on a given day (see Table 5). The baseline sample (Day 1) will be drawn at study entry before study drug is administered. Samples will be processed and shipped from participating sites to the NHLBI sample repository, as well as shipped to the NHLBI central laboratory for evaluation of the pro Brain Natriuretic Peptide levels.

Table 5. Investigational Laboratory Studies (Biomarkers)

Biomarker Assessed	Screening	Baseline	Week 6	Week 10	Week 16
NT pro Brain Natriuretic Peptide levels	X	X	X	X	X
Plasma for storage	X	X	X	X	X

9.3.2 Clinical Laboratory Tests

Clinical laboratory tests (including the pregnancy test, if applicable) will be performed on blood samples collected at screening and all clinic visits for the Main Interventional Trial. Urinalysis laboratory tests will be performed on urine samples collected at screening and all clinic visits for the MIT as well. The following laboratory tests will be performed:

- Liver function tests (AST, ALT, albumin, total protein, total bilirubin, alkaline phosphatase)
- Renal function tests (potassium, sodium, chloride, bicarbonate, blood urea nitrogen (BUN), and creatinine)
- Mineral panel tests (magnesium, phosphate, calcium)
- CBC (Hemoglobin, hematocrit, RBC, MCHC, mean cell volume (MCV), reticulocyte counts, white blood cell (WBC) count, platelet count, and ANC)
- LDH
- INR values for subjects who are on vitamin K antagonists (e.g. warfarin)
- Activated Partial Thromboplastin Time (aPTT) for subjects who are on therapeutic doses of unfractionated heparin

9.3.3 Physical Examination and Vital Signs

A physical examination and set of vital signs will be assessed at each clinic visit. Vital sign measurements include weight, diastolic and systolic blood pressure, heart rate, and temperature.

To determine whether the subject's blood pressure qualifies him/her for the MIT, have the subject rest for at least 5 minutes prior to the assessment. Up to 3 blood pressures may be taken. Once an assessment qualifies on BOTH the systolic and diastolic, no further assessments are needed, and the subject qualifies for the study per exclusion criterion #12. The intent is to exclude patients who have uncontrolled systemic hypertension, rather than to exclude patients in a temporary/transitory state with a one time measurement out of normal range (e.g., "white coat hypertension"). Therefore, if the clinician believes, based on experience with the subject, that the blood pressure assessment is not reflective of the subject's actual level of systemic blood pressure, the assessment may be repeated.

9.3.4 Medical History

At screening, the subject's medical history will be recorded including: previous treatment for SCD, previous ACS events, previous strokes, pain crises frequency and usual management, history of priapism, previous transfusions and reactions to transfusions and to medications.

The subject's interim medical history will be recorded at baseline and every clinic visit of the MIT, focusing on emergency department (ED) visits, day clinic visits, hospitalizations, self-medicated crises, and analgesic usage (see Appendix B for sample data collection tool).

10 DATA MANAGEMENT

10.1 Source Documentation

All clinical data entered into the electronic Case Report Form (eCRF) must be supported by signed source documentation maintained at the clinical site. Specifics of source documentation maintenance will be detailed in the Walk-PHaSST Manual of Operations.

10.2 Data Management

The site study coordinator will enter clinical data into the CRF for each subject via the secure internet access, Rho Electronic Data Capture System (Rho*EDC*™). A CRF completion guide will be available online to assist in correct CRF completion. On all other study documents, subjects will be identified by a subject number assigned at enrollment, and will not be identified by name.

Using this system, clinical site personnel will use an internet browser (Internet Explorer or similar) to key data into an electronic CRF. The screens will be accessible via the Walk-PHaSST website and require site-specific user ID/password privileges. Univariate data validation tests will be performed as the data are keyed; most implausible data values will be resolved immediately.

Data will not be stored on the site's computer. At the end of each "page," data will be submitted to DCC's secure web server using SSL (128 byte public key encryption methodology) and stored in the study's "operational database." (The database used for capturing, validating, updating, and storing the data is called an "operational database"). The database will be backed up nightly; backup tapes will be saved in a secure, off-site location. Authorized site personnel may log in to the system, review and correct previously entered data, key additional data, or lock records to prevent further inadvertent modifications at any time.

10.3 Staff Training

Prior to the onset of enrollment, clinical study coordinators and data coordinators will be centrally trained to ensure adherence to the protocol and assure the highest possible data quality. Training will be led by a combination of investigators and other staff from the clinical sites, the DCC, and NHLBI. Training presentations will address informed consent procedures, study operations and protocol requirements, data collection procedures, maintenance of source documentation, CRF completion and review, routine reporting requirements, data entry and management, and NHLBI policies and procedures. As needed and as time allows, in-person training will be provided by DCC staff as part of periodic site visits.

10.4 Data Monitoring

After training, site study coordinators will have the responsibility of monitoring CRFs and source documents for accuracy, protocol compliance, subject safety, and adherence to guidelines outlined in the Manual of Operations.

As referenced above, sites will be visited by a DCC CRA or qualified designee. At each site visit, recruitment guidelines and study eligibility criteria will be reviewed. As the study progresses, completed data forms may be reviewed during site visits and compared to source documentation (medical or site records) to confirm accuracy.

11 STATISTICAL METHODS

11.1 Analysis Populations

This study is designed to enroll sickle cell disease subjects diagnosed with PH.

Intent-to-Treat (ITT) / Safety population (SAF): All randomized subjects will be included in the ITT/SAF population. Subjects in the ITT/SAF population will be classified according to the treatment group to which they were randomized, regardless of what study drug they received.

Per-Protocol (PP) population: The PP population consists of all subjects from the ITT/SAF population who had no major protocol violations and who were followed until discharge from the study.

The primary efficacy analysis will be an intention-to-treat analysis. Secondary analyses of efficacy and safety will be performed on the ITT/SAF population, and many of the secondary efficacy analyses will be repeated on the PP population.

11.2 Efficacy Analyses

11.2.1 Primary Efficacy Endpoint

The objective of the *primary efficacy endpoint* analysis is to demonstrate through a formal hypothesis test that treatment with 16 weeks of sildenafil provides greater improvement in six-minute walk (6MW) distance than does placebo in subjects with sickle cell disease and PH.

The primary efficacy endpoint for the main study is the change in 6MW distance across 16 weeks of therapy. Missing values will be imputed based on the procedure detailed in section 11.5.5. In essence, the imputation methodology is a combination of Last Observation Carried Forward (LOCF) and the imputation of worst value observed or worst measurable value, depending on reason for discontinuation and on the availability of an assessment at the time of discontinuation. LOCF will be used to impute data for 1) subjects who are lost-to-follow-up and 2) subjects who prematurely withdraw from the randomized-double blind phase (for any reason) but complete the 6MW at the time of discontinuation. On the other hand, if a subject dies, the 6MW distance at 16 weeks will be set to zero meters. Finally, if a subject discontinues due to clinical deterioration, then the lowest observed 6MW value within that subject's treatment group and TRV stratum will be imputed as the 16 week value (see section 11.5.5 for additional details on imputation.)

The primary efficacy analysis is an Analysis of Covariance Analysis (ANCOVA) on 6MW distance change from baseline to Week 16 on the ITT Population. The primary

This protocol sponsored by the
National Heart Lung and Blood Institute

hypothesis test will be based on a test that the average change differs between the two treatment groups, with baseline 6MW distance and TRV stratum used as covariates. This type of model controls for any impact of baseline 6MW on the treatment effect without assumptions about the slope of the relationship between the baseline and Week 16 measures.

Secondary analyses of the primary efficacy endpoint are intended to demonstrate that the primary analysis results are robust. Analyses include:

- Replication of the analyses described above on the PP population
- A single two-sample Wilcoxon rank-sum test (the nonparametric alternative to the two-sample t-test) to compare the change from baseline to Week 16 in the sildenafil arm to the change from baseline to Week 16 in the placebo arm for the 6MW distance. For those subjects with missing Week 16 measurements, these measurements will be imputed using a last observation carried forward (LOCF) approach or by using the last possible measurement (see section 11.5.5).
- An Analysis of Variance (ANOVA) model which is a small deviation from the t-test approach and the ANCOVA model. This method will use the change from baseline to Week 16 with missing values imputed by LOCF or by using the last possible measurement (see section 11.5.5), as the primary endpoint. The analysis will be based on a cell-mean model with the four treatment-by-TRV-strata groups as the cells. The primary hypothesis will be based on a test that the average change across the two strata differs between the two treatment groups. The hypothesis will be tested with an F-test with 1 numerator and N-4 denominator degrees of freedom, where N is the number of subjects in the ITT population.
- A linear mixed model, which will incorporate the 6MW measurements from the baseline, Week 6, Week 10, and Week 16 assessments. This model allows for comparison of the 6MW distance over the two treatment groups, controlling for time, TRV stratum, and baseline 6MW distance as fixed effects and subject as a random effect. This analysis uses likelihood-based estimation, and subject-specific effects and correlations between the repeated measurements are modeled via the within-subject error correlation structure. All available data will be used to compensate for the data missing on a particular subject.

11.2.2 Secondary Efficacy Endpoints

The objectives of the analyses of the *secondary efficacy endpoints* are to:

- Evaluate descriptively and with model based estimation and informal hypothesis tests the effects of sildenafil therapy on sickle cell patients diagnosed with PH on multiple outcome measures including hemodynamic parameters, standard clinical measures of PH, and quality of life measurements
- Gain through descriptive and exploratory analyses a better understanding of what clinical and demographic factors might affect the efficacy of sildenafil for PH in this population

- Provide age and gender prevalence estimates for pulmonary hypertension in sickle cell patients

Secondary efficacy endpoints of the main trial will be analyzed as follows:

- Continuous outcome measures (e.g. hemodynamic parameters (RVSP), change in TRV, change in 6MW distance on sildenafil 20 mg vs. 40 mg and 80 mg in the group receiving active drug during the placebo controlled study) will generally be analyzed based on a combination of descriptive summaries and model-based analyses using models like that described for the primary endpoint analysis. At-visit-values and change from baseline values will be analyzed and reported. Descriptive, model based analyses will then be generated based on both analysis of variance type models (ANOVA) and analysis of covariance type models (ANCOVA) that control for both demographic and clinical parameters of interest (e.g. baseline NYHA/WHO functional class or baseline Borg dyspnea).
- Time to clinical deterioration of PH will be based on decisions of a blinded adjudication panel (see Section 9.2.4) and will be assessed by Kaplan-Meier methodology. These assessments will utilize log rank tests based on the Kaplan-Meier estimates used to test for differences between the two treatment groups. For this time to event outcome, Cox proportional hazards models will be used to assess whether clinical factors (e.g., TRV stratum, baseline NYHA/WHO functional class) act as confounders or effect modifiers of the treatment effect.
- The effect of treatment on the categorical measures of Borg dyspnea scale, BPI score, quality of life score, and NYHA/WHO functional class (all ordinal measures), will be examined descriptively. For each of these ordinal measures, both cross-sectional descriptions of the outcome at each time point and shift-tables from baseline will be developed to compare treatment arms descriptively. To generate informal hypothesis tests Mantel-Haenszel procedures will be used to assess for outcome differences at Week 16 and end-of-study controlling for baseline levels of the measure and TRV stratum.
- Differences across treatment groups for the clinical endpoints of death, episodes of acute chest syndrome, emergency department visits, outpatient/day hospital visits, hospitalizations, and transfusions will be analyzed using Fisher's exact test, if the number of events is sufficient. Otherwise, these data will be summarized by treatment group.

In the final report, all continuous efficacy measures (e.g. 6MW distance, hemodynamic parameters, quality of life assessments) will be summarized by N, mean, median, standard deviation, minimum, and maximum. Statistics used to summarize categorical variables (e.g. NYHA/WHO functional class) will include number and percent in each category. All efficacy measures will be summarized by treatment group, TRV strata, and treatment by TRV strata.

11.3 Safety Analyses

All safety analyses will be performed using the ITT/SAF population. Safety variables will include laboratory variables and adverse event information. Specific endpoints that will be examined include:

- Incidence of AEs between baseline and Week 16
- Incidence of SAEs between baseline and Week 16
- Mortality incidence

The primary safety outcomes for the MIT will be all AEs; the subset of AEs defined as SAEs; and mortality. Treatment emergent AEs (those that begin or worsen after start of study drug), drug-related AEs, SAEs, and drug-related SAEs (as determined by the investigator) will be categorized by body system and MedDRA (Version 6.0 or later) preferred term.

The assessment of safety data will be based mainly on the tabulation of AEs, by TRV stratum and treatment arm. Additionally, AEs will be tabulated by severity and relationship to drug. Serious AEs will be described in detail. Comparisons across treatment groups, stratifying on TRV stratum will be made using a Cochran-Mantel-Haenszel chi-square test (or Fisher's Exact test as appropriate) on any AEs that occur in more than 10% of the subjects.

Additionally, continuous blood chemistry and hematology laboratory measurements will be categories as normal/abnormal and summarized using shift tables, which describe changes in the values across visits.

11.4 Interim Analyses

No formal interim analyses will be performed in this study.

11.5 Statistical Considerations

11.5.1 Covariates

The primary analyses will be conducted adjusting for baseline 6MW distance and TRV stratum, which is a stratification variable for the study design. Baseline laboratory measurements will be controlled for when analyzing laboratory outcomes. No other covariates will be used for adjustment in the primary analyses. For all model based secondary analyses, for which preliminary descriptive analyses provide evidence of a difference among treatment groups in a baseline demographic or clinical characteristic, these characteristics will be included in the models to assess whether they act as confounders or effect modifiers. If the estimate of the effect of the characteristic in the model is significant then it will remain in the model.

11.5.2 Multi-center Studies

This study will be conducted in approximately 9 clinical sites. Descriptive measures will be used to assess whether the treatment effect differs by site for primary and secondary variables, but no formal tests will be conducted.

11.5.3 Multiple Comparisons and Multiplicity

The primary formal hypothesis test will be conducted using an alpha on a two-sided test of 0.05. Because only a single hypothesis test will be tested and other reported p-values will be considered descriptive, no adjustment for multiple comparisons will be made. Informal hypothesis tests conducted as a part of secondary analyses will all be two-sided tests, and the p-values from these tests will be presented as descriptive measures of strength of evidence. These descriptive p-values will be presented for differences between treatment groups in summary tables for secondary exploratory analyses.

11.5.4 Examination of Subgroups

Primary and most secondary efficacy analyses will be stratified by TRV stratum (2.7 m/s ≤ TRV < 3.0 m/s, or TRV ≥ 3.0 m/s). If warranted, subgroup analyses by TRV stratum may be performed to examine differences in treatment effect. Any subgroup analysis will be performed as an exploratory analysis and represented as such.

11.5.5 Handling of Dropouts and Missing Data

Investigators should make every effort to obtain end of study assessments when subjects discontinue from the study due to clinical deterioration.

For the ANOVA, ANCOVA, and the Wilcoxon rank sum test, missing data at the Week 16 assessment will be derived from predefined replacement rules:

- *Last Observation Carried Forward (LOCF).* Subjects without an assessment at Week 16 due to missing data (e.g. lost to follow-up) will have their last 6MW distance, Borg dyspnea index, and WHO functional classes carried forward (LOCF). Additionally, LOCF will be used for those who discontinue early for any reason, but who participate in a study assessment at the time of withdrawal.
- *Worst measurable value and worst observed value.* A subject who is missing endpoint values at 16 weeks because of discontinuation of study medication due to clinical worsening or death will be assigned values equal to or worse than the worst values recorded in a subject who does not discontinue study medication. Subjects who die will be assigned the worst value possible based on the scale of the endpoint (e.g., 0m for the 6MW, 10 for the Borg dyspnea and FC IV), whereas subjects who discontinue due to clinical worsening and who also do not participate in an end of study assessment of that endpoint will be assigned the worst value from that subject's treatment group and stratum.
- No hemodynamic parameters will be imputed.

No data imputation will be performed for the linear mixed model analyses, and no data will be discarded. For persons who withdrew from treatment early, this type of analyses

have been designed to use the data obtained to the point that they were lost. For these secondary analyses, missing data will be treated as randomly missing data.

11.6 Sample Size

This study will randomize 132 subjects (66 to sildenafil and 66 to placebo). Sample size was determined based on the following considerations.

The measurement of primary interest is the change in 6MW distance from baseline to week 16. To estimate the trial sample size for the primary endpoint, we used data from Ghofrani's evaluation of the effects of sildenafil in subjects with chronic thromboembolic pulmonary hypertension. [26] Ghofrani's study showed an average change from baseline of 53.7m in 6MW distance with a standard deviation of 67m after approximately 6 months of sildenafil treatment.

A t-test was used to compare the difference in change from baseline 6MW distance between the sildenafil and placebo treatment groups. In order to calculate the sample size the following assumptions were made:

- No subjects discontinue because of clinical worsening or death
- No subjects dropout prior to week 10 for other reasons
- Sixteen-week change in 6MW distance is normally distributed in both treatment groups
- Sixteen-week change in 6MW distance is 40m in subjects on sildenafil and there is no change in subjects on placebo (i.e., a difference of 40m between the two treatment groups)
- Standard deviation of change from baseline between 62 and 72m for the 16-week change in each of the treatment groups
- Power ranges from 80% to 90%
- Two-sided t-test with alpha=0.05

Table 6 presents the number of subjects needed per treatment arm for the range of assumed standard deviations and powers, thus the total number is 2 times the numbers presented in each cell.

Table 6. Sample size needed per treatment arm by assumed standard deviation and power

Change from baseline (6MW)	Standard deviation (m)	Power 90%	Power 85%	Power 80%
Mean 40m	62	52	45	39
	67	60	52	46
	72	70	60	52

If we choose the standard deviation of change from baseline determined in Ghofrani's study (67m) and a power of 90%, then we would need 60 subjects enrolled per treatment

arm. If the standard deviation were to be larger (e.g. 72m), 60 subjects per treatment arm would still result in 85% power to detect a 40m change in 6MW distance in subjects treated with sildenafil versus 0m change in placebo treated subjects. We anticipate that up to 10% of the subjects may dropout during the randomization phase prior to week 10. In order to maintain study power in the presence of potential study dropouts, we will supplement the computed sample size with an additional 10%. Based on these data, we will randomize 132 subjects into the study (approximately 33 per each of four treatment arm/TRV strata combinations).

The data presented above were also used to develop power calculations under multiple scenarios.

I. Using the two-sample t-test approach, power estimates were developed with the following assumptions

   a) 5% of subjects randomized to placebo and 2.5% of subjects randomized to sildenafil will discontinue study medication due to clinical worsening or death, and therefore will have assigned values less than the smallest change in 6MW distance in a subject who completes the study. If 6MW is normally distributed in subjects who complete the study, simulations indicated that the power is still 90%.

   b) The same result was obtained for percentages discontinuing study medication of 10% and 5% in placebo and sildenafil subjects, respectively.

II. The effect of sildenafil on 6MW distance over time was also explored using a General Linear Mixed Model (GLMM) with change from baseline as the outcome variable. The SAS® procedure MIXED was used to fit the GLMM. The GLMM included treatment group and weeks on treatment (as a class variable) as fixed effects, and subject as a random effect. This type of model allows exploration of general trends over time while controlling for correlations between repeated measurements made on each subject. The GLMM indicated a within-subject standard deviation of 47.4m and a between-subject standard deviation of 89.7m across that study. Power was calculated using mixed models under the following scenarios:

   a) Assumptions:
      - 66 subjects per treatment group,
      - 4 visit time points (Wk6, Wk10, Wk16),
      - within- and between-subject variance components obtained from the mixed model run on the Ghofrani data set,
      - mean change from baseline for the sildenafil group is 15, 25, and 40 at Wk6, Wk10, and Wk16 visit time points respectively,
      - mean change from baseline for the placebo group is 0 at all 3 post-baseline visit time points, and
      - no subjects are lost to follow up (no missing endpoint data)

      → Power estimated: 92.8%

b) Same assumptions as scenario IIa, but instead of zero subjects lost to follow-up we assume that 6% in placebo and 3% in sildenafil group are lost-to-follow-up between Wk6 and W10, and 6% in placebo and 3% in sildenafil group are lost between Wk10 and Wk16. All available data is used to compensate for the data missing on a particular subject (no LOCF procedures).
→ Power estimated: 91.1%

c) Same assumptions as scenario IIa, but instead of no lost to follow up subjects we assume that 12% in the placebo and 6% in the sildenafil group are lost between Wk0 and Wk6. All available data is used to compensate for the data missing on a particular subject (no LOCF procedures).
→ Power estimated: 90.6%

d) Same assumptions as scenario IIa, but we assume that mean change from baseline for the sildenafil group is 10, 20, and 35 at Wk6, Wk10, and Wk16
→ Power estimated: 84.9%

Now we show that the sample size calculated for the primary endpoint is appropriate for detecting significance in the secondary hemodynamic endpoint (change from baseline PA pressure) using data from the recently completed SUPER-1 trial (a randomized placebo control trial evaluating the effects of 20 mg, 40 mg or 80 mg TID of sildenafil in subjects with PH). Using the hemodynamic data from the 80 mg arm of the SUPER-1 trial with 5% two-sided significance level and 80% power, the N for mean change from baseline PA pressure is 32 subjects per group, for pulmonary vascular resistance the N is 25 subjects per group. Assuming that half of the subjects enrolled in the trial will have moderate to severe PH (TRV ≥ 3.0 m/s) and meet criteria for RHC the study will be adequately powered to ascertain the hemodynamic effects of 80 mg sildenafil.

Based on the data described herein, we will randomize 132 subjects into the study (approximately 33 per each of four treatment arm/TRV strata combinations). All randomized subjects will contribute to the primary intent-to-treat (ITT) analysis. Assuming a 15-20 % incidence of PH (defined by TRV ≥ 2.7 m/s) in adults with sickle cell disease, approximately 1000 subjects will have to be screened for eligibility to ensure a sufficient number of subjects with TRV ≥ 2.7 m/s. Subjects will be stratified and randomized for each TRV stratum and treatment arm (≥ 2.7-2.9 m/s and ≥ 3.0 m/s) with 66 subjects assigned to each treatment.

11.7 Statistical Estimates for Genetic Analysis

We will be particularly interested in evaluating a number of potential genetic risk factors for PH in individuals with SCD. The power of the study to find a significant difference between PH proportions in sickle cell patients with and without a genetic factor will depend on the numbers of cases with and without the factor, who have PH. For example, the variant VCAM1 G1238C has an estimated prevalence of 9% in both SS disease

populations and healthy African American populations in the USA. If 27 of 300 sickle cell cases have the variant and the true prevalence of PH is 30% in cases who do not have the variant, the prevalence of PH in cases who have the variant would have to be approximately 0.59 – i.e., the relative risk would have to be essentially 2 – in order for the study to have 80% power to find a significant increase. The table below shows the power of a study of 300 sickle cell patients to find a significant difference in PH prevalence between sickle cell patients with and without a genetic factor, by a z-test at a two-sided 5% level, as a function of the observed proportion of cases with the factor and the relative risk (RR) of PH in patients with the factor. True PH prevalence in the group of 300 sickle cell patients is assumed to be 30%, so that prevalence rates in patients with and without the factor depend on RR and the prevalence of the factor.

Table 7. Relative Risk Ratio

	Power (%) if relative risk (RR) of PH =		
Observed prevalence of genetic factor in sickle cell cases	1.5	2.0	2.5
5%	24	64	93
10%	38	85	99
15%	47	93	99.8
20%	54	96	99.9

The power is high (approximately 85% or higher) for genetic factors that are present in 10% or more of the sickle cell patients and have RR at least 2.

We intend for samples from this population to become a genetic archive to be used as a resource for analysis of many different genetic susceptibility factors. We have no preformed hypotheses about associations of specific genetic factors. Therefore, associations from this study will mainly serve to generate hypotheses to be tested in future studies.

12 HUMAN SUBJECT PROTECTION

12.1 Rationale for Subject Selection

Subjects of both genders will be considered for inclusion in this study. Because sickle cell anemia is primarily a disease affecting African Americans, most if not all of the subjects will be African American. Cognitively impaired and institutionalized persons will not participate in this study. Because this is a multi-Center trial, recruitment strategies may vary across sites. Subjects may be recruited through advertisement by the NIH Clinical Research Volunteer Program, the Patient Recruitment and Referral Center and through existing contacts with sickle cell individuals, support groups, and clinics. Additionally, recruitment may be a part of the general intake and may include measuring of vital signs and gathering of interim history. Criteria for exclusion or withdrawal from the study are based on the presence of other disease processes that may interfere with the interpretation of our results or situations that may be harmful to the healthy subjects.

12.2 Participation of Children

Children 12 years of age and older will be included in this study per §46.405 Research involving greater then minimal risk but presenting the prospect of direct benefit to the individual subject. "DHHS will conduct or fund research in which the IRB finds that more than minimal risk to children is presented by an intervention or procedure that holds out the prospect of direct benefit for the individual subject, or by a monitoring procedure that is likely to contribute to the subject's well-being, only if the IRB finds that:

(a) the risk is justified by the anticipated benefit to the subjects;

(b) the relation of the anticipated benefit to the risk is at least as favorable to the subjects as that presented by available alternative approaches; and

(c) adequate provisions are made for soliciting the assent of the children and permission of their parents or guardians, as set forth in §46.408.

Heart catheterization is routinely performed for the diagnosis of pulmonary hypertension in pediatric patients. In addition, several centers perform follow-up catheterizations to assess response to therapy, guide therapeutic decisions and to provide prognostic information. Therefore, this follow-up measurement is necessary to help determine the effectiveness of the treatment in this research study. Therefore, for the purposes of this research, the second heart catheterization has been classified as research related and presents the prospect of direct benefit.

12.2.1 Sickle Cell Disease and Sildenafil in Pediatric Pulmonary Arterial Hypertension

12.2.1.1 Sickle Cell Disease and Pediatric PH

Pulmonary arterial hypertension is well documented in the pediatric population and has an adverse impact in morbidity and mortality that is similar to adults. The prevalence of pulmonary arterial hypertension among children with sickle cell disease is not well known. There is emerging evidence, however, suggesting that the prevalence in children with sickle cell disease is similar to that of the adult population[41]. Further study of the optimal measure to assess the degree of pulmonary hypertension in this patient population is warranted.

12.2.1.2 Sildenafil Use in Children

Many uncontrolled studies and case series have documented the use of sildenafil in pediatric patients (from newborns to adolescents) with persistent pulmonary hypertension of the newborn, after cardiovascular surgery, and specifically in children with pulmonary arterial hypertension[42-62]. These patients have been exposed to acute or single dose of the drug or chronic treatment for up to 12 months. These studies report that sildenafil is safe and tolerated in the pediatric population and that its safety profile and prevalence of adverse events is similar to what is reported in adults. Humpl et al [63] evaluated the effects

of sildenafil in fourteen children with pulmonary arterial hypertension, ages 5 to 18 years old for 2 months. Sildenafil resulted in significant improvements in functional class, hemodynamic parameters and six-minute walk distance. In that study sildenafil was well tolerated, without drug discontinuation or changes in laboratory parameters (complete blood counts, chemistries and liver function studies). There were no changes in visual acuity or color changes in vision. One patient developed facial flushing, headache and postural dizziness, which required decrease in sildenafil dose. Two patients reported heavy menstrual bleeds with onset of menarche and two patients reported transient nose bleeds. A large pediatric multi-center randomized placebo-control trial is currently underway to investigate the use of sildenafil for pediatric pulmonary hypertension. This study has already enrolled approximately 80 patients and no adverse effects different to the ones observed in adults have been observed (personal communication Dr. Robyn Barst).

12.3 Evaluation of Benefits and Risks/Discomforts

The level of risk to the adult research participants is greater than minimal, more than a minor increment over minimal risk, with a prospect of direct benefit. This assessment is in accordance with 45 CFR 46.102.

12.3.1 Risks/Discomforts of Procedures

12.3.1.1 Arterial and Venous Angiocatheters

With intra-arterial puncture there is a small potential for local hematoma (less than 5%). A very remote risk for thrombosis and laceration of arteries and veins has been reported in the literature; however, in the NHLBI experience (approximately 1000 arterial punctures with infusion studies in more than 10 years), these complications have never occurred. In our experience in protocols #98-CC-0129, # 00-H-0031 and #04-CC-0032 we have performed arterial catheterizations in more than 150 individuals with sickle cell anemia without complication.

12.3.1.2 Central Venous Catheter (CVC) Introducer

To minimize discomfort and complications all catheters will be placed by the most experienced operators. Complications of internal jugular venous catheter placement include pneumothorax (< 0.5%), catheter tip malposition requiring fluoroscopy to reposition (<5%), arterial puncture (<1%), and regional hematoma. Very rare complications include brachial plexus injury, mediastinal hematoma, significant bleeding, and catheter malposition outside of the vasculature system [64]. The risk of infection after 24-48 hours of catheter placement is minimal.

12.3.1.3 Pulmonary Artery Catheter

Of 187 patients undergoing cardiac catheterization in the NHLBI/NIH national prospective study, there were only four adverse effects of the catheterization (arterial puncture, over sedation, and one pneumothorax)[8]. Rare complications include nonsustained and sustained atrial and ventricular arrhythmia, right bundle branch block, and catheter malposition. Many of these complications could be very serious and

This protocol sponsored by the
National Heart Lung and Blood Institute

theoretically lead to death if the treatments of these complications are not effective. We have never had a death related to a pulmonary artery placement in the 10D ICU over the last 10 years with greater than 100 PA catheter placements per year. Very rare complications include pulmonary artery laceration during catheter balloon insufflation, which could lead to death (this has never occurred at our facility and has only been reported in the literature as case reports). We have performed greater than 50 right heart catheterizations in patients with sickle cell disease with only one complication. One patient developed a delayed thrombosis and phlebitis of her right internal jugular vein one week after pulmonary artery catheter placement in that vein.

In a recently published randomized trial of 1000 patients comparing the use of central venous catheters to PA catheters for the fluid management of patients with acute lung injury, complications were uncommon and were reported at similar rates in each group: 0.08±0.01 per catheter inserted in the pulmonary artery catheter group and 0.06±0.01 per catheter inserted in the CVC group (P=0.35) [65].

Left heart catheterization (entering the artery in the groin) is associated with complications that include hematoma (bleeding leading to a painful lump), ecchymosis (large black and blue mark that eventually resolves but often causes itching), retroperitoneal hemorrhage (serious bleeding into a body cavity), pseudoaneurysm or arteriovenous fistula (injuries to the arteries) which may require minimally-invasive or surgical treatment, hemorrhage (bleeding) which may require transfusion or surgical repair. Rarely these procedures are complicated by death (less than 0.2%)

12.3.1.4 Fluoroscopy

According to dosimetry estimates calculated by Dr. James Vucich of the Clinical Center Nuclear Medicine Department, this exposure to x-ray will result in 0.216 rem to the skin.

12.3.2 Risks/Discomforts of Study Medications

12.3.2.1 Sildenafil Use in Healthy Volunteers

Cardiovascular

Single oral doses of sildenafil up to 100 mg produced no clinically relevant changes in the electrocardiograms of normal male volunteers.

Studies have produced relevant data on the effects of sildenafil on cardiac output. In one small, open-label, uncontrolled, pilot study, eight patients with stable ischemic heart disease underwent right heart catheterization. A total dose of 40 mg sildenafil was administered by four intravenous infusions.

The results from this pilot study demonstrated that the mean resting systolic and diastolic blood pressures decreased by 7% and 10% compared to baseline in these patients. Mean resting values for right atrial pressure, pulmonary artery pressure, pulmonary capillary wedge pressure and cardiac output decreased by 28%, 28%, 20% and 7%, respectively. Even though this total dosage produced plasma sildenafil concentrations, which were approximately two to five times higher than the mean maximum plasma concentrations

following a single oral dose of 100 mg in healthy male volunteers, the hemodynamic response to exercise was preserved in these patients.

Ocular Events

At single oral doses of 100 mg and 200 mg, transient dose-related impairment of color discrimination (blue/green) was detected using the Farnsworth-Munsell 100-hue test, with peak effects near the time of peak plasma levels. This finding is consistent with the inhibition of PDE6, which is involved in phototransduction in the retina. An evaluation of visual function at doses up to twice the maximum recommended dose revealed no effects of sildenafil on visual acuity, intraocular pressure, or pupillometry.

12.3.2.2 Adverse Reactions: Pre-marketing Experience

Sildenafil was administered to over 3700 patients (aged 19-87 years) during clinical trials worldwide. Over 550 patients were treated for longer than one year.

In placebo-controlled clinical studies, the discontinuation rate due to adverse events for sildenafil (2.5%) was not significantly different from placebo (2.3%). The adverse events were generally transient and mild to moderate in nature.

In trials of all designs, adverse events reported by patients receiving sildenafil were generally similar. In fixed-dose studies, the incidence of some adverse events increased with dose. The nature of the adverse events in flexible-dose studies, which more closely reflect the recommended dosage regimen, was similar to that for fixed-dose studies.

When sildenafil was taken as recommended (on an as-needed basis) in flexible-dose, placebo-controlled clinical trials, the following adverse events were reported:

Table 8. Adverse Events Reported more Frequently by Patients Treated with Sildenafil than those Treated with Placebo in PRN Flexible-Dose Phase II/III Studies

Event	Percentage of Patients Reporting an Event[1] SILDENAFIL **N=734**	PLACEBO **N=725**
Headache	16%	4%
Flushing	10%	1%
Dyspepsia	7%	2%
Nasal Congestion	4%	2%
Urinary Tract Infection	3%	2%
Abnormal Vision[2]	3%	0%
Diarrhea	3%	1%
Dizziness	2%	1%
Rash	2%	1%

[1] Reported by more than 2% of patients on sildenafil
[2] *Abnormal Vision:* Mild and transient, predominantly color tinge to vision, but also increased sensitivity to light or blurred vision. In these studies, only one patient discontinued due to abnormal vision.

Other adverse reactions occurred at a rate of >2%, but equally common on placebo: respiratory tract infection, back pain, flu-like syndrome, and arthralgia.

In fixed-dose studies, dyspepsia (17%) and abnormal vision (11%) were more common at 100 mg than at lower doses. At doses above the recommended dose range, adverse events were similar to those detailed above but generally were reported more frequently.

12.3.2.3 Adverse Reactions: Post-marketing Experience

Cardiovascular

Serious cardiovascular events, including myocardial infarction, sudden cardiac death, ventricular arrhythmia, cerebrovascular hemorrhage, transient ischemic attack and hypertension, have been reported post-marketing in temporal association with the use of sildenafil. Most, but not all, of these patients had preexisting cardiovascular risk factors.

Many of these events were reported to occur during or shortly after sexual activity, and a few were reported to occur shortly after the use of sildenafil without sexual activity. Others were reported to have occurred hours to days after the use of sildenafil and sexual activity. It is not possible to determine whether these events are related directly to sildenafil, to sexual activity, to the patient's underlying cardiovascular disease, to a combination of these factors, or to other factors (see WARNINGS for further important cardiovascular information).

Other Events

Other events reported post-marketing to have been observed in temporal association with sildenafil and not listed in the pre-marketing adverse reactions section above include the following.

- *Nervous system:* seizure and anxiety
- *Urogenital:* prolonged erection, priapism and hematuria
- *Ocular:* diplopia, temporary vision loss/decreased vision, ocular redness or bloodshot appearance, ocular burning, ocular swelling/pressure, increased intraocular pressure, retinal vascular disease or bleeding, vitreous detachment/traction and paramacular edema.

At single oral doses of 100 mg and 200 mg, transient dose-related impairment of color discrimination (blue/green) was detected using the Farnsworth-Munsell 100-hue test, with peak effects near the time of peak plasma levels. This finding is consistent with the inhibition of PDE6, which is involved in phototransduction in the retina. An evaluation of visual function at doses up to twice the maximum recommended dose revealed no effects of sildenafil on visual acuity, intraocular pressure, or pupillometry.

Characteristically mild and transient, predominantly described as color tinge to vision, but also increased sensitivity to light or blurred vision have been reported in 3% of patients enrolled in the sildenafil arm of Flexible-Dose Phase II/III Studies. In these studies, only one patient discontinued due to abnormal vision. In a recently completed 12 week randomized, double-blind, placebo-controlled study conducted in 277 patients with PH the incidence of visual complains was similar to the one described in studies of sildenafil use in erectile dysfunction [24].

Nonarteritic ischemic optic neuropathy (NAION) has been described in approximately 40 patients using sildenafil and tadalafil for erectile dysfunction [66]. All individuals either had hypertension, dyslipidemia or diabetes. In these individuals, final visual acuity in the affected eye ranged from 20/20 to light perception. It is possible that this complication could lead to blindness in the affected eye. In the general population risk factors for the development of NAION include low cup to disc ratio ('crowded disc'), age over 50, diabetes, hypertension, coronary artery disease, hyperlipidemia and smoking.

In a retrospective case (n=38) control (n=38) study males with NAION were no more likely to report a history of sildenafil or tadalafil use compared to similarly aged controls (odd ratio (OR) 1.75, 95% confidence interval (CI) 0.48 to 6.30 and OR 1.82, 95% CI

0.21 to 15.39). However, for those males with NAION and a history of myocardial infarction, a statistically significant association with sildenafil or talidafil use was observed (OR 10.7, 95% CI 1.3 to 95.8). A similar association was observed for those with a history of hypertension though it lacked statistical significance (OR 6.9, 95% CI 0.8 to 63.6) [67]. In another survey the incidence of NAION in men receiving sildenafil treatment for erectile dysfunction was estimated using pooled safety data from global clinical trials and European observational studies [68]. Based on clinical trial data in more than 13,000 men and on more than 35,000 patient-years of observation in epidemiologic studies, the authors estimated an incidence of 2.8 cases of NAION per 100,000 patient-years of sildenafil exposure. This is similar to estimates of NAION reported in general US population samples (2.52 and 11.8 cases per 100,000 men aged >or=50 years).

Using the World Health Organization definitions for causality assessment of suspected adverse reactions most reports suggest that the association between PDE-5 inhibitors and NAION is possible [69]. As such the US Food and Drug Administration (FDA) issued the following recommendation regarding the use of PDE-5 inhibitors:

> "Physicians should advise patients to stop use of all PDE-5 inhibitors, including sildenafil, and seek medical attention in the event of a sudden loss of vision in one or both eyes. Such an event may be a sign of non-arteritic anterior ischemic optic neuropathy (NAION), a cause of decreased vision including permanent loss of vision, that has been reported rarely post-marketing in temporal association with the use of all PDE-5 inhibitors. It is not possible to determine whether these events are related directly to the use of PDE-5 inhibitors or to other factors.
>
> Physicians should also discuss with patients the increased risk of NAION in individuals who have already experienced NAION in one eye, including whether such individuals could be adversely affected by use of vasodilators such as PDE-5 inhibitors."

A number of studies evaluating both acute dosing (12.5-100 mg)[27, 28, 30, 31, 70] and chronic dosing (20-100 mg TID) [24, 71-75] have been performed. These studies report no serious adverse events with only mild headache, dyspepsia, and flushing reported (see Table 3).

Table 9. Sildenafil Trials in Men and Women with Pulmonary Hypertension with Published Adverse Events

Reference	Number of Patients	Dose	Duration	Adverse Event
Zimmerman [76]	1	50 mg TID<br>50 mg QID	30 weeks	Transient headache<br>Mild dyspepsia and flushing
Jackson [72]	2	50 mg TID	6 months	None
Lepore [30]	9	50 mg X1	Single dose	None (MAP stable)
Littera [74]	1 (patient with β-thalassemia intermedia)	50 mg BID	15 months	Nasal congestion
Michelakis [31]	13 (9 women)	75 mg X1	Single dose	1 patient with headache (no change in MAP)
Watanabe [77]	2	25 – 50 mg PO BID	3 months	None
Singh [78]	2	25 mg TID – 100 mg QID	5 months	None
Abrams [79]	1<br>(4 year old girl)	2mg/kg QID	3 months	None
Ghofrani [28]	8	50 mg	Single dose	None
Michelakis[32]	5	50 mg TID	3 months	None
Ghofrani[25]	14	25 mg TID- 50 mg TID	9-12 months	None
Ghofrani[26]	12	50 mg TID	6 months	None
Galie [24]	207 on active drug	20 mg, 40 mg, 80 mg TID	12 week	Headache<br>Dyspepsia<br>Flushing<br>Back Pain

12.3.2.4 Sildenafil and Sickle Cell Disease

Overall we have treated 22 patients with SCD and PH with sildenafil. The drug was well tolerated and only 2 patients required drug discontinuation due to headaches. We have also observed the presence of eyelid edema in three sickle cell patients at either sildenafil initiation or dose escalation. This phenomenon was not reported in the literature, is transient in nature even with continuation of the drug and was not associated with any impairment in visual function.

Up to 45 % of male patients with sickle cell disease (HbSS) will develop one episode of priapism as a consequence of the disease. As such, patients SCD are advised to use sildenafil with caution. No cases of priapism were reported in clinical trials of sildenafil. Interestingly, there are no case reports of sildenafil-induced priapism with SCD. There was one report of an individual with sickle cell trait (HbAS) presenting with priapism after a 50 mg dose of sildenafil. In contrast to that report, Bialecki and Bridges [80]describe resolution of recalcitrant priapism with acute administration of sildenafil in three patients

with sickle cell disease. Interestingly in the two patients who were offered outpatient therapy, the use of sildenafil at the onset of priapism resulted in resolution of the episode.

We have treated seven males with SCD and PH with sildenafil on a chronic basis. Three were at low risk for the development of priapism (due the chronic exchange transfusion and erectile dysfunction) and 4 were at risk. One of the 4 subjects at risk developed an episode of mild priapism that spontaneously resolved in two hours without any clinical sequelae.

12.3.3 Benefits of Sildenafil Therapy

The study is intended to evaluate the safety and efficacy of sildenafil in patients with SCD and lung disease. Therefore, as in all clinical trials, the true benefit to individual patients is uncertain. Improvement in exercise capacity is the predicted benefit to those subjects on sildenafil. Moreover, we believe that the information obtained from this study will be important for better understanding the therapeutic efficacy of sildenafil for sickle cell patients with lung disease.

12.4 Protocol Consent Processes and Documents

Each subject will receive an oral and written explanation of the purposes, procedures, and risks of this study in language appropriate for the individual’s level of understanding. A copy of the signed consent/assent form will be placed in the medical record. A member of the protocol team will be available to answer questions about the study to be performed.

The study will be discussed verbally with each child in the presence of their parent(s) or guardian(s). All questions will be answered and verbal assent will be obtained. The parent(s)/guardian(s) and/or child will then sign the appropriate consent/assent document according to local IRB policy.

12.5 Patient Advocate

A patient’s right representative will be made available to patients at each study site. Patients may ask any questions about the study and may withdraw their consent at any time.

13 SAFETY EVALUATIONS AND REPORTING PLAN

13.1 Safety Assessments Overview

During the initial screening phase, subjects will be monitored for adverse events and serious adverse events.

Subjects in the double-blind phase of the Main Intervention Trial will have routine evaluations at weeks 6, 10 and 16. At each visit, subjects and/or their parents will be queried regarding recent medical events or procedures. Specific events will be documented at each visit to ascertain the nature and treatment of the event, including pain crises, episodes of acute chest syndrome, transfusions, and hospital admissions. These events and diagnoses will be followed up by the nurse coordinator, who will review

hospital charts, medical records, and office visit records for documentation in the follow-up visit forms in the CRF. Additionally, clinical laboratory data (specified elsewhere in the protocol) will be assessed to evaluate safety concerns. Subjects will continue receiving the same regimen until they have been followed for 16 weeks or until they have discontinued from the study.

During the open-label follow-up phase, subjects will be monitored for adverse events and serious adverse events at every 1-3 months, based on each site's standard practice.

Subjects in the Observational Follow-up Study may be contacted every 6-12 months and asked to report on their experiences in relation to a pre-designated list of clinical events only. These clinical events are noted in section 9.2.

13.2 Definitions

13.2.1 Adverse Events

An adverse event (AE) is defined for this study as any untoward medical occurrence in a subject or clinical investigation subject that does not necessarily have a causal relationship with the study treatment. An AE can therefore be any unfavorable and unintended sign (including an abnormal laboratory finding), symptom, or disease that began or worsened during the course of clinical study participation, whether or not related to sildenafil. AE data are recorded on the CRF.

13.2.2 Serious Adverse Events

A serious adverse event (SAE) is defined as any untoward medical occurrence that:

- Results in death;
- Is life-threatening (i.e., an event in which the subject was at risk of death at the time of the event; it does not refer to an event which hypothetically might have caused death if it were more severe).
- Requires inpatient hospitalization or prolongation of existing hospitalization.
- Results in persistent or significant disability/incapacity.
- Results in a congenital anomaly/birth defect.
- In the opinion of the investigator, is an important medical events that may not be immediately life-threatening or result in death or hospitalization but may jeopardize the subject or may require intervention to prevent one of the other outcomes listed in the definition above, may be considered serious. Examples of such events are intensive treatment in an emergency department or at home for allergic bronchospasm; blood dyscrasias or convulsions that do not result in hospitalization; or development of drug dependency or drug abuse.

13.2.3 Adverse Clinical Laboratory Trend

An *adverse clinical laboratory trend* is a shift, in an adverse direction, in the mean (or median) change-from-baseline of a clinical laboratory parameter that is more adverse in

an active treatment group than in a control group (e.g., mean ALT change-from-baseline increases significantly more in the active treatment group than in the control group). An adverse clinical laboratory trend may also be evaluated via a “shift table,” which cross-tabulates subjects’ lab results (e.g., ALT values) in three categories (below normal range, within normal range, above normal range) at baseline, by their results in the same three categories post-baseline, separately for each treatment group. An adverse clinical laboratory trend would be demonstrated by increased frequencies of shifts in an adverse direction (e.g., above-normal ALT) in the active treatment group in comparison to the control group.

13.3 Assessment of Adverse Event Severity and Relationship to Treatment

The following scale will be used to "grade" the severity of all adverse events.

1. Mild. Awareness of sign, symptom, or event, but easily tolerated; does not interfere with usual daily activities or tasks.

2. Moderate. Discomfort enough to cause interference with usual daily activity; may warrant therapeutic intervention.

3. Severe. Incapacitating; inability to perform usual activities and daily tasks; significantly affects clinical status; requires therapeutic intervention.

4. Life-threatening. Adverse event is life-threatening.

5. Death. Adverse event causes death.

The standard nomenclature for defining the causal relationship between an AE and the study drug used by the NHLBI is listed in the following table. The category that overall best "fits" the relationship between the adverse event and the study drug should be chosen and recorded on the CRF and SAE form, if appropriate.

Table 10. Relationship between Treatment and AE

Unrelated	No temporal association to study product.<br>An alternate etiology has been established.<br>The event does not follow the known pattern of response to study product.<br>The event does not reappear or worsen with re-challenge.
Probably not related / remote	No temporal association to study product.<br>Event could readily be produced by clinical state, environmental or other interventions.<br>The event does not follow the known pattern of response to study product.<br>The event does not reappear or worsen with re-challenge.
Possibly related	Reasonable temporal relationship to study product.<br>The event is not readily produced by clinical state, environmental, or other interventions.<br>The event follows a known pattern of response to the study product or as yet unknown pattern of response.
Probably related	There is a reasonable temporal association with the study product.<br>The event is not readily produced by clinical state, environmental, or other interventions.<br>The event follows a known pattern of response to the study product.<br>The event decreases with de-challenge.
Definitely related	There is a reasonable temporal relationship to the study product.<br>The event is not readily produced by clinical state, environmental, or other interventions.<br>The event follows a known pattern of response to the study product.<br>The event decreases with de-challenge and recurs with re-challenge.

13.4 Assessment of Adverse Event Outcome and Action(s) Taken

The CI will follow every AE reported on the CRF to 1) a satisfactory outcome or stabilization of the event or 2) until the trial has ended. SAEs ongoing at the end of the trial will be followed by the CI for one additional month to determine an outcome, if possible.

The CI will record each AE outcome on the eCRF. The terms used to describe AE outcome are as follows (outcome of reaction/event at the time of last observation):

- Ongoing
- Resolved or stabilized without sequelae
- Resolved or stabilized with sequelae
- Death

Both actions taken in response to an AE and any follow-up results (including lab results) will be recorded in the subject's medical record in accordance with local procedure. Any treatment administered for the AE must be recorded in the subject's CRF. When subjects are discontinued from the study due to an AE, relevant clinical assessments and laboratory tests will be repeated as necessary until final resolution or stabilization occurs.

13.5 Reporting of Adverse Events

In the screening phase, AEs will be reported if they begin or worsen from the time a subject signs informed consent for the Screening phase through 7 days after the last screening procedure and only if the investigator believes the event to be possibly associated with that study procedure.

In the MIT and Open-label follow-up phases, the study will capture AEs that begin or worsen in the period from provision of MIT informed consent through the either the last dose of study drug OR until discontinuation from the MIT (for those consented subjects who were never treated).

13.6 Reporting of Serious Adverse Events

In the screening phase, SAEs will be reported if they begin or worsen from the time a subject signs informed consent for the Screening phase through 7 days after the last screening procedure and only if the investigator believes the event to be possibly associated with that study procedure.

SAE reporting during the MIT and Open-label follow-up phases will include events with onset dates occurring any time after the subject signs MIT informed consent until 1 month after the last dose of study drug or until discontinuation from the MIT (for those consented subjects who were never treated). All SAEs with onset prior to study discontinuation will be reported on the CRF and to the DCC Product Safety Team.

Sites will report SAEs to their IRBs according to section 13.7 and to that IRB's requirements.

13.6.1 Initial Reporting of SAEs by the Clinical Investigator (CI)

After learning that a subject has experienced an SAE, the CI must report the SAE to the DCC Product Safety Associate by the end of the next business day. The CI will report the SAE to the governing IRB, according to section 13.7 and to that IRB's requirements.

The initial SAE report received from the site should include the following minimum information: the subject's study identification number; study treatment; the identification of the person making the report; the event, the CI's evaluation of the AE's relationship to study drug as well as 1) characterization (e.g., requires inpatient hospitalization > 4 days); 2) severity; 3) onset date; 4) outcome, if available; 5) actions taken; and 6) investigator comments.

The DCC Product Safety Associate will review the SAE Report, will gather additional information from the site, as necessary, and will consult with the medical monitor or his physician designee. Together, this Product Safety Team will determine 1) whether the SAE was expected and 2) whether the SAE was at least possibly related to the study drug. The Product Safety Team will also review the severity of the SAE in order to determine follow-up actions.

13.6.2 IND Safety Reporting to the FDA

Because this study will be conducted under an IND, U.S. Government regulations 21CFR, Part 312.32 require the issuance of an IND Safety Report to the Food and Drug Administration (FDA) for all "serious" adverse events that are "unexpected" and for which there is a "reasonable possibility that the experience may have been caused by the drug." Such serious events that are characterized as fatal or life-threatening must be reported to the FDA within 7 calendar days of the sponsor's initial receipt of the information, whereas non-fatal and non-life threatening events must be reported within 15 calendar days.

The list in Table 11 identifies potentially serious adverse events that are "expected" based on the underlying disease, sickle cell anemia, or on the right heart catheterization procedure. Section 13.7.2 includes additional expected SAEs based on PH and study procedures. No expedited IND Safety Report is required if the event is considered to be expected based either on the sildenafil product label, the underlying disease states (i.e., PH or sickle cell disease), or the study procedures. Additionally, a serious adverse event requires an expedited report to the FDA only if it is considered to be at least possibly related to study drug.

The Product Safety Team will issue IND Safety Reports to NHLBI for all unexpected, serious adverse events that are believed to be at least possibly related to study drug. In compliance with FDA and National Institutes of Health (NIH) regulations, the Product Safety Team will prepare a collaborative report of these qualifying SAEs using the current version of the FDA MedWatch reporting form. The form will be sent to NHLBI for review and submission to the FDA. When an SAE requiring an IND Safety Report has not resolved by the report deadline, NHLBI will send the report to the FDA, and the Product Safety Team will work with the CI's to gather follow-up reports on the event.

Table 11. List of Sickle Cell Related or Right Heart Catheterization Related Adverse Events

**Sickle Cell Disease**		
Acute chest syndrome	Ileus	Renal failure
Amenorrhea	Infection, other bacterial	Renal insufficiency
Anemia (severe)	Infection, pneumococcal	Renal papillary necrosis
Aplastic crisis	Infection, line	Retinopathy
Avascular necrosis of hip/shoulder	Infection, viral	Rhabdomyolysis
Bacteremia	Jaundice	Seizure
Bone infarction	Meningitis	Septicemia
Cardiomegaly	Nephropathy	Silent infarct
Cholecystitis	Osteomyelitis	Skin ulcer
Cognitive dysfunction	Pain, back	Splenic sequestration
Decreased renal function	Pain, chest	Splenic sequestration
Decreased lung function	Pain, joint	Splenomegaly
Delayed growth/puberty	Pain, long bone	Transfusion, unanticipated
Depression	Pain, severe abdominal	Vaso-occlusive pain
Electrolyte imbalance	Pain, sternal or rib	
Elevated serum transaminases	Proteinuria	
Fever	Pneumonia	
Hand-foot syndrome/dactylitis	Pulmonary embolism	
Hematuria	Pulmonary hypertension	
Hemiplegia	Pulmonary infiltrate on chest x-ray	
Hepatic sequestration	Pyelonephritis	
Hepatomegaly		
Hospitalization >24 hours for sickle cell related events		
Hypersplenism		
Hypertension		
Hypoxemia (PO2 < 65mm Hg)		
**Catheterization Procedure**		
Arterial puncture	Hematoma	Vasovagal reaction
Cardiovascular symptoms occurring more than 24 hours after admin of pharmacologic agents (e.g. prostacyclin, nitric oxide, oxygen)	Transient arrhythmia	
Chest or extremity discomfort	Non-sustained hypotension	

13.6.3 Safety Reporting in the United Kingdom

Reporting SAEs and of suspect, unexpected, serious adverse reactions (SUSARs) will be conducted in compliance with the European Directive 2001/20/EC.

13.7 Reporting of Adverse Events to the IRB

13.7.1 Expected AEs that Require no IRB Reporting

The following expected adverse events will be listed in the consent approved by IRB and will not be reported to IRB.

Cardiology

- Vasovagal symptoms during placement of intravascular or arterial catheters or during blood draw.
- Cardiovascular symptoms occurring less than 24 hours after administration of pharmacologic agents (e.g., nitric oxide, oxygen) during cardiac catheterization studies

Pulmonary Artery Catheterization

- Transient arrhythmia
- Chest or extremity discomfort
- Vasovagal reaction
- Non-sustained hypotension
- Hematoma
- Arterial puncture
- Conduction abnormalities or atrial/ventricular arrhythmias occurring cardiac catheterization study not requiring pacing or cardioversion/defibrillation.
- Pericardial effusion following diagnostic/research cardiac catheterization study not requiring treatment
- Pneumothorax following diagnostic/research cardiac catheterization (left ventricular puncture) not requiring treatment

6-minute Walk

- Shortness of breath
- Dizziness
- Headache

- Transient arrhythmia
- Accidental injury sustained from falling not requiring medical evaluation
- Chest or extremity discomfort
- Vasovagal reaction
- Non-sustained hypotension
- Atrial/ventricular arrhythmias occurring during study not requiring pacing or cardioversion/defibrillation.
- Accidental injury sustained from falling that requires medical evaluation
- Chest or extremity discomfort requiring treatment
- Sustained hypotension requiring treatment

Pulmonary Function Testing

- Chest and/or abdominal discomfort
- Vasovagal reaction
- Non-sustained hypotension
- Headache
- Sustained bronchospasm or chest tightness

Hematology (Sickle Cell Anemia)

- Anemia and its complications; chronic simple or exchange transfusion in transfusion program for anemia
- Transfusion reactions
- Treatable infections from bacteria, viruses, protozoa and fungi.
- Mild vaso-occlusive crisis requiring oral narcotic analgesia
- Vaso-occlusive crisis requiring hospital admission
- Avascular necrosis of the femoral head
- New leg ulcers

Sildenafil

- Transient minor headache
- Nasal congestion

- Dyspepsia
- Transient visual abnormalities including eyelid edema
- Asymptomatic hypotension

13.7.2 SAEs that Require Expedited Reporting to the IRB

The following serious adverse events will be listed in the consent and reported to the local IRB within 7 days of a death or other life threatening event or 15 days of any other serious adverse event as outlined in the Interim Guidelines for Adverse Event Reporting. Other SAEs not noted herein will be reported to local IRBs per the guidelines of each IRB.

Pulmonary Artery Catheterization

- Conduction abnormalities or atrial/ventricular arrhythmias occurring during cardiac catheterization requiring pacing or cardioversion/defibrillation.
- Injury to coronary artery, myocardium, or great vessel during diagnostic or research cardiac catheterization that requires further treatment; interventionally, surgically or pharmacologically.
- Pericardial effusion following diagnostic/research cardiac catheterization study requiring more intensive monitoring, or treatment such as a pericardial tap.
- Pneumothorax following diagnostic/research cardiac catheterization (left ventricular puncture) requiring more intensive monitoring or insertion of a chest tube.
- Hematoma with pseudoaneurysm (of major artery resulting from inadvertent puncture or of the pulmonary artery) following the catheterization.
- Apnea requiring airway management during conscious sedation for diagnostic cardiac catheterization.
- Myocardial infarction/neurological event during or following (within 24 hours) diagnostic/research cardiac catheterization

Hematology (Sickle Cell Anemia)

- Priapism requiring medical or surgical intervention
- Acute chest syndrome/pneumonia requiring simple or exchange transfusion, admission and antibiotic therapy (not mechanical ventilation)
- Infections from bacteria, viruses, protozoa and fungi requiring vasopressor support or hospital admission
- Avascular necrosis of the femoral head requiring surgical intervention

- Bleeding requiring transfusion
- Major bleeding complication including intracerebral hemorrhage, stroke or GI bleed
- Sustained thrombocytopenia or neutropenia
- New leg ulcers requiring surgical intervention
- Greater than two admissions for vaso-occlusive crisis per week for two sequential weeks
- Retinal detachment or vitreous bleeding
- Pulmonary embolism
- All hospitalizations for medical indications (with the exception of hospitalizations for vaso-occlusive crises – per Section 13.7.1)

Pulmonary Function Testing

- Sustained bronchospasm or chest tightness resulting in mechanical ventilation

Sildenafil

- Hypotension requiring admission or fluid resuscitation
- Visual changes requiring discontinuation of therapy or ophthalmologic evaluation
- Hypotension that requires vasopressor support
- Priapism requiring exchange transfusion or surgical intervention

13.8 Reporting of Serious Safety Issues to the DSMB; Suspension Guidelines

Serious safety issues that arise in this study will be brought to the attention of the Data and Safety Monitoring Board (DSMB), which will make recommendations to the National Heart, Lung, and Blood Institute (NHLBI) regarding possible suspension or termination of the study. The NHLBI will consider the DSMB's recommendations, determine an appropriate action and notify the Data Coordinating Center (DCC). The DCC will notify all participating investigators, who will implement the actions directed by the NHLBI. This section defines "serious safety issues" and describes procedures for bringing them to the attention of the DSMB.

The DCC or PI will make the following types of reports that can alert the DSMB to a potential safety issue:

- Ad hoc reports of unexpected serious adverse events and those serious adverse events associated with major bleeding that are made within 7 or 15 calendar days, as specified in subsequent paragraphs.

- Reports of quarterly statistical analyses of all serious adverse events. The DCC will make such analyses quarterly, but files a report to the DSMB only when analyses indicate that a safety issue has arisen, as defined by the "alert" criteria.
- Reports of semi-annual DCC analyses of all adverse events and of adverse clinical laboratory trends. These reports will highlight any safety issues revealed by the analyses that meet the "alert" criteria.

The DCC will submit the DSMB report to the Chair of the DSMB Subcommittee appointed to monitor this study and to the NHLBI Project Officer and will submit the unexpected SAE report to all study investigators. Each CI will submit the unexpected SAE report to the local Institutional Review Board (IRB) and other local authorities in accordance with the institution's regulations.

13.8.1 Reporting of All Serious Adverse Events to the DSMB

Three months after the first subject is enrolled in the study, and at the end of each 3 month period thereafter, if any SAEs (expected or unexpected) have been reported in the study during the preceding 3 months, the DCC will:

- Use the current version of the MedDRA dictionary to code all AEs (serious or not) that have been recorded on study AE forms.
- Make a "snapshot" copy of the adverse events data, including MedDRA codes.
- Create frequency tables of treatment x occurrence (yes or no, since inception of the study) of all subjects. One table will be created for each highest-level MedDRA term for which SAEs have been reported. The counting units are subjects, not events.
- Compute Fisher's Exact Test (FET) statistic to test the alternative hypothesis that occurrence of SAEs is not independent of treatment group. The FET p-value is not adjusted for multiplicity.
- If the FET p-value is less than the critical value shown in Table 12 and the active treatment group has a higher AE rate, the DCC will conduct further statistical analyses as indicated by the circumstances and report the results to the Chair of the DSMB subcommittee monitoring this study, the PI, and the NHLBI Project Officer.

The DCC will not file a report of all SAEs if none of the FET p-values is less than the critical value shown in Table 12 or if the relative risk is less than 1.

13.8.2 Reporting of All Adverse Events (Serious and Non-serious)

Six months after the first subject is enrolled in the study, and at the end of each 6 month period thereafter, the DCC will:

- Use the current version of the Medical Dictionary for Regulatory Activities (MedDRA) to code all AEs that have been recorded on study AE forms.
- Make a "snapshot" copy of the adverse events data, including MedDRA codes.

- Create frequency tables of treatment x occurrence (yes or no, since inception of the study) of all subjects. One table will be created for each highest-level MedDRA term for which AEs have been reported. The counting units are subjects, not events.
- Compute Fisher's Exact Test (FET) statistic to test the alternative hypothesis that occurrence of AEs is not independent of treatment group. The FET p-value is not adjusted for multiplicity.
- If the FET p-value is less than the critical value shown in Table 12 and the active treatment group has a higher AE rate, the DCC will conduct further statistical analyses as indicated by the circumstances and alert the DSMB to this finding in the semi-annual DSMB report.
- Collaborate with the PI to incorporate the results into the study's semi-annual report to the DSMB and the NHLBI Project Officer.
- A copy of the study's semi-annual report will be submitted to the IND Holder's IRB. Measures will be taken to ensure that the PI and Study Coordinator at that site remain blinded to their subject's treatment.

13.8.3 Reporting of Adverse Clinical Laboratory Trends

Six months after the first subject is enrolled in the study, and at the end of each 6 month period thereafter, the DCC will:

- Make a "snapshot" copy of the study's clinical laboratory data.
- Perform an appropriate statistical analysis of clinical laboratory change-from-baseline data for each clinical laboratory evaluation obtained in this study.
- Perform an appropriate statistical test of H_o: (The mean [or median, or proportion, as appropriate] change-from-baseline of the clinical laboratory values for the active treatment group is the same as for the control group), vs. H_a: (The mean [or median, or proportion] change-from-baseline in the active treatment group is "worse" than for the control group). The meaning of "worse" depends upon the specific clinical lab measurement. The test statistic p-value is not adjusted for multiplicity.
- If the hypothesis tests p-value is less than the critical value shown in Table 12, the DCC will conduct further statistical analyses as indicated by the circumstances and highlight this finding in the semi-annual DSMB report.
- Collaborate with the PI to incorporate the results into the study's semi-annual report to the DSMB and the NHLBI Project Officer.
- A copy of the study's semi-annual report will be submitted to the IND Holder's IRB. Measures will be taken to ensure that the CI and study coordinator at that site remain blinded to their subject's treatment.

Table 12. Summary of Procedures and Timing for Alerting the DSMB and NHLBI Project Officer of Possible Serious Safety Issues

Situation or Event	Summary of Procedure (See text for details.)	Critical Value for DSMB "Alert"
Unexpected SAEs	Clinical investigator notifies DCC SAE Regulatory Specialist and PI.<br>Clinical investigator, DCC SAE Regulatory Specialist and PI prepare report using FDA forms and submit report to DSMB, NHLBI Project Officer, IRBs, study investigators. Report:<br>Fatal or life-threatening: within 7 calendar days.<br>Otherwise: within 15 calendar days.	Alert all cases.
Serious Bleeding Events, other than above	Clinical investigator notifies DCC SAE Regulatory Specialist and PI.<br>Clinical investigator, DCC SAE Regulatory Specialist and PI prepare report and submit report to DSMB and NHLBI Project Officer within 15 calendar days	Alert all cases.
All SAEs	DCC performs quarterly analyses of MedDRA-coded SAEs, tabulates subjects with SAEs classified by highest-level MedDRA term. Report only when p < critical value and active treatment group has higher AE rate.	$p < 0.01$<br>p not adjusted for multiplicity
Adverse Events (all)	DCC performs semi-annual analyses of MedDRA-coded AEs, tabulates subjects with AEs classified by highest-level MedDRA term. Report every 6 months. Alert only when FET p < critical value and active treatment group has higher AE rate.	$p < 0.01$<br>p not adjusted for multiplicity
Adverse Clinical Lab Trends	DCC performs semi-annual analyses of clinical lab change-from-baseline using analyses appropriate for the data type. Report every 6 months. Alert only when p < critical value and change is in "adverse" direction.	$p < 0.005$<br>p not adjusted for multiplicity

13.9 Pregnancy Reporting

Because of potential adverse effects on the fetus, pregnant subjects will be excluded from the study. Female subjects who are pubertal will be regularly assessed throughout the study. In the event of a positive pregnancy test, the subject will be immediately informed of this result and will have sildenafil treatment discontinued.

The investigator, or his/her designee, will collect pregnancy information on any female who becomes pregnant while participating in this study. The investigator or his/her designee will record pregnancy information on the appropriate form and submit it to the DCC Product Safety Associate within 2 weeks of learning of a subject's pregnancy. The

subject will also be followed to determine the outcome of the pregnancy. Information on the status of the mother and child will also be reported to the DCC. Generally follow-up will be no longer than 6 to 8 weeks following the estimated delivery date. Any premature termination of the pregnancy will be reported.

While pregnancy itself is not considered to be an AE or SAE, any pregnancy complication, elective termination of a pregnancy for medical reasons, or spontaneous abortion will be recorded as an AE or a SAE, as appropriate.

13.10 Clinical and Safety Monitoring

13.10.1 Subject Safety Monitoring

The Data and Safety Monitoring Board (DSMB) has been appointed by and is responsible to NHLBI. The DSMB has adopted a written charter that has been approved by NHLBI and will govern its activities. The DSMB will decide how often they need to review the safety data for each protocol. The DCC will provide a report to the DSMB approximately 2 weeks prior to each scheduled DSMB meeting. This report will consist of relevant safety information. Upon review of the safety data, the DSMB will make a recommendation to continue the trial or stop it due to safety concerns.

13.10.2 Clinical Monitoring Plan

A study coordinator from the DCC will assess overall enrollment and compliance with the clinical protocol at all participating sites. A trained clinical research associate (CRA) will visit the sites once every year for the duration of the trial. The CRA will focus on compliance with Good Clinical Practice and data quality and will provide feedback to the sites on how well they are capturing and entering their data.

13.10.3 Plan for Reporting Protocol Violations/Deviations

All protocol violations/deviations will be reported on the DCC Protocol Deviation/Violation Form. This form asks for a description of the event, any corrective action taken, and the final disposition of the event.

14 COMPENSATION

Each study site will determine compensation for subjects participating at that site. The terms of the compensation will be approved by that site’s governing IRB.

15 STUDY CONDUCT

This protocol has been filed under an Investigational New Drug (IND) application with the US Food and Drug Administration (FDA) and will be conducted in accordance with “good clinical practice” (GCP), all applicable US regulatory requirements, and where

applicable, the UK Medicines for Human Use (Clinical Trials) Regulations 2004 (SI 2004/1031) and the European Clinical Trials Directive 2001/20/EC (EUCTD).

16 INVESTIGATOR AGREEMENT

Investigator Agreement: I have read the protocol and agree to conduct the study as outlined herein.

Signature: ________________________________ Date: ________________________

Name (print): ________________________________

17 REFERENCES

1. Castro O. Systemic fat embolism and pulmonary hypertension in sickle cell disease. *Hematology - Oncology Clinics of North America.* 1996;10(6):1289-1303.

2. Sutton LL, Castro O, Cross DJ, Spencer JE, Lewis JF. Pulmonary hypertension in sickle cell disease. *American Journal of Cardiology.* 1994;74(6):626-628.

3. Norris SL, Johnson C, Haywood LJ. Left ventricular filling pressure in sickle cell anemia. *Journal of the Association for Academic Minority Physicians.* 1992;3(1):20-23.

4. Verresen D, De Backer W, Vermeire P. Pulmonary hypertension and sickle hemoglobinopathy. *Chest.* 1990;98(4):1042.

5. Simmons BE, Santhanam V, Castaner A, Rao KR, Sachdev N, Cooper R. Sickle cell heart disease. Two-dimensional echo and Doppler ultrasonographic findings in the hearts of adult patients with sickle cell anemia. *Archives of Internal Medicine.* 1988;148(7):1526-1528.

6. anonymous. Case records of the Massachusetts General Hospital. Weekly clinicopathological exercises. Case 52-1983. Pulmonary hypertension associated with abnormal hemoglobin. *New England Journal of Medicine.* 1983;309(26):1627-1636.

7. Collins FS, Orringer EP. Pulmonary hypertension and cor pulmonale in the sickle hemoglobinopathies. *American Journal of Medicine.* 1982;73(6):814-821.

8. Rich S, Dantzker DR, Ayres SM, et al. Primary pulmonary hypertension. A national prospective study. *Ann Intern Med.* Aug 1987;107(2):216-223.

9. Powars D, Weidman JA, Odom-Maryon T, Niland JC, Johnson C. Sickle cell chronic lung disease: prior morbidity and the risk of pulmonary failure. *Medicine.* 1988;67(1):66-76.

10. Castro O, Hoque M, Brown BD. Pulmonary hypertension in sickle cell disease: cardiac catheterization results and survival. *Blood.* 2003;101(4):1257-1261.

11. Gladwin MT, Sachdev V, Jison ML, et al. Pulmonary hypertension as a risk factor for death in patients with sickle cell disease. *N Engl J Med.* Feb 26 2004;350(9):886-895.

12. Ataga KI, Moore CG, Jones S, et al. Pulmonary hypertension in patients with sickle cell disease: a longitudinal study. *Br J Haematol.* Jul 2006;134(1):109-115.

13. De Castro LM, Jonassiant JC, Graham FL, Ashley-Koch A, Tellen MJ. Pulmonary Hypertension in SS, SC and Sβ Thalassemia: Prevalence, Associated Clinical Sydromes, and Mortality. *Blood.* 16 November 2004 2004;104(11):462a.

14. Gladwin MT, Schechter AN, Shelhamer JH, et al. Inhaled nitric oxide augments nitric oxide transport on sickle cell hemoglobin without affecting oxygen affinity. *Journal of Clinical Investigation.* 1999;104(7):937-945.

15. Reiter CD, Wang X, Tanus-Santos JE, et al. Cell-free hemoglobin limits nitric oxide bioavailability in sickle-cell disease. *Nature Medicine.* 2002;8(12):1383-1389.

16. De Caterina R, Libby P, Peng HB, et al. Nitric oxide decreases cytokine-induced endothelial activation. Nitric oxide selectively reduces endothelial expression of adhesion molecules and proinflammatory cytokines. *J Clin Invest.* Jul 1995;96(1):60-68.

17. Rybicki AC, Benjamin LJ. Increased levels of endothelin-1 in plasma of sickle cell anemia patients. *Blood.* 1998;92(7):2594-2596.

18. Azizi E, Dror Y, Wallis K. Arginase activity in erythrocytes of healthy and ill children. *Clin Chim Acta.* Jun 1970;28(3):391-396.

19. Belfiore F. [Enzymatic Activities of the Blood Serum in Thalassemia and in Thalassodrepanocytosis]. *Riforma Med.* Sep 19 1964;78:1052-1055.

20. Morris CR, Kato GJ, Poljakovic M, et al. Dysregulated arginine metabolism, hemolysis-associated pulmonary hypertension, and mortality in sickle cell disease. *Jama.* Jul 6 2005;294(1):81-90.

21. Morris CR, Kuypers FA, Larkin S, et al. Arginine therapy: a novel strategy to induce nitric oxide production in sickle cell disease. *British Journal of Haematology.* 2000;111(2):498-500.

22. Schnog JJ, Jager EH, van der Dijs FP, et al. Evidence for a metabolic shift of arginine metabolism in sickle cell disease. *Ann Hematol.* Jun 2004;83(6):371-375.

23. Kato GJ MV, Machado RF, Little JA, Taylor J 6th, Morris CR, Nichols JS, Wang X, Poljakovic M, Morris SM Jr, Gladwin MT. Lactate dehydrogenase as a biomarker of hemolysis-associated nitric oxide resistance, priapism, leg ulceration, pulmonary hypertension, and death in patients with sickle cell disease. *Blood.* 2006;107(6):2279-2285.

24. Galie N, Ghofrani HA, Torbicki A, et al. Sildenafil citrate therapy for pulmonary arterial hypertension. *N Engl J Med.* Nov 17 2005;353(20):2148-2157.

25. Ghofrani HA, Rose F, Schermuly RT, et al. Oral sildenafil as long-term adjunct therapy to inhaled iloprost in severe pulmonary arterial hypertension. *J Am Coll Cardiol.* Jul 2 2003;42(1):158-164.

26. Ghofrani HA, Schermuly RT, Rose F, et al. Sildenafil for long-term treatment of nonoperable chronic thromboembolic pulmonary hypertension. *Am J Respir Crit Care Med.* Apr 15 2003;167(8):1139-1141.

27. Ghofrani HA, Wiedemann R, Rose F, et al. Combination therapy with oral sildenafil and inhaled iloprost for severe pulmonary hypertension. *Ann Intern Med.* Apr 2 2002;136(7):515-522.

28. Ghofrani HA, Wiedemann R, Rose F, et al. Sildenafil for treatment of lung fibrosis and pulmonary hypertension: a randomised controlled trial. *Lancet.* Sep 21 2002;360(9337):895-900.

29. Hoeper MM, Olschewski H, Ghofrani HA, et al. A comparison of the acute hemodynamic effects of inhaled nitric oxide and aerosolized iloprost in primary pulmonary hypertension. German PPH study group. *J Am Coll Cardiol.* Jan 2000;35(1):176-182.

30. Lepore JJ, Maroo A, Pereira NL, et al. Effect of sildenafil on the acute pulmonary vasodilator response to inhaled nitric oxide in adults with primary pulmonary hypertension. *Am J Cardiol.* Sep 15 2002;90(6):677-680.

31. Michelakis E, Tymchak W, Lien D, Webster L, Hashimoto K, Archer S. Oral sildenafil is an effective and specific pulmonary vasodilator in patients with

pulmonary arterial hypertension: comparison with inhaled nitric oxide. *Circulation.* May 21 2002;105(20):2398-2403.

32. Michelakis ED, Tymchak W, Noga M, et al. Long-term treatment with oral sildenafil is safe and improves functional capacity and hemodynamics in patients with pulmonary arterial hypertension. *Circulation.* Oct 28 2003;108(17):2066-2069.

33. Driscoll MC, Hurlet A, Styles L, et al. Stroke risk in siblings with sickle cell anemia. *Blood.* Mar 15 2003;101(6):2401-2404.

34. Therneau TM. How many stratification factors are 'too many' to use in a randomization plan? *Controlled Clinical Trials.* 1993;14:98-108.

35. Begg CB, Iglewicz B. A treatment allocation procedure for sequential clinical trials. *Biometrics.* 1980;36:81-90.

36. Pocock SJ, Simon R. Sequential treatment assignment with balancing for prognostic factors in the controlled clinical trial. *Biometrics.* 1975;31:103-115.

37. Freedman LS, White SJ. On the use of Pocock and Simon's method for balancing treatment numbers over prognostic factors in the controlled clinical trial. *Biometrics.* 1976;32:691-694.

38. Guyatt GH, Sullivan MJ, Thompson PJ, et al. The 6-minute walk: a new measure of exercise capacity in patients with chronic heart failure. *Can Med Assoc J.* Apr 15 1985;132(8):919-923.

39. Barst RJ, Rubin LJ, Long WA, et al. A comparison of continuous intravenous epoprostenol (prostacyclin) with conventional therapy for primary pulmonary hypertension. The Primary Pulmonary Hypertension Study Group. *N Engl J Med.* Feb 1 1996;334(5):296-302.

40. Badesch DB, Tapson VF, McGoon MD, et al. Continuous intravenous epoprostenol for pulmonary hypertension due to the scleroderma spectrum of disease. A randomized, controlled trial. *Ann Intern Med.* Mar 21 2000;132(6):425-434.

41. Kato GJ, Onyekwere OC, Gladwin MT. PULMONARY HYPERTENSION IN SICKLE CELL DISEASE: Relevance to Children. *Pediatr Hematol Oncol.* Apr-May 2007;24(3):159-170.

42. Baquero H, Soliz A, Neira F, Venegas ME, Sola A. Oral sildenafil in infants with persistent pulmonary hypertension of the newborn: a pilot randomized blinded study. *Pediatrics.* Apr 2006;117(4):1077-1083.

43. Barnett CF, Machado RF. Sildenafil in the treatment of pulmonary hypertension. *Vasc Health Risk Manag.* 2006;2(4):411-422.

44. Brancaccio G, Toscano A, Bevilacqua M, Di Chiara L, Parisi F. Bosentan and sildenafil: should the combination therapy be a valid alternative in childhood to prostacyclin infusion? *Pediatr Transplant.* Feb 2007;11(1):110-112.

45. Chaudhari M, Vogel M, Wright C, Smith J, Haworth SG. Sildenafil in neonatal pulmonary hypertension due to impaired alveolarisation and plexiform pulmonary arteriopathy. *Arch Dis Child Fetal Neonatal Ed.* Nov 2005;90(6):F527-528.

46. Hamdan MA, Abu-Sulaiman RM, Najm HK. Sildenafil in pulmonary hypertension secondary to unilateral agenesis of pulmonary artery. *Pediatr Cardiol.* Mar-Apr 2006;27(2):279-281.

47. Keller RL, Moore P, Teitel D, Hawgood S, McQuitty J, Fineman JR. Abnormal vascular tone in infants and children with lung hypoplasia: Findings from cardiac catheterization and the response to chronic therapy. *Pediatr Crit Care Med.* Nov 2006;7(6):589-594.

48. Lammers AE, Haworth SG, Pierce CM. Intravenous sildenafil as an effective treatment of pulmonary hypertensive crises during acute intestinal malabsorption. *Cardiol Young.* Feb 2006;16(1):84-86.

49. Leibovitch L, Matok I, Paret G. Therapeutic applications of sildenafil citrate in the management of paediatric pulmonary hypertension. *Drugs.* 2007;67(1):57-73.

50. Lunze K, Gilbert N, Mebus S, et al. First experience with an oral combination therapy using bosentan and sildenafil for pulmonary arterial hypertension. *Eur J Clin Invest.* Sep 2006;36 Suppl 3:32-38.

51. Martell M, Blasina F, Silvera F, et al. Intratracheal sildenafil in the newborn with pulmonary hypertension. *Pediatrics.* Jan 2007;119(1):215-216; author reply 216.

52. Nagdyman N, Fleck T, Bitterling B, et al. Influence of intravenous sildenafil on cerebral oxygenation measured by near-infrared spectroscopy in infants after cardiac surgery. *Pediatr Res.* Mar 2006;59(3):462-465.

53. Namachivayam P, Theilen U, Butt WW, Cooper SM, Penny DJ, Shekerdemian LS. Sildenafil prevents rebound pulmonary hypertension after withdrawal of nitric oxide in children. *Am J Respir Crit Care Med.* Nov 1 2006;174(9):1042-1047.

54. Nemoto S, Umehara E, Ikeda T, Itonaga T, Komeda M. Oral sildenafil ameliorates impaired pulmonary circulation early after bidirectional cavopulmonary shunt. *Ann Thorac Surg.* May 2007;83(5):e11-13.

55. Noori S, Friedlich P, Wong P, Garingo A, Seri I. - Cardiovascular Effects of Sildenafil in Neonates and Infants with Congenital Diaphragmatic Hernia and Pulmonary Hypertension. 2007;- 91(- 2):- 100.

56. Oliveira EC, Amaral CF. [Sildenafil in the management of idiopathic pulmonary arterial hypertension in children and adolescents]. *J Pediatr (Rio J).* Sep-Oct 2005;81(5):390-394.

57. Rugolotto S, Errico G, Beghini R, Ilic S, Richelli C, Padovani EM. Weaning of epoprostenol in a small infant receiving concomitant bosentan for severe pulmonary arterial hypertension secondary to bronchopulmonary dysplasia. *Minerva Pediatr.* Oct 2006;58(5):491-494.

58. Simiyu DE, Okello C, Nyakundi EG, Tawakal AH. Sildenafil in management of persistent pulmonary hypertension of the newborn: report of two cases. *East Afr Med J.* Jun 2006;83(6):337-340.

59. Singh TP, Rohit M, Grover A, Malhotra S, Vijayvergiya R. A randomized, placebo-controlled, double-blind, crossover study to evaluate the efficacy of oral sildenafil therapy in severe pulmonary artery hypertension. *Am Heart J.* Apr 2006;151(4):851 e851-855.

60. Tulloh R. Management and therapeutic options in pediatric pulmonary hypertension. *Expert Rev Cardiovasc Ther.* May 2006;4(3):361-374.

61. Uzun O, Wong JK, Bhole V, Stumper O. Resolution of protein-losing enteropathy and normalization of mesenteric Doppler flow with sildenafil after Fontan. *Ann Thorac Surg.* Dec 2006;82(6):e39-40.

62. Wong AR, Rasool AH, Abidin NZ, Noor AR, Quah BS. Sildenafil as treatment for Human Immunodeficiency Virus-related pulmonary hypertension in a child. *J Paediatr Child Health.* Mar 2006;42(3):147-148.

63. Humpl T, Reyes JT, Holtby H, Stephens D, Adatia I. Beneficial effect of oral sildenafil therapy on childhood pulmonary arterial hypertension: twelve-month clinical trial of a single-drug, open-label, pilot study. *Circulation.* Jun 21 2005;111(24):3274-3280.

64. Gladwin MT, Slonim A, Landucci DL, Gutierrez DC, Cunnion RE. Cannulation of the internal jugular vein: is postprocedural chest radiography always necessary? *Crit Care Med.* Sep 1999;27(9):1819-1823.

65. Wheeler AP, Bernard GR, Thompson BT, et al. Pulmonary-artery versus central venous catheter to guide treatment of acute lung injury. *N Engl J Med.* May 25 2006;354(21):2213-2224.

66. Hayreh SS. Erectile dysfunction drugs and non-arteritic anterior ischemic optic neuropathy: is there a cause and effect relationship? *J Neuroophthalmol.* Dec 2005;25(4):295-298.

67. McGwin G, Jr., Vaphiades MS, Hall TA, Owsley C. Non-arteritic anterior ischaemic optic neuropathy and the treatment of erectile dysfunction. *Br J Ophthalmol.* Feb 2006;90(2):154-157.

68. Gorkin L, Hvidsten K, Sobel RE, Siegel R. Sildenafil citrate use and the incidence of nonarteritic anterior ischemic optic neuropathy. *Int J Clin Pract.* Apr 2006;60(4):500-503.

69. Fraunfelder FW, Shults T. Non-arteritic anterior ischemic optic neuropathy, erectile dysfunction drugs, and amiodarone: is there a relationship? *J Neuroophthalmol.* Mar 2006;26(1):1-3.

70. Gillies HC, Roblin D, Jackson G. Coronary and systemic hemodynamic effects of sildenafil citrate: from basic science to clinical studies in patients with cardiovascular disease. *Int J Cardiol.* Dec 2002;86(2-3):131-141.

71. Carlsen J, Kjeldsen K, Gerstoft J. Sildenafil as a successful treatment of otherwise fatal HIV-related pulmonary hypertension. *Aids.* Jul 26 2002;16(11):1568-1569.

72. Jackson G, Chambers J. Sildenafil for primary pulmonary hypertension: short and long-term symptomatic benefit. *Int J Clin Pract.* Jun 2002;56(5):397-398.

73. Kothari SS, Duggal B. Chronic oral sildenafil therapy in severe pulmonary artery hypertension. *Indian Heart J.* Jul-Aug 2002;54(4):404-409.

74. Littera R, La Nasa G, Derchi G, Cappellini MD, Chang CY, Contu L. Long-term treatment with sildenafil in a thalassemic patient with pulmonary hypertension. *Blood.* Aug 15 2002;100(4):1516-1517.

75. Sastry BK, Narasimhan C, Reddy NK, et al. A study of clinical efficacy of sildenafil in patients with primary pulmonary hypertension. *Indian Heart J.* Jul-Aug 2002;54(4):410-414.

76. Zimmermann AT, Calvert AF, Veitch EM. Sildenafil improves right-ventricular parameters and quality of life in primary pulmonary hypertension. *Intern Med J.* Aug 2002;32(8):424-426.

77. Watanabe H, Ohashi K, Takeuchi K, et al. Sildenafil for primary and secondary pulmonary hypertension. *Clin Pharmacol Ther.* May 2002;71(5):398-402.

78. Singh B, Gupta R, Punj V, et al. Sildenafil in the management of primary pulmonary hypertension. *Indian Heart J.* May-Jun 2002;54(3):297-300.

79. Abrams D, Schulze-Neick I, Magee AG. Sildenafil as a selective pulmonary vasodilator in childhood primary pulmonary hypertension. *Heart.* Aug 2000;84(2):E4.

80. Bialecki ES, Bridges KR. Sildenafil relieves priapism in patients with sickle cell disease. *Am J Med.* Aug 15 2002;113(3):252.

18 APPENDICES

This protocol sponsored by the
National Heart Lung and Blood Institute

Appendix A: Echocardiographic parameters

LV Structure and Function	LVED size (mm)<br>LVES size (mm)<br>Septal thickness (mm)<br>PW thickness (mm)<br>LVMI (m/h)<br>LVEF (%)
LV Diastolic Function	Left atrial area (cm^2)<br>Max LA vol/BSA (ml/m^2)<br>Peak E velocity (m/s)<br>Peak A velocity (m/s)<br>E/A ratio<br>Deceleration time (msec)<br>Isovolumic relaxation time (msec)<br>Pulmonary vein flow<br>Tissue Doppler of the septum and lateral walls
Right Heart Size and Function	Right atrial area (cm^2)<br>RV end diastolic area (cm2)<br>RV end systolic area (cm2)<br>RV area change (%)<br>RV:LV diastolic area ratio<br>Tricuspid regurgitant jet velocity (m/s)
Valvular Function	

This protocol sponsored by the
National Heart Lung and Blood Institute

Appendix B: Patient History Intake Document

Date of visit:
History and Physical exam and significant findings
Chief Complaint:
History of the Present Illness:
Past Medical History / Review of Systems:
Allergies:

HOSPITALIZATIONS
Date of last hospitalization:
Reason for admission:

Number of hospitalizations in last year:
TRANSFUSIONS
Total number of transfusions in lifetime: ☐ 0 ☐ 1-4 ☐ 5-10 ☐ >10
Date of last transfusion:
Type of transfusion and number of units:
Known antibodies? Y N
History of transfusion reaction? Y N, if yes describe:

PAIN
Description of pain (include location, quality, rating on a scale of 1-10, and treatment):
Chronic

Acute

# of MILD pain crises (events) in the last week:_____, in the last month: ______, in the last year: ______(may or may not require pain medicine and did not prevent normal daily activity)

# of MODERATE pain crises (events) in the last week:_____, in the last month: ______, in the last year: ________(required medications and caused significant changes in daily activities (ie. missing work)

# of SEVERE pain crises (events) in the last week :_____, in the last month: _______, in the last year: ________(went to ER but was not admitted)

# of EXTREMELY SEVERE pain crises (events) in the last week:_____, in the last month: ______, in the last year: _________(admitted to the hospital)

MEDICAL HISTORY OF: (please circle and comment where appropriate)

Y N Hip complications/Avascular necrosis
Y N Stroke/TIA
Y N Headaches
Y N Retinal disease
Y N Asthma or wheezing
Y N Pneumonia or acute chest syndrome
Date of last episode
Treatment
Number of episodes total
Number that required ICU care
Number that required blood transfusions

Y N Heart failure
Y N Heart attack
Y N Arrhythmias
Y N Enlarged heart
Y N Obstructive sleep apnea
Y N Chronic lung disease
Y N Hepatitis
Y N Gallbladder disease
Y N Sickle hepatopathy
Y N Renal insufficiency
Y N Hemo or peritoneal dialysis
Y N Hematuria
Y N Nocturia
Y N Skin ulcers
Treatment
duration
Y N Iron overload
Y N Adrenal insufficiency
Y N Other:

Reproductive: Female

- Age menarche
- Age of onset of menopause
- LMP
- Cycle length
- Regular Y N
- G P

Reproductive: Male

- Priapism Y N
- Treatment

PAST SURGICAL HISTORY

Y N Splenectomy
Y N Cholecystectomy
Y N Hip replacement
Y N Shoulder replacement
Y N Laser eye surgery
Y N Port/VAD
Y N Other:

SOCIAL HISTORY

- Smoking (include # packs per day and date of smoking cessation):
- Alcohol / Drugs:
- Living arrangements:
- Employment:
- Social work: does patient want to be contacted by social work?

FAMILY HISTORY

Y N Other family members with sickle cell disease?

PERSONAL PHYSICIAN CONTACT INFORMATION:

MEDICATIONS

- ❑ Hydroxyurea
- ❑ Folic acid

- ❑ Dilaudid
- ❑ Oxycodone
- ❑ Oxycontin
- ❑ Codeine
- ❑ Tylenol 3
- ❑ Morphine
- ❑ Demerol
- ❑ Ibuprofen
- ❑ Percocet
- ❑ Vicodin
- ❑ Other:

Physical Exam:

Vitals:	P	R	B/P	T	SPO2
	Ht	Wt			

General Appearance:
HEENT:
NEURO
CARDIOVASCULAR
Clubbing: absent mild mod severe
PULMONARY
GI:
SKIN

Appendix C: Acute inhaled NO and sildenafil challenge at Baseline and End of MIT

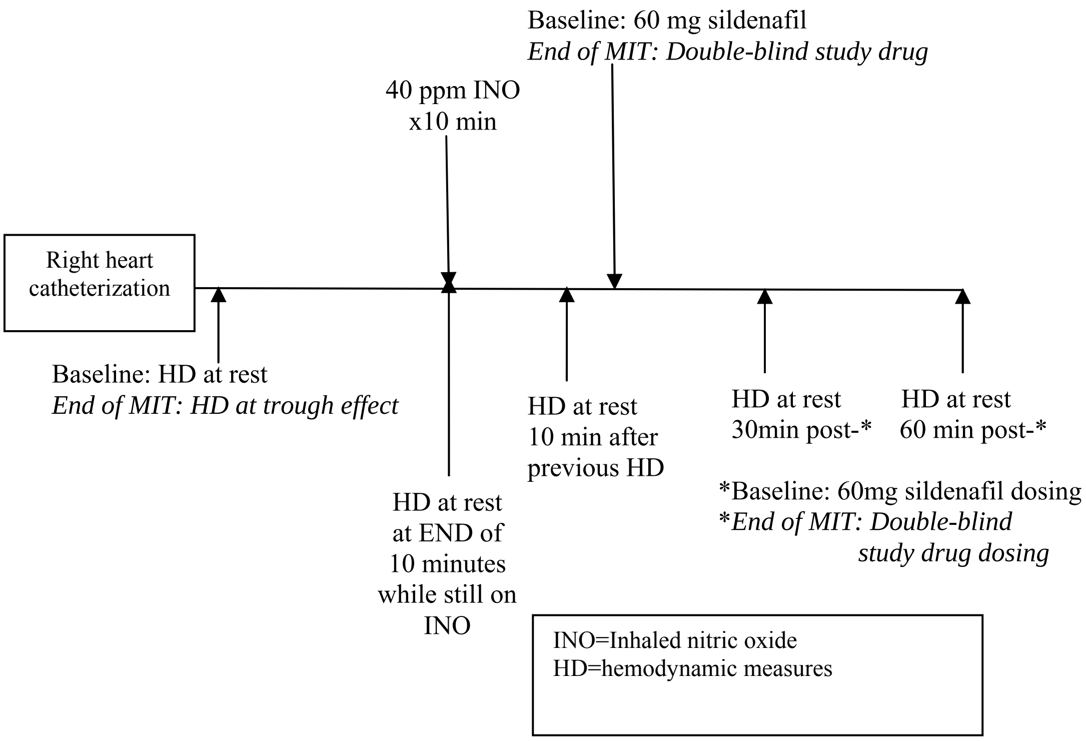

Appendix D: Six Minute Walk Test

The **unencouraged** 6 minute walk test will be used. The test should be administered by the same tester throughout the study. The timing for all walk tests should be 1 to 2 hours after taking study drug medication. The test should be conducted in a corridor on a course with a predetermined length, e.g. 30 m and at least 6-10 feet in width. The area should be well ventilated with temperature controlled at 20-23 °C (68-76 °F). If the subject was using oxygen for his Baseline walk test, subsequent walk tests should be performed under the same conditions.

The tester may be at the starting end of the corridor or at the midpoint of the corridor with a stop-watch. Intermittent rest periods are allowed if the subject can no longer continue. If the subject needs to rest briefly, he may stand or lean against the wall and then begin walking again when able; the clock will continue to run.

Subjects will be instructed that the preceding meal should be light. They will be told to wear comfortable clothing and shoes. The person administering the test will use the following exact dialogue with the subject: “The object of this test is to walk as far as possible for 6 minutes. You will walk back and forth in this hallway. Six minutes is a long time to walk, so you will be exerting yourself. You will probably get out of breath or become exhausted. You are permitted to slow down, to stop, and to rest as necessary. You may lean against the wall while resting, but resume walking as soon as you are able. You will be walking back and forth around the cones. You should pivot briskly around the cones and continue back the other way without hesitation. Now I am going to show you. Please watch the way I turn without hesitation.”
Demonstrate by walking one lap yourself. Walk and pivot around the cone briskly

“Are you ready to do that? I am going to use this counter to keep track of the number of laps you complete. I will click it each time you turn around at this starting line. Remember that the object is to walk AS FAR AS POSSIBLE for 6 minutes, but don’t run or jog.”

After these instructions are given, the tester will then say: “I will tell you the time, and I will let you know when 6 minutes are up. When I say STOP, please stand right where you are. Do you have any questions about the test? Please explain what you are going to do.”

The tester will then start the test by saying the following: “Are you ready? Start when I say GO”. The tester will tell the subject the time at 2 and 4 minutes by saying, “You have completed 2 minutes” and “You have completed 4 minutes.” **No other instruction or encouragement should be given during the test**. Eye contact with the subject should be avoided during the test. At the end of 6 minutes, the tester will call “stop” while simultaneously stopping the watch; the distance walked will be measured. The Borg dyspnea score will then be recorded (Appendix 3) by asking,
“Please grade your level of the greatest degree of breathlessness you experienced at any time during the 6-minute walk test, using this scale.”

Table 13. Modified NYHA Functional Classification

**Class I**	Patients with pulmonary hypertension but without resulting limitation of physical activity. Ordinary physical activity does not cause undue dyspnea or fatigue, chest pain, or near syncope.
**Class II**	Patients with pulmonary hypertension resulting in slight limitation of physical activity. They are comfortable at rest. Ordinary physical activity causes undue dyspnea or fatigue, chest pain, or near syncope.
**Class III**	Patients with pulmonary hypertension resulting in marked limitation of physical activity. They are comfortable at rest. Less than ordinary physical activity causes undue dyspnea or fatigue, chest pain, or near syncope.
**Class IV**	Patients with pulmonary hypertension resulting in the inability to carry out any physical activity without symptoms. These patients manifest signs of right heart failure. Dyspnea and/or fatigue may be present at rest, and discomfort is increased by any physical activity.

This protocol sponsored by the
National Heart Lung and Blood Institute

Table 14. Borg dyspnea score

SCALE	SEVERITY OF BREATHLESSNESS
0	Nothing At All
0.5	Very Very Slight (Just Noticeable)
1	Very Slight
2	Slight
3	Moderate
4	Somewhat Severe
5	Severe
6	
7	Very Severe
8	
9	Very Very Severe (Almost Maximum)
10	Maximum

Borg GA. Psychophysical bases of perceived exertion.

Med Sci Sports Exerc 1982; 14:377–381.

Appendix E: Candidate Genes

Table 15. Candidate Genes in Cell Adhesion Molecules

Locus ID	Gene Symbol	Cytogenetic Location
948	CD36	7q11.2
3676	*ITGA4*	2q31-q32
3685	*ITGAV*	2q31-q32
3688	*ITGB1*	10p11.2
3383	*ICAM1*	19p13.3-p13.2
6401	*SELE*	1q22-q25
6402	*SELL*	1q23-q25
6403	*SELP*	1q22-q25
7057	*THBS1*	15q15
7412	*VCAM1*	1p32-p31

Table 16. Candidate Genes - Cytokine and Regulatory Molecules

Locus ID	Gene Symbol	Cytogenetic Location
3458	IFNG	12q14
3459	*IFNGR1*	6q23-q24
3552	*IL1A*	2q14
3557	*IL1RN*	2q14.2
3553	*IL1B*	2q14
3558	*IL2*	4q26-q27
3562	*IL3*	5q31.1
3565	*IL4*	5q31.1
3567	*IL5*	5q31.1
3569	*IL6*	7q21
3576	*IL8*	4q13-q21
3586	*IL10*	1q31-q32
3592	*IL12A*	3p12-p13.2
3593	*IL12B*	5q31.1-q33.1
3596	*IL13*	5q31
1234	*CCR5*	3p21
4049	*LTA*	6p21.3
4050	*LTB*	6p21.3
7124	*TNF*	6p21.3
4843	*NOS2A*	17q11.2-q12
4846	*NOS3*	7q36
3162	*HMOX1*	22q13.1
3163	*HMOX2*	16p13.3

Table 17. Candidate Genes – Major Human Erythrocyte Membrane Proteins

LocusID	Gene Symbol	Description	Position	Location in Membrane
60	ACTB	β-Actin	7p15-p12	peripheral
118	*ADD1*	α-Adducin 1	4p16.3	
119	*ADD2*	β-Adducin 2	2p14-p13	
286	*ANK1*	Ankyrin 1, erythrocytic	8p21.1-p11.2	peripheral
11846	*ARG1*	Arginase	6q23	
11847	*ARG2*	Arginase II	14q24.1-q24.3	
115500	*CAT*	Catalase	11p13	
2035	*EPB41*	Erythrocyte membrane protein band 4.1	1p33p32	peripheral
2038	*EPB42*	Erythrocyte membrane protein band 4.2	15q15-q21	peripheral
2039	*EBP49*	Erythrocyte membrane protein 4.9 (dematin)	8p21.1	peripheral
2040	EPB72	Erythrocyte membrane protein band 7.2 (Stomatin)	9q34.1	integral
2597	GAPD	Glyceraldehyde 3 phosphate dehydrogenase (G3PD)	12p13	peripheral
2993	GYPA	Glycophorin A (MN blood group)	4q28.2-q31.1	integral
2294	*GYPB*	Glycophorin B (Ss blood group)	4q28-q31	integral
2295	*GYPC*	Glycophorin C (Gerbich blood group)	2q14 q21	integral
2296	GYPE	Glycophorin E	4q28-q31	integral
4354	*MPP1*	p55, membrane protein, palmitoylated 1 (55 kd)	Xq28	peripheral
6521	SLC4A1	Solute carrier family 4, anion exchanger, member 1 (band 3)	17q12-q21	integral
6708	SPTA1	α-Spectrin, erythrocytic	1q21	peripheral
6710	*SPTB*	β-Spectrin, erythrocytic	14q23-q24.2	peripheral
7111	*TMOD*	Tropomodulin	9q22.3	peripheral
7170	*TPM3*	Tropomyosin 3	1q22-q23	peripheral

Table 18. Candidate Genes Identified By Gene Expression Analysis in SCD

LocusID	Gene Symbol	Description	Position
3240	HP	haptoglobin	16q22.1
644	*BLVRA*	Biliverdin reductasse A	7p14
645	*BLVRB*	Biliverdin reductase B	19q13.1-q13.2
1026	*CDKN1A*	p21 or cyclin-dependent kinase inhibitor 1A	6p21.2
596	*BCL2*	Adenovirus E1B 19 kDa interacting protein	18q21.33
9332	*CD163*	CD163 antigen	12p13
4153	*MBL2*	mannose binding lectin	10q11.2-q21
9963	SLC23A1	solute carrier family 23 (nucleobase transporters), member 1	5q31.2-q31.3
9962	SLC23A2	solute carrier family 23 (nucleobase transporters), member 2	20p13
